# Self‐Catalyzed Chemically Coalescing Liquid Metal Emulsions

**DOI:** 10.1002/advs.202413116

**Published:** 2025-04-26

**Authors:** Stephanie F. Zopf, Ramón E. Sánchez Cruz, Chloe Kekedjian, Lu Ping, Javier M. Morales Ferrer, Jean Paul Soto Aquino, Rongxuan Xie, Xi Ling, J. William Boley

**Affiliations:** ^1^ Department of Mechanical Engineering Boston University 730 Commonwealth Avenue Boston MA 02215 USA; ^2^ Department of Materials Science Boston University 730 Commonwealth Avenue Boston MA 02215 USA; ^3^ Department of Chemistry Boston University 590 Commonwealth Avenue Boston MA 02215 USA

**Keywords:** catalysis, chemical coalescence, chemical sintering, conductive inks, emulsions, hybrid 3D printing, liquid metals

## Abstract

Gallium‐based liquid metal alloys (GaLMAs) have widespread applications ranging from soft electronics, energy devices, and catalysis. GaLMAs can be transformed into liquid metal emulsions (LMEs) to modify their rheology for facile patterning, processing, and material integration for GaLMA‐based device fabrication. One drawback of using LMEs is reduced electrical conductivity owing to the oxides that form on the surface of dispersed liquid metal droplets. LMEs thus need to be activated by coalescing liquid metal droplets into an electrically conductive network, which usually involves techniques that subject the LME to harsh conditions. This study presents a way to coalesce these droplets through a chemical reaction at mild temperatures (*T* ∼ 80 °C). Chemical activation is enabled by adding halide compounds into the emulsion that chemically etch the oxide skin on the surface of dispersed droplets of eutectic gallium indium (eGaIn). LMEs synthesized with halide activators can achieve electrical conductivities close to bulk liquid metal (2.4 × 10^4^ S cm^−1^) after being heated. 3D printable chemically coalescing LME ink formulations are optimized by systematically exploring halide activator type and concentration, along with mixing conditions, while maximizing for electrical conductivity, shape retention, and compatibility with direct ink writing (DIW). The utility of this ink is demonstrated in a hybrid 3D printing process to create a battery‐integrated light emitting diode array, followed by a nondestructive low temperature heat activation that produces a functional device.

## Graphical abstract

A new type of liquid metal emulsion is developed that chemically activates and becomes electrically conductive under mild thermal stimulus. The activation mechanism occurs through a unique set of catalytic dehalogenation and chemical oxide etching reactions. These emulsions are 3D printable and exhibit high shape retention after being heated. This technology enables simple fabrication of liquid metal‐based applications and patterning of soft metal catalysts.

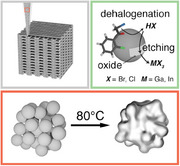

## 1 Introduction

Gallium‐based liquid metal alloys (GaLMAs) are an indispensable class of materials inspiring work in myriad research domains. Two examples of widely researched GaLMAs include eutectic gallium‐indium (eGaIn, 75 w/w% Ga and 25 w/w% In)^[^ ^1^ ^]^ and gallium‐indium‐tin (GaInSn, 68.5 w/w% Ga, 21.5 w/w% In and 10 w/w% Sn).^[^ ^2^ ^]^ The bulk liquid has a low viscosity (eGaIn, 2.0 × 10^−3^ Pa·s^[^ ^3^ ^]^; GaInSn, 2.4 × 10^−3^ Pa·s^[^ ^4^ ^]^) and a high electrical conductivity (eGaIn, 3.4 × 10^4^ S cm^−1^^[^ ^5^ ^]^; GaInSn, 3.46 × 10^4^ S cm^−1^^[^ ^2^ ^]^), making it an ideal deformable conductor for soft electronics.^[^ ^6^ ^]^ Its high apparent surface tension (eGaIn, 624 × 10^−3^ N m^−1^^[^ ^5^ ^]^; GaInSn, 718 × 10^−3^ N m^−1^^[^ ^4^ ^]^) from the fast‐forming oxide skin in oxygenated environments can be electrically^[^ ^7^ ^]^ or chemically^[^ ^8^ ^]^ manipulated for soft actuator applications.^[^ ^9^ ^]^ Even the energy entailed from spontaneous formation of the oxide can be used to power electronics.^[^ ^10^ ^]^ GaLMAs are “atomically intelligent” and can facilitate chemical processes by dissolving other metals in its bulk.^[^ ^11^ ^]^ GaLMAs can also coordinate with other atoms through the ionized metal and oxygen sites on their surface, catalyzing or initiating many chemical reactions.^[^ ^11^ ^]^

Many applications utilize GaLMAs in the form of liquid metal emulsions (LMEs), which involve dispersing the liquid metal droplets in a carrier liquid.^[^ ^12^ ^]^ Because gallium has low toxicity,^[^ ^13^ ^]^ GaLMAs can be processed into functionalized particles for drug delivery and therapeutics.^[^ ^14^ ^]^ When liquid metals are dispersed in elastomer precursors, they form composites with increased fracture toughness, electrical permittivity, and thermal conductivity.^[^ ^15^ ^]^ More recently, liquid metal droplets have been shown to provide a reaction medium to efficiently produce high entropy alloy nanoparticles at bulk scale.^[^ ^16^ ^]^ Additionally, tuning liquid metal concentration in LMEs enables improved rheology for handling and patterning liquid metals, which is essential for fabricating GaLMA‐based devices. Dilute (1–3 v/v%) LMEs produced through sonication (droplet diameters ∼1 nm – 1 µm) can form 2D films through ink‐jet^[^ ^17^ ^]^ or aerosol^[^ ^18^ ^]^ printing, while highly concentrated (60–83 v/v%) LMEs produced through shear mixing (droplet diameters ∼1–200 µm) become viscoelastic pastes with rheology that is compatible with direct ink write (DIW) 3D printing.^[^ ^19^, ^20^, ^21^, ^22^ ^]^ Sedimentation can occur if droplets are not sufficiently stabilized, which may result in nozzles clogging during printing^[^ ^18^ ^]^ or inhomogeneous composites.^[^ ^20^, ^23^, ^24^ ^]^ To address this, droplets can be stabilized with functional ligands,^[^ ^17^, ^25^ ^]^ which work for dilute emulsions. LMEs can also be made into stable dense emulsions^[^ ^21^ ^]^ with volume concentrations above ∼62% (random close packing of spheres).^[^ ^26^ ^]^ While separated and stabilized droplets are desirable for patterning GaLMAs, disconnected liquid metal droplets result in poorly connected liquid metal networks, which is not favorable for applications require liquid metals to perform as deformable conductors.

Sintering or activation is a secondary processing step for making LMEs electrically conductive. During activation, the oxide skin on the dispersed liquid metal droplets must be removed to induce droplet coalescence and create a conductive liquid metal network. Mechanical activation has been used on LMEs in the form of compression^[^ ^17^, ^18^, ^20^, ^27^ ^]^ or tension,^[^ ^21^, ^22^, ^27^, ^28^ ^]^ applying localized stresses or large global stresses to force droplets to coalesce. Laser ablation has also been used to activate LMEs, causing the oxide shell to vaporize, releasing the liquid metal core in each droplet.^[^ ^29^, ^30^ ^]^ Researchers have also improved electrical conductivity by heating LMEs at extreme temperatures (*T* ∼ 500–1000 °C)^[^ ^31^, ^32^ ^]^ to form a new metal oxide/metal biphasic system, or by supercooling LMEs (*T* ∼ −30 °C),^[^ ^22^ ^]^ which causes the frozen bulk liquid metal core in the droplets to expand, breaking the oxide, thus inducing coalescence. Acoustic fields can also activate LMEs, causing nano‐sized liquid metal particles to grow on dispersed LME droplets, subsequently creating droplet connection and forming an electrically conductive percolating pathway.^[^ ^33^ ^]^ While these activation modes succeed in coalescing droplets and rendering patterned emulsions electrically conductive, they present harsh conditions to the other soft and rigid components in GaLMA‐based devices, which can cause unintentional stress and ultimately lead to device failure.

Here we present an LME that activates using a chemical reaction. Our technology takes advantage of the unique ability of halide compounds to chemically etch metal oxides under mild temperatures. Seeking to make our LME viscoelastic with a high stiffness, eGaIn is dispersed at a high‐volume concentration of 80% into a continuous phase liquid mixture of diphenyl ether, rosin and halide‐based activator. The combination of low vapor pressure from diphenyl ether and emulsifying properties of rosin makes the emulsion stable against sedimentation. Through extensive screening of halide activator type and concentration, we arrive at a formulation that optimizes for high electrical conductivity, shape retention, and compatibility with both stencil patterning and direct ink writing (DIW). Rheology for our optimum ink shows that our ink is shear thinning and shear yielding and exhibits a high plateau modulus and high yield stress. As a result, it is able to span millimeter long distances and retain its shape with minimal shrinkage after chemical activation. We demonstrate its utility by employing it as a conductive ink in a hybrid 3D printing process that integrates off the shelf components, including batteries and light emitting diodes (LEDs) in a single auotmated process. The entire fabricated circuit can be activated under mild conditions, thus simplifying and enabling the rapid assembly of liquid metal‐based electronic devices.

## 2 Results and Discussion

### 2.1 Chemical Coalescence

#### 2.1.1 Materials for Enabling Chemical Coalescence

**Figure 1:**
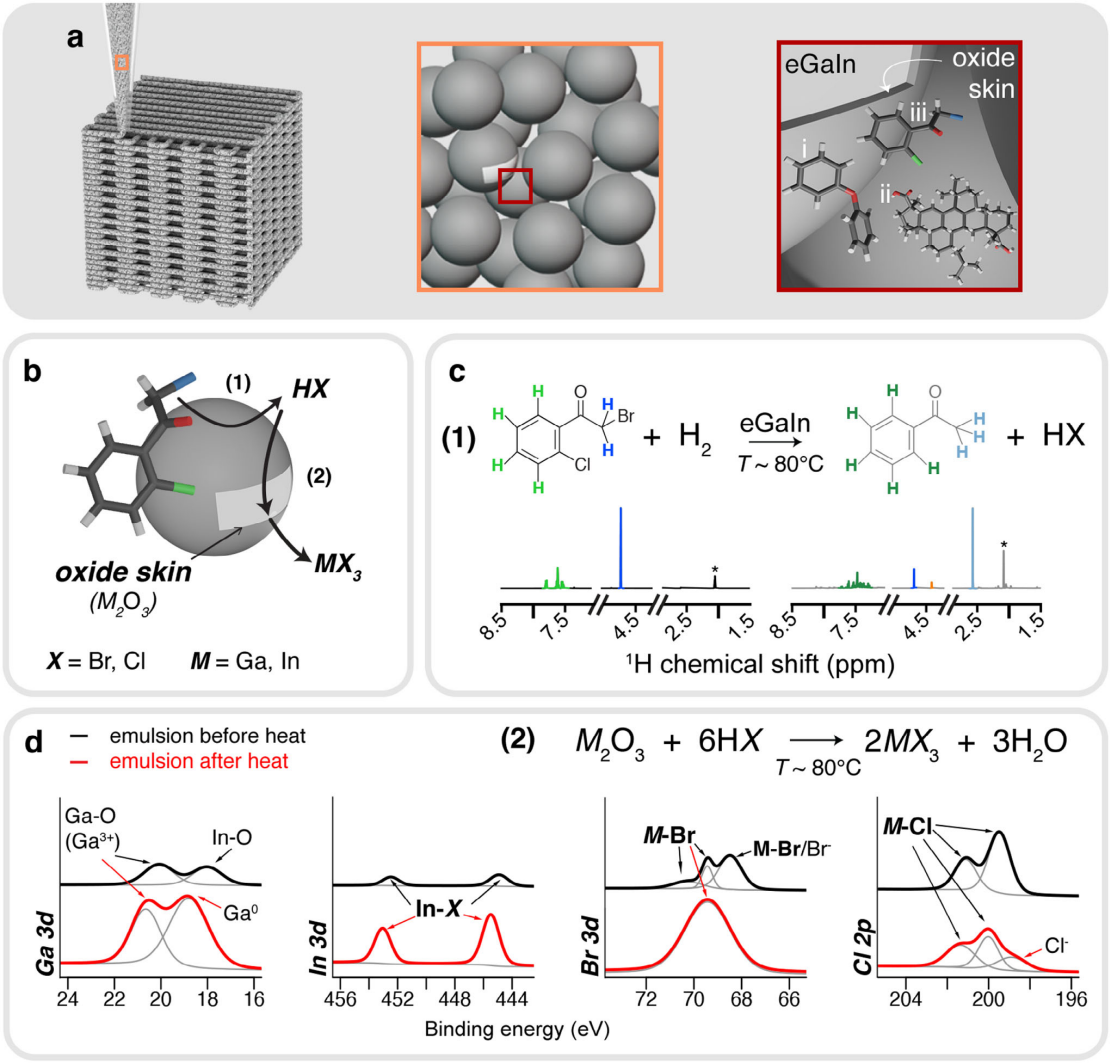
a) Schematic of 3D printable liquid metal emulsion ink. Left: 3D printable emulsion through direct ink writing. Middle (orange outline): the emulsion ink is comprised of dispersed liquid metal droplets. Right (dark red outline): In addition to eGaIn, (i) diphenyl ether (solvent), (ii) dimerized rosin (D140) and (iii) 2‐bromo‐2'‐chloroacetophenone (2B2c) exist in the ink. b) Schematic of chemical coalescence mechanism through 2B2c dehalogenation reaction (1), which releases a hydrohalide (HX), and is subsequently used for chemical etching reaction (2). (c) Dehalogenation reaction (1) of 2B2c occurs in the presence of eGaIn and heat (*T* = 80 °C) as shown through ^1^H NMR. Color of hydrogens in molecular structure correspond to NMR peak with the same color. The orange peak at 4.5 ppm in the heated LME results correspond to phenacyl bromide (Figure S4, Supporting Information), indicate dechlorination. The acetone solvent peak is denoted with a (*). d) Chemical etching of the metal oxide skin (reaction (2)) from the released hydrohalide is responsible for coalescence of eGaIn droplets, as evidenced through XPS spectra data collected on emulsions containing eGaIn, DE and 2B2c.

The technology enabling chemical coalescence within our LME is shown in **Figure** **1**a, where the dispersed phase of the emulsion consists of eGaIn droplets and the continuous phase contains i) diphenyl ether (DE), ii) dimerized gum rosin (D140) and iii) 2‐bromo‐2'‐chloroacetophenone (2B2c). DE is used as a solvent due to its low vapor pressure and high boiling point (258 °C),^[^ ^34^ ^]^ which helps prevent the ink from drying out during processing. DE is an aromatic non‐polar solvent^[^ ^34^ ^]^ that also dissolves the highly aromatic D140. D140 is a highly dimerized gum rosin, polymerized for stability against oxidation^[^ ^35^ ^]^ with a high molecular weight (*M*_w_ = 550 g mol^−1^) and a high acid value number (AVN) of 148.^[^ ^36^ ^]^ The AVN is a measure of the number of carboxylic acid groups^[^ ^37^ ^]^ which rosin is known for having.^[^ ^38^ ^]^ High AVN is needed for readily emulsifying eGaIn (Figure S1, Supporting Information), which occurs through carboxylic acid group attachment to the eGaIn oxide skin through coordination chemistry.^[^ ^39^, ^40^ ^]^ To enable chemical coalescence, we include the covalent halide compound, 2B2c, a type of aromatic ketone, which offers a source of halogens (bromine (Br) and chlorine (Cl)) for creating hydrohalides (HBr and HCl) to perform chemical etching. In 2B2c, Br is covalently bonded to the alpha carbon of the ketone group, while Cl is covalently bonded to the main aromatic ring. In contrast to dimethylammonium chloride (DAC), the stronger halide salt activator, which can also be used for this ink (Figure S2, and Note S1, Supporting Information), covalent halide activators offer a longer shelf life (i.e., they exhibit a significantly lower degree of coalescence over time under room temperature conditions).^[^ ^41^ ^]^ It is also both miscible and chemically inert with DE (Figure S3, Supporting Information).

#### 2.1.2 Catalytic Dehalogenation of the Chemical Activator

Figure 1b shows the two‐part scheme of dehalogenation (reaction 1) and chemical etching (reaction 2) that 2B2c undergoes for chemically coalescing eGaIn droplets. Dehalogenation involves the release of a halogen (represented as ‘*X*’ Br or Cl) from 2B2c. The released halogen combines with hydrogen gas (H_2_) generated between the reactions that gallium (Ga) and gallium suboxide (Ga_2_O) have with adventitious water in the air (Equations S2 and S3 in Note S2, Supporting Information) to then produce hydrohalide (“*HX*”) in reaction 1 scheme of Figure 1b, hydrobromic acid (HBr) or hydrochloric acid (HCl) (Equation S4, Supporting Information). Since metals have been found to dehalogenate covalent halide compounds similar to 2B2c,^[^ ^42^, ^43^, ^44^ ^]^ we hypothesize that eGaIn facilitates the dehalogenation of 2B2c, especially since liquid metals are known for catalyzing many chemical reactions.^[^ ^45^ ^]^ To validate this hypothesis, we bath sonicate a mixture of eGaIn and 2B2c in a closed glass vial to create an LME. Bath sonication is used in lieu of planetary shear mixing since shear mixing eGaIn and 2B2c results in viscoelastic mixtures that are unusable for NMR. DE is omitted to obtain a relatively clearer ^1^H‐NMR signal. After 45 min, the bath‐sonicated eGaIn/2B2c mixture is transferred to a hot plate and heated at 80 °C. After one hour, the organic layer is removed and characterized with ^1^H‐NMR. The results from this experiment are shown in Figure 1c (right) and compared to the ^1^H‐NMR spectrum for pure 2B2c (Figure 1c, left).

In comparison to ^1^H‐NMR spectra collected from pure 2B2c (Figure 1c, left), the NMR spectra of heated 2B2c alongside eGaIn (“2B2c_EGaIn, heated_”) reveals debromination evidenced by the appearance of a strong peak at 2.6 ppm, corresponding to acetyl protons (light blue), and the shrinking of the peak at 4.7 ppm, corresponding to the protons adjacent to bromine (blue). Additionally, dechlorination is indicated by the appearance of a peak at 4.5 ppm (orange). These hydrogens are noted in Figure S4‐ii (Supporting Information), which correspond to the alpha‐ketone protons after 2B2c undergoes dechlorination. The absence of Cl allows for more proton shielding and a downshifted signal. The appearance of new aryl hydrogen peaks at 7.1–8.2 ppm (green) indicates changes in the aromatic proton environment. For simplicity, reaction 1 displays the generation of two products. However, our NMR spectra suggests several other species are generated, as outlined in the more detailed reaction scheme shown in Figure S4 (Supporting Information). Notably, HBr is more readily generated than HCl during dehalogenation based on the intense acetyl group hydrogen peak (light blue) in the 2B2c_EGaIn, heated_ spectrum, which is consistent with the ease of debromination over dechlorination due to bond dissociation energies for C‐Br (63.1 kcal mol^−1^) < C‐Cl (91.5 kcal mol^−1^) in 2B2c.^[^ ^46^, ^47^ ^]^ Based on control experiments using the metals and metal oxides present in eGaIn to further elucidate the dehalogenation reaction mechanism (Figure S5, Supporting Information), we conclude that both gallium and indium in eGaIn catalyze the dehalogenation of 2B2c. Furthermore, we find that significant dehalogenation occurs in ambient conditions over the course of six days (Figure S6, Supporting Information), which further supports the the fact that eGaIn behaves as a catalyst in our LME.

#### 2.1.3 Chemical Etching of the Oxide

Chemical etching occurs through reaction scheme 2 shown in Figure 1b, where the released hydrohalide, *HX* (HBr or HCl) reacts with the oxide skin, *M*_2_O_3_ (Ga_2_O_3_ or In_2_O_3_) to generate metal halide salts, *MX*_3_ (GaBr_3_, GaCl_3_, InBr_3_, or InCl_3_) and water. During initial attempts to synthesize LMEs with eGaIn, DE, D140, and 2B2c, we find that 2B2c is capable of emulsifying eGaIn on its own, though the addition of DE is needed to attain a complete, homogeneous emulsion (Figure S7, Supporting Information). Thus, to monitor the effect that 2B2c has on chemical oxide etching within the emulsion before and after heat, we create an emulsion composed of ∼80 v/v% eGaIn, ∼17 v/v% DE, and ∼3 v/v% 2B2c for XPS characterization. The survey data for this experiment can be found in Figure S8 (Supporting Information), with main results shown in Figure 1d, where the fitted peaks for Ga 3d, In 3d, Br 3d, and Cl 2p orbitals for the emulsion before (black curves) and after (red curves) being heated at *T* = 80 °C for 1 h are shown. The *y*‐axis scale for each orbital in Figure 1d is adjusted for clarity and therefore is not the same across all orbitals. However, the spectrum for before and after heating within a given orbital display the actual intensity of each signal collected. For comparison, we also obtained the XPS for a drop of eGaIn only exposed to air (Figure S11, Supporting Information) to serve as a sample that has not been exposed to an activator or acidic environment.

In the black curves of Figure 1d, it appears that the 2B2c starts to coordinate with oxide surface during the emulsification process. This is shown through the presence of metal oxide and metal halide compounds in the XPS spectra. The presence of metal oxides is apparent in the Ga 3d orbital for the emulsion before heat, which displays two signals corresponding to Ga_2_O_3_ (∼20.1 eV, Ga^3 +^) and In_2_O_3_ (∼18.0 eV, In‐O), respectively.^[^ ^48^ ^]^ Conversely, the presence of metal halide complexes can be seen in the In 3d, Br 3d and Cl 2p orbitals. In the In 3d orbital, the non‐heated emulsion spectrum displays indium halide bonding (In‐*X*) through the In 3d_3/2_ and In 3d_5/2_ doublet peaks respectively centered at ∼452.5 eV and ∼444.9 eV, which correspond to InBr or InCl_2_.^[^ ^49^ ^]^ The formation of indium halide bonding is supported by the metal‐bromide (*M*‐Br) and metal‐chloride (*M*‐Cl) doublet peaks shown in the Br 3d and Cl 2p orbitals centered at Br 3d_3/2_ ∼70.3 eV and Br 3d_5/2_ ∼69.4 eV (Figure S9, Supporting Information) and Cl 2p_1/2_ ∼201.1 eV and Cl 2p_3/2_ ∼199.5 eV (Figure S10, Supporting Information),^[^ ^50^ ^]^ respectively. The Br 3d orbital for the non‐heated emulsion also displays an overlapping doublet peak at Br 3d_3/2_ ∼ 69.4 eV and Br 3d_5/2_ ∼68.5 eV, which is lower in binding energy and likely corresponds to InBr or adsorbed Br^−^.^[^ ^51^ ^]^ These additional Br 3d peaks align with our results that indicate debromination being the favored dehalogenation mechanism (Figure 1c), as multiple modes of metal‐Br are evident in the before heated Br 3d spectrum.

In the red curves of Figure 1d, we observe that heating provides even further evidence of chemical etching. After the emulsion is heated, it transforms into a mixture of bulk eGaIn and metal halide salts produced by the etching reaction. The formation of bulk eGaIn is evident in the Ga 3d orbital through the presence of Ga_2_O_3_ (∼20.7 eV, Ga^3 +^) and bare Ga metal (∼18.8 eV, Ga^0^), which also is present in its Ga 2p spectrum (Figure S8, Supporting Information). Although the heated emulsion Ga 3d peak values are higher than literature,^[^ ^48^ ^]^ we attribute the positive peak shift to suboxides of gallium and indium formed during the chemical etching process.^[^ ^52^ ^]^ The formation of metal halide salts can be seen in the In 3d, Br 3d and Cl 2p orbitals. The In 3d signal for the heated emulsion shows a signal for indium halide (In*X*_3_, either InCl_3_ or InBr_3_) through the In 3d_3/2_ and In 3d_5/2_ doublet peaks respectively centered at ∼453.0 and ∼445.5 eV.^[^ ^49^ ^]^ Formation of indium halide salt is further supported by the significant peak shifts from the unheated emulsion (+0.5 eV) and pure eGaIn (+1.2 eV), which represent the indium halide salt being transformed from intermediate indium‐halide complexes and In_2_O_3_, respectively (Figure S11, Supporting Information).^[^ ^53^ ^]^ In the Br 3d orbital for the heated emulsion, a peak at ∼69.4 eV is displayed, which is also present in the unheated emulsion Br 3d orbital. Since the In 3d orbital for the heated emulsion indicates In*X*_3_, we ascribe the strong Br 3d peak at ∼69.4 eV to indium‐bromide salts (InBr_3_) being generated from the hydrobromic acid etching reaction (Figure S9, Supporting Information). Lastly, the Cl 2p orbital for the heated emulsion suggests a conglomerate of metal‐chloride (*M*‐Cl) compounds (likely InCl_2_, InCl_3_, InCl)^[^ ^54^ ^]^) due to the subdued peak profile showing two prominent signals at ∼201.4 eV and ∼200.0 eV. Furthermore, adsorbed Cl^−^^[^ ^55^ ^]^ is present through the peak at ∼198.9 eV, suggesting that 2B2c dechlorination occurred after the emulsion was heated. We believe the results in Figure 1d constitute a snapshot of the dynamic reactions occurring within the emulsion. For example, while we expect to see a gallium‐halide signal in the Ga 2p orbital for the heated emulsion, the etching reaction produces water, which further generates Ga_2_O_3_ when reacting with Ga and Ga_2_O (see Equations S2 and S3 in Note S2, Supporting Information), but can also react with the metal halide salt products to generate additional acid for etching (see Equations S9–S12 in Note S2, Supporting Information). Furthermore, metal halide salts and/or metal‐hydroxide‐halogen complexes can be formed, as discussed in Equations S13–S16 in Note S2 (Supporting Information).

### 2.2 Liquid Metal Emulsion (LME) Formulations for Conductivity, Shape Retention, and Printability

To ensure compatibility of the LME with stencil patterning and DIW, eGaIn concentrations are fixed to ∼80 v/v% (i.e., well above ∼62%, the volume of random close packing of spheres)^[^ ^26^ ^]^ to create a dense emulsion with the viscoelastic behavior^[^ ^56^ ^]^ A concentration sweep is then performed of the DE/D140/2B2c continuous phase to define a region of attainable homogeneous emulsions. Figure S7 (Supporting Information) shows the ternary map of the continuous phase that explores the formulation space for achieving homogeneous emulsions. With the attainable homogeneous emulsion range identified from Figure S7 (Supporting Information), we then focus within this sub‐space (0.0–2.0 v/v% D140 rosin and 0.0–5.5 v/v%) to understand the effects of the concentrations of 2B2c and D140 on the post‐heat conductivity.

#### 2.2.1 Conductivity and Shape Retention

**Figure 2:**
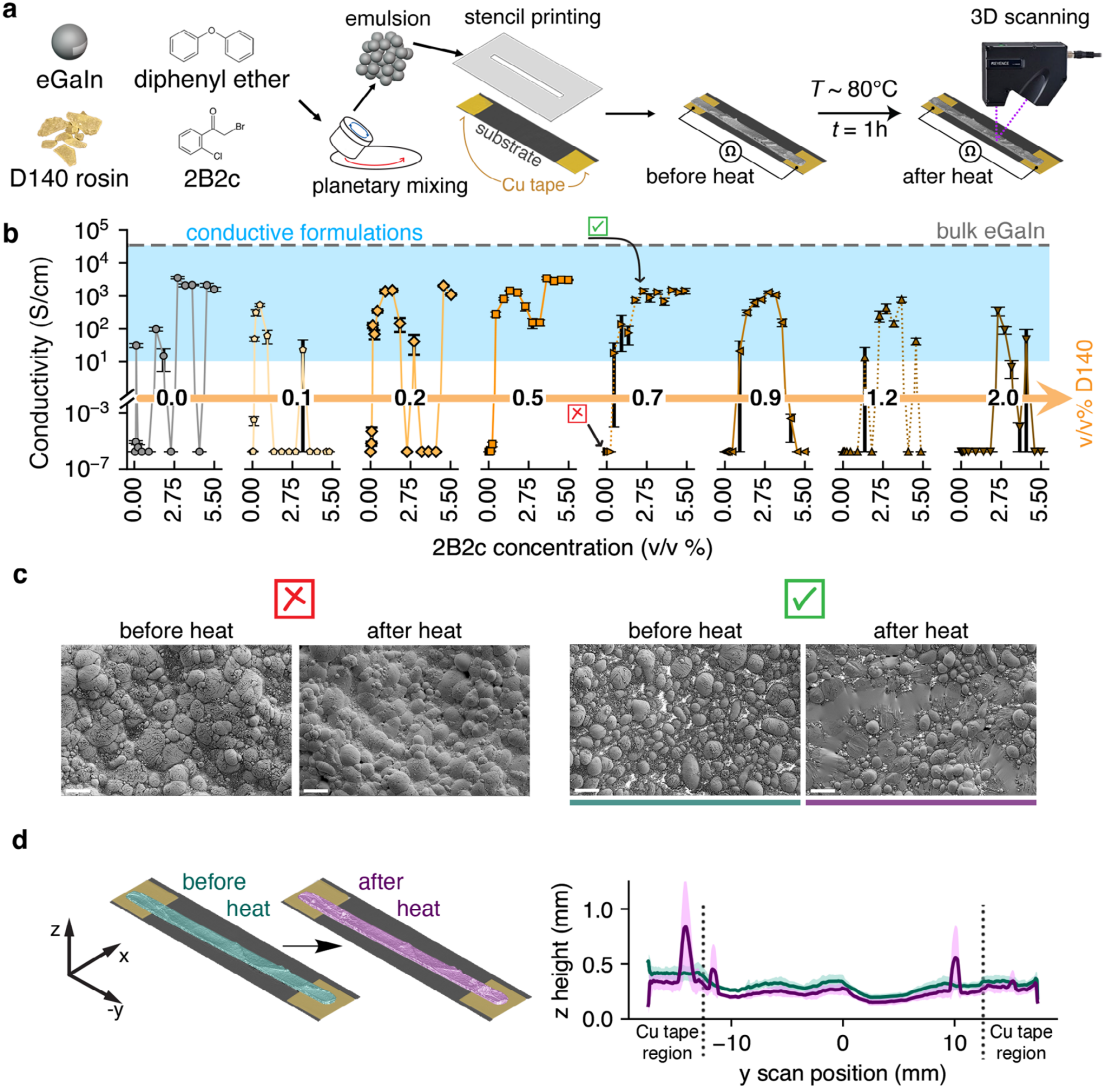
a) Process for testing formulated inks. Each formulation is shear mixed, stencil patterned on top of copper tapes and heated in an oven for one hour at *T* ∼ 80 °C. Resistances and 3D scans of each formulation before and after heating is collected to determine conductivity. b) Conductivity of formulated ∼80 v/v% eGaIn emulsions, with each subplot representing a 2B2c activator concentration sweep (0.0–5.5 v/v%) for a concentration of D140 rosin. c) Scanning electron microscope (SEM) images of 80 v/v% eGaIn emulsions before and after heat. The formulations for each emulsion is noted (b) by the symbol in the top left corner of the before heated SEM image. 2B2c activator is needed for eGaIn droplet coalescence and conductivity. Scale bar denotes 60 µm. d) The emulsion retains its shape after being heated: (left) Falsely colored 3D scans to accompany *z*‐height plot (right). (right) Average 2D height profile of liquid metal emulsion trace before and after heating.

The process for making a liquid metal emulsion sample is shown in the first half of **Figure** **2**a (details in Experimental Section). To make the emulsion, ground D140 is dissolved in DE. 2B2c is then added to the DE/D140 mixture, creating a DE/D140/2B2c premixture, which serves as the continuous phase of the emulsion. EGaIn is then added to the premixture and shear‐mixed to create dispersed liquid metal droplets, resulting in an emulsion. The concentrations of D140 and 2B2c are tuned by adjusting their premixture concentrations. Both D140 and 2B2c are emulsifiers, evidenced by our inability to create emulsions without them, and our ability to create LMEs with each ingredient individually as shown in Figures S1 (Supporting Information) and 1d. Increased emulsifier concentration and hence viscosity in the continuous phase has been shown to yield smaller mean droplet diameters.^[^ ^57^ ^]^ Thus, increased concentration of D140 and/or 2B2c increases premixture viscosity, which contribute to breakup of eGaIn into smaller droplets during emulsification. This can be seen in Figure S12 (Supporting Information) where the emulsion with the highest volume concentration of 2B2c (4.5 v/v%) has the smallest mean droplet diameter.

The second half of Figure 2a shows our process for monitoring a formulation's ability to coalesce to conductivity by measuring the resistance of a stencil‐patterned emulsion on top of two conductive copper tape pads before and after being heated at *T* = 80 °C for 1 h. 3D scans of heated emulsions are collected to accurately calculate post‐heat conductivity. Details of this process are outlined in the Experimental Section and in Figure S13 (Supporting Information). Before heating, the measured electrical resistance of all emulsion ink samples exceeded the maximum limit of the source meter, indicating a highly resistive material. Figure 2b shows the mean conductivity (*N* = 4) for each tested formulation after being subjected to heat. The effect of 2B2c concentration (*x*‐axis in each subplot) for a given concentration of D140 (labeled over the orange arrow) is displayed. After mild heat treatment, only the LME formulations highlighted by the light blue section spanning each subplot were considered electrically conductive (σ ⩾ 10 S cm^−1^). Based on the stoichiometry of 2B2c dehalogenation and Ga_2_O_3_ oxide etching reactions and assuming perfectly spherical eGaIn droplets (diameter = 26.51 µm) with a 3 nm oxide skin,^[^ ^58^ ^]^ we estimate that 0.82 v/v% 2B2c is required to etch all the oxides in an 80 v/v% liquid metal emulsion after heat, which also assumes that 2B2c undergoes 100% dehalogenation (Note S8, Supporting Information). However, experimentally, we find that our emulsions require higher concentrations of activator (∼2.5–5.0 v/v% 2B2c within each D140 concentration set) to attain high electrical conductivity. If instead we assume 100% debromination, we estimate that 1.63 v/v% 2B2c would be required to etch all oxides for an 80 v/v% liquid metal emulsion. Since we know from XPS and NMR that debromination is the favored dehalogenation reaction and is not 100% (Figure 1c,d), this explains the higher 2B2c concentration requirement for our system to attain higher degrees of chemical etching and thus higher electrical conductivity. The highest mean conductivity obtained was 3.54 × 10^3^ S cm^−1^ for an emulsion that utilized 0.0 v/v% D140 and ∼3 v/v% 2B2c. Comparable conductivities were found for emulsions formulated with 0.5 v/v% D140 and 4–5 v/v% 2B2c (∼3 × 10^3^ S cm^−1^). Based on Figure 2b, it can be concluded that 2B2c is necessary for achieving conductivity, evidenced by non‐conductive formulations with 0.0 v/v% 2B2c activator for each concentration of D140. Furthermore, the results in Figure 2b show that a D140 concentration between 0.5–0.7 v/v% offers the potential to yield conductive emulsions at 2B2c concentrations higher than 5.5 v/v%, while lower or higher concentrations of D140 (0.0–0.2 and 0.9–2.0, respectively) yield inconsistent conductive behavior in the tested concentration range of 2B2c. Since D140 and 2B2c contribute to emulsification, we hypothesize that at low concentrations of D140, the two molecules compete for eGaIn oxide surface attachment,^[^ ^59^ ^]^ resulting in highly resistive emulsions through a depletion of available 2B2c to enable coalescence. At higher D140 concentrations, the relatively large D140 molecule might act as an effective surfactant in stabilizing eGaIn droplets^[^ ^60^ ^]^ and preventing droplets from coalescing into a fully connected conductive network.

Chemical coalescence enabled by 2B2c is further supported by the scanning electron microscope image shown in Figure 2c, which shows emulsions formulated at ∼0.7 v/v% D140 rosin: one with no 2B2c activator (left two images) and one with ∼2.5 v/v% 2B2c (right two images), with their corresponding conductivity results indicated in Figure 2b. Before heating, both formulations show dispersed eGaIn droplets. After heating, the formulation containing 2B2c shows clear signs of coalescence among eGaIn droplets shown by the smooth connected surface spanning the droplets. We note that, within this emulsion, there are still uncoalesced eGaIn droplets, which contribute to emulsions being unable to reach bulk eGaIn conductivity. It is possible to increase chemical coalescence and thus electrical conductivity (2.4 × 10^4^ S cm^−1^) to that of bulk eGaIn by using stronger chemical activators such as dimethylammonium chloride (DAC), a halide salt (Figure S2). Halide salt‐based LMEs achieve the highest conductivity compared to LMEs utilizing other activation modes, outperforming most liquid metal composites a well as PDMS encased bulk liquid metal conductors^[^ ^61^ ^]^ (Figure S15 and Table S5, Supporting Information). Although the electrical conductivity of our chemically coalescing LMEs is not as high as one of the SEBS‐encased bulk liquid metal conductors^[^ ^62^ ^]^ and the nickel‐eGaIn composite,^[^ ^63^ ^]^ our LMEs do possess the benefit of being compatible with a variety of patterning techniques, such as stencil patterning and 3D printing. Heat activating (*T* ≈ 80 °C) free‐standing geometries of patterned DAC‐based emulsions, however, results in disconnected coalesced eGaIn droplets that are not conductive (Figure S2d, Supporting Information). While covalent halide LMEs possess lower conductivity, they possess more shape retention after being heated (Figure 2d), which has more utility for achieving 3D forms with preserved structural integrity throughout the coalescence process. The presence of the uncoalesced eGaIn droplets and potentially chemical byproducts from 2B2c dehalogenation and oxide etching reactions in our covalent halide activator‐based emulsion help retain its shape after undergoing chemical activation. The mean height of the patterned trace (“*z* height”) (across the *x*‐position) versus its position along its length (“*y* scan position”) is shown on the right of Figure 2d. It can be seen that the general shape of the average *z* height stays the same before and after heat activation between the copper tape, having a volume shrinkage of only ∼12.9% (Figure S16, Supporting Information). In contrast to the volume concentration of non‐eGaIn emulsion ingredients that are liquid and likely to evaporate or react during heat activation (diphenyl ether (∼16.8 v/v%) and 2B2c (∼2.5 v/v%)), the calculated volume shrinkage from 3D scan data is ∼6.4 % lower. This discrepancy suggests that compressed eGaIn droplets, as well as solid byproducts such as metal halide salts from oxide etching reactions discussed in SI Note 2, resist further shrinkage, contributing to its shape retention. Notably, the height changes drastically in the region of the copper tapes, which is due to eGaIn preferentially wetting the copper.^[^ ^64^ ^]^

#### 2.2.2 Printability

**Figure 3:**
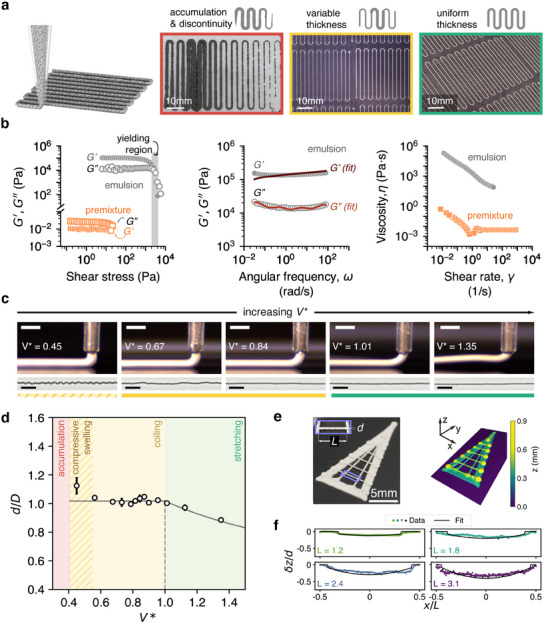
a) Print outcomes of ∼80 v/v% eGaIn liquid metal emulsion inks: Accumulation and discontinuity (red), variable thickness (yellow), and uniform thickness (green). b) Rheology of emulsion ink (0.7 v/v% D140, 4.5 v/v% 2B2c) mixed for 67.5 min at 15.2% relative humidity (RH) showing its printability. Left: viscoelastic behavior of the emulsion ink. The inclusion of eGaIn at high concentration in the emulsion is needed for viscoelasticity. Middle: The relaxation spectrum, which shows the emulsion is predominantly elastic. Right: Flow curve showing shear thinning characteristics of the emulsion. c) Extrusion behavior of emulsion ink (0.7 v/v% D140, 4.5 v/v% 2B2c, mixed for 32.5 min at 21.7% RH) at varying print velocities (V*). Top row: microscope view of print nozzle (scale bars indicate 0.5 mm). Bottom row: resulting filaments at select V*s, printed at 0.5 mm nozzle height. V* = *V*/*C*, where *V* is the print velocity and *C* is the determined ink extrusion velocity. Scale bars indicate 2 mm. d) Nondimensionalized printed filament width (*d*/*D*, where *D* is the nozzle diameter) vs. V* shows how the printed filament varies with V*. e) Spannability of emulsion ink (0.7 v/v% D140, 4.5 v/v% 2B2c, mixed for 60 min at 16.1% RH). Left: Orthographic view of a triangle containing different spanning features, with inset defining variables used in spanning calculations (*d* is filament width, *L* is span length). Right: Height map of printed triangle generated from 3D scan on left. f) *z*‐deflection (normalized by filament diameter, *d*) versus *x*‐coordinate (normalized by length, *L*) for spanned lines. The length of each spanned line is denoted in lower left of each subplot. Colored data points represent mean *z* height data along the length of the trace from 3D point clouds; solid black lines represent the model fit of the viscoelastic bending and stretching equation.

**Figure** **3**a shows the range of qualitative printability outcomes – not printable (red), mildly printable (yellow), and printable (green) of formulated inks. LMEs are mixed longer (an additional 10 min for the emulsions shown in red and yellow; and an additional 12.5 min for the emulsion shown in green) than the stated mixing times listed in Table S3 (Supporting Information) to increase its viscoelastic behavior through smaller droplet size creation,^[^ ^17^, ^65^ ^]^ then tested for printability. Printability is then assessed as described in Experimental Section and Figure S17 (Supporting Information). Printed serpentine patterns for a particular formulation are visually inspected and qualitatively categorized based on extrusion consistency at a pressure specifically determined for each formulation (Experimental Section), and a print velocity of 70 mm s^−1^, which is used across all ink formulations. An ink that displays consistent extrusion throughtout all serpentine prints is deemed printable, while any extrusion inconsistencies rules out a formulation as being unprintable. Although D140/DE or 2B2c can emulsify eGaIn on their own, synthesizing a printable emulsion based only on eGaIn/DE/D140 or eGaIn/DE/2B2c is not feasible as this results in (1) highly inconsistent extrusion marked by accumulation/discontinuity (red outline in Figure 3a, ink composition: ∼80.3 v/v% eGaIn, ∼19.5 v/v% DE and ∼0.2 v/v% D140) or (2) moderately inconsistent extrusion marked by variable filament thickness (yellow outline in Figure 3a, ink composition: ∼80.0 v/v% eGaIn, ∼14.5 v/v% DE, and ∼5.5 v/v% 2B2c). Thus, a combination of D140 rosin and 2B2c is needed for producing a printable emulsion. The formulation that displays the highest consistency during our printability experiments as well as the highest reported conductivity (green outline inset shown in Figure 3a) is selected as our optimum ink. The composition of this ink is ∼80 v/v% eGaIn, ∼0.7 v/v% D140 and ∼4.5 v/v% 2B2c, with a conductivity of 1.50 × 10^3^ S cm^−1^.

#### 2.2.3 Characterization of Optimum Liquid Metal Emulsion Ink

Two main observations during ink synthesis confirm the chemical complexity of our emulsion ink system. First, we find that the relative humidity in the lab decreased the total mixing time required to obtain a printable, viscoelastic consistency with our emulsion (Figures S18 and S19, Supporting Information). This finding is consistent with the role of adventitious water in the environment contributing to the hydrogen supply for producing the HCl and HBr needed for etching. Second, we find that further mixing the ink to increase printability reduces its conductivity by two orders of magnitude (1.23 × 10^1^ S cm^−1^) post‐heat activation (*T* = 80 °C, 1 h) (Figure S20 and Table S7, Supporting Information). Since longer mixing times create smaller droplets,^[^ ^17^ ^]^ the lower observed conductivity is likely due to the increased presence of oxides due to smaller droplet sizes, which also contribute to an increased stiffness (*G*′) (Figure S18, Supporting Information). For undermixed emulsions of the optimal formulation we find that longer mixing time achieves smaller droplets (Figure S18, Supporting Information), which is consistent with results from other high shear emulsification processes.^[^ ^17^, ^65^ ^]^ We also find that the decrease in droplet size results in an increase of *G*′ (Figure S18 and Table S6, Supporting Information). Notably, we find a difference in drop morphology between the optimally mixed emulsions and the undermixed samples (Figure S18b, Supporting Information), where the droplets transition from separate and discrete to seemingly more aggregated and interconnected with apparent cracks on their surfaces with freshly exposed eGaIn. We identify this morphology as a state just before creaming, which also gives rise to a gray macroscale optical appearance (Figure S18a, Supporting Information) and a corresponding significant increase (approximately one order of magnitude) in *G*′ (Figure S18c and Table S6, Supporting Information). Evidently, this morphology improves printability, since undermixed inks also display print inconsistencies such as variable thickness, discontinuities, and ruptures (Figure S18d, Supporting Information). Also, since our emulsion is catalytic, heating the emulsion at *T* = 80 °C for a longer time can improve conductivity. Furthermore, using higher activation temperatures (120−160 °C) with the optimum ink helps to achieve higher post heat conductivity (1.07 × 10^3^ S cm^−1^ at 120 °C), lower contact resistance, and a higher degree of coalescence by accelerating the chemical reaction with the emulsion (Figures S20 and S21, Supporting Information).

Rheological characterization in Figure 3b (method details in Experimental Section) of the selected formulation shows that the emulsion (circle markers in all Figure 3b subplots) is a stiff, viscoelastic, yield stress material with shear thinning behavior. The oscillation amplitude sweep results in Figure 3b (left) show that the emulsion has high stiffness (storage modulus, *G*′ ∼1.0 × 10^5^ Pa) in the linear viscoelastic region (plateau where *G*′ > *G*″ at low shear stresses) and yields when a stress of 2308.02 ± 336.38 Pa is applied (light gray region, see Figure S22, Supporting Information, for yield stress calculations). This value is 1–2 orders of magnitude higher than liquid metal emulsions with lower concentrations of eGaIn (∼60–76%),^[^ ^20^, ^21^ ^]^ which is expected since yield stress directly scales with dispersed phase concentration.^[^ ^66^ ^]^ Furthermore, compared to water‐based liquid metal emulsions with similar eGaIn content (∼80–85%)^[^ ^22^ ^]^ to our optimal formulation, our calculated yield stress is roughly the same order of magnitude though ∼2 times higher. We attribute the differences in yield stress to differences in the continuous phase between these emulsions: in the presence of water, eGaIn's oxide will weaken,^[^ ^67^ ^]^ which potentially contributes to weaker interdroplet interfaces thus resulting in lower yield stress. Comparing the emulsion oscillation data with that of the continuous phase of the emulsion, which displays dominantly viscous behavior (*G*″ > *G*′), it can be seen that the incorporation of eGaIn imparts the emulsion with the required viscoelasticity for DIW 3D printing. The elasticity is due to the high concentration (∼80 v/v%) of dispersed and compressed eGaIn droplets within the emulsion, which stores energy at the droplet interfaces.^[^ ^56^ ^]^ The associated Lissajous figures for this oscillation data shown in Figure S23 (Supporting Information) suggests that the emulsion undergoes an interesting fluid transition from being highly elastic to perfectly viscous after a yield stress is applied.

The high elasticity is further confirmed by Figure 3b (middle), which shows oscillation frequency sweep data at a strain amplitude γ_0_ = 1%, corresponding to the linear viscoelastic region in our amplitude sweep experiments. Throughout the frequency range tested, the *G*′ plateaus and remains higher than *G*″, indicating the emulsion's dominantly elastic behavior, while *G*″ exhibits a shallow minimum at ω ∼ 1 rad s^−1^. Mason et al.^[^ ^56^ ^]^ attribute this behavior to slow relaxation processes occurring within the emulsion due to droplet rearrangement or viscous relaxation of the continuous phase fluid. The behavior in this spectrum is likened to that of soft glassy materials, as Datta et al.^[^ ^68^ ^]^ described for highly concentrated attractive emulsions. This is further validated by our fitting of the data to the discrete form of the Maxwell relaxation modulus equation (Figure S24, Supporting Information), which yields relaxation modulus values that stay within the same order of magnitude, as opposed to spanning different orders of magnitudes, as typically found in material systems exhibiting a more pronounced viscous behavior.^[^ ^68^, ^69^ ^]^

The flow data (Figure 3b, right) shows that the emulsion is shear thinning with decreasing viscosity values upon increasing shear rate. Compared to the premixture, its viscosity is five orders of magnitude larger, which is consistent with the elastic phenomena imparted by compressed droplets observed in the oscillation data in Figure 3b, left. The continuous phase (DE/D140/2B2c) displays shear thinning at low shear rates followed by a viscosity plateau (Newtonian fluid behavior) at increasing shear rate, indicating that the premixture itself is a structured fluid.^[^ ^70^ ^]^ The corresponding stress versus shear rate graphs for the emulsion and premixture (Figure S25a,b, Supporting Information) show stress plateaus indicative of shear banding and is similar to flow behavior of entangled polymeric‐like micellar solutions that break and reform.^[^ ^71^ ^]^. Since the flow curves of DE and DE/D140 mixtures display Newtonian behavior (Figure S25c and d, Supporting Information), this suggests that inclusion of the 2B2c activator imparts shear thinning properties to the DE/D140 mixture, which likely imparts consistent extrusion behavior for inks with 2B2c in contrast to the inconsistent extrusion for inks without 2B2c. Extending the comparison of micellar breaking and reforming to our ink, we conclude that the emulsion flows through a process of rapid interdroplet breaking^[^ ^68^ ^]^ of the weakest bonds between compressed eGaIn droplets (described as “compressed springs” by Mason et al.^[^ ^56^ ^]^), followed by their rapid reformation within the emulsion.

Figure 3c and d shows how interdroplet breaking and reforming within the emulsion manifests itself as a function of print speed (*V*) at a nozzle‐to‐substrate height (*H*) of 0.5 mm. This print height is chosen for being below the characteristic height (*H*_vg_ = 10 mm) where gravitational and inertial effects could be neglected (Note S11, Supporting Information). We define the dimensionless print speed V* as the print speed *V* divided by the extrusion velocity *C*. At V* = 0.45, it can be seen that the extruded emulsion laterally expands upon substrate landing and produces a thicker filament, evidenced by the high value of the ratio of the printed filament diameter *d* to the nozzle diameter *D* in Figure 3d. We attribute this deviation from the constant *d*/*D* behavior in the coiling region to axial compression due to the velocity of ink extrusion being significantly larger than print nozzle speed and to deformations within elongational flow^[^ ^72^, ^73^ ^]^ as the filament experiences buckling (coil). As seen in Figure 3d and Video S2 (Supporting Information), at V* = 0.56, the filament is slightly larger than the width of the nozzle diameter due to die swelling, indicating a transition from combined axially compression and buckling to only buckling. Thus, in Figure 3d, we define a compressive swelling zone (0.41 < V* < 0.56) that illustrates this phenomena. As V* increases, the slenderness of the extruded emulsion increases, resulting in pure buckling, which manifests as irregular meandering of the filament^[^ ^74^ ^]^ in V* ∈ (0.67, 0.84). Because the eGaIn droplets are highly compressed inside the nozzle, this lack of periodicity can be attributed to differences in individual droplet velocities and accelerations previously reported for highly concentrated emulsions under flow.^[^ ^75^ ^]^ As V* approaches 1.00, the effect of coiling disappears resulting in a straight filament. In Figure 3d, we construct a printing phase diagram for this ink based on the calculated filament thickness from extrusion experiment in Figure 3c. To estimate ink extrusion velocity *C* through Yuk and Zhao's 3D printing phase diagram, we performed a nonlinear least squares fit of the piece‐wise equation to filament data printed at V* > 0.45 (see Experimental Section), which estimates the extrusion velocity of the ink at *C* = 88.91 ± 0.10 mm s^−1^ and die swelling ratio, α = 1.02 ± 0.0002. Calculation of *C* allows us to highlight the filament accumulation, coiling, and stretching print regions^[^ ^69^ ^]^ for our optimum emulsion ink.

Figure 3e shows how our emulsion ink has the ability to span gaps. In Figure 3f, we fit the equilibrium viscoelastic bending and stretching equations developed by Zhu and Smay for spanning catenary‐shaped filaments^[^ ^76^ ^]^ to the 2D mean *z*‐height data for all printed lines. The piecewise deflection outlined in Equation (3) relies on physical and elastic properties of the ink (see Experimental Section, Tables S9, and S10 for model parameter values, Supporting Information). Since the bending boundary layer, δ, is initially unknown and is dependent on *G*′, model‐fitting entails providing initial guesses of *G*′ = 0.80–1.05 × 10^5^ Pa, values of *G*′ collected in oscillation‐based rheology experiments. Each initial *G*′ guess also provides a guess to δ. For each initial *G*′ guess, the model estimates *G*′ ≈1.00 ± 0.03 × 10^5^ Pa, which closely agrees with the *G*′ collected in the oscillation amplitude experiment at low shear stress (5–10 Pa) in Figure 3b ($G_{\text{average}}^{\prime }=1.03\times 10^{5}$ Pa) as well as data on colloidal gels.^[^ ^76^, ^77^ ^]^ We use the result *G*′ = 1.00 ± 0.03 × 10^5^ Pa to approximate δ for each spanned line to display model results (δ = 237, 208, 189, and 175 µm for lines with *L* = 1.2, 1.8, 2.4, and 3.1 mm, respectively) with a linear fit (slope ∼|1.33| mm mm^−1^) connecting the bending and stretching regions for each line. Figure 3f shows how the data follows the predicted trend for decreasing δ's as well as increasing deflection and stretching as the spanned length *L* increases.

### 2.3 Demonstration of a Hybrid 3D Printed LME‐Based Device with Integrated Batteries

**Figure 4:**
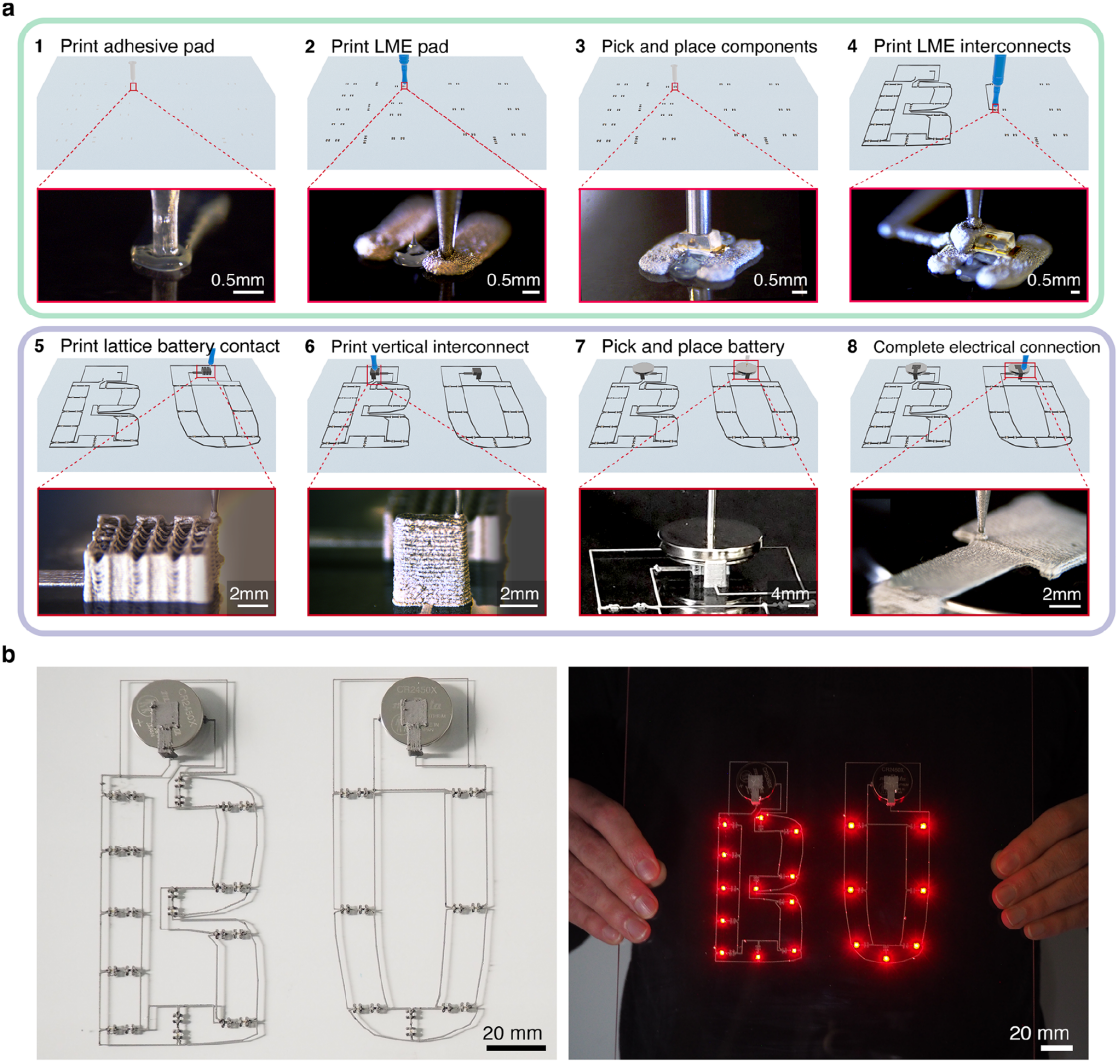
a) Automated process of complex device fabrication using liquid metal emulsion ink, using multimaterial printing and automated pick and place for surface mount device components. b) Photographs of assembled LED device (left) before being heated at *T* ∼ 80 °C, (right) after being heated. After heat, the functioning device can be held up vertically due to the adhesive properties of the coalesced emulsion.

To demonstrate the utility of chemically coalescing emulsions in simplifying liquid metal‐based electronics fabrication, we create a multi‐component electronic device through hybrid 3D printing.^[^ ^78^ ^]^ Our chemically coalescing ink not only facilitates programmable device fabrication by acting as electrical interconnects for light emitting diode (LED) and resistor arrays, but also enables device functionality through mild activation temperatures that are compatible with commercial batteries. Furthermore, the chemistry in our ink also acts as a solder between the coalesced eGaIn and the electrical pads on surface mount device (SMD) components. The use of a chemically coalescing emulsion ink consists of three main steps: 1) circuit design, 2) hybrid 3D printing, and 3) mild heat post‐processing. First, the circuit is designed with custom Python codes to define print paths and component placement. Electrical checks for circuit designs are validated using MultiSim Live. The circuit diagram schematic of the LED array used in this work is shown in Figure S27 (Supporting Information). Next, the device is fabricated through multimaterial and hybrid 3D printing (details outlined in Experimental Section). The workflow for this step can be seen in **Figure** **4**a, which consists of surface mount device component interfacing (green outline in Figure 4a) and inline assembly of battery power and electrical interconnections (purple outline in Figure 4a). Lastly, the printed and assembled LED array device (Figure 4b, left) can be made functional through mild heat (*T* = 80 °C), which can be seen by the entirely lit LED array (Figure 4b, right). For timelapse of device activation in the oven for an analagous single LED device, see Video S4 (Supporting Information). For a timelapse of the full fabrication process for the BU LED array, see Video S5 (Supporting Information).

#### 2.3.1 Design Requirements for Optimal Ink Activation

To make optimal connections to electrical components, we find that paths for printing electrical interconnects must be designed so that enough liquid metal emulsion ink interfaces with SMD component electrical pins. Both the LED and resistor components have pins that are exposed to the LME at the top and bottom. In a typical printed circuit board (PCB) fabrication process, SMD components are placed on top of solder paste so the bottom of the SMD pins primarily connect to the copper routing underneath.^[^ ^79^ ^]^ Initially, we fabricate devices printing the emulsion ink solely on top of SMD electrical pins, which result in electrical connections being inconsistently formed, as shown by the variable intensity in light of LEDs in Figure S28 (Supporting Information). In subsequent devices, after printing a SMD component adhesive (Figure 4a, step 1), to maximize the interfacial area between the SMD electrical pins and the emulsion ink, we print a rectangular pad (Figure 4a, step 2) with the emulsion ink to interface with the bottom part of the SMD electrical pins as well as printed electrical traces. This is followed by pick and place of the components (Figure 4a, step 3), and then printing of the emulsion ink on top of each SMD pin (Figure 4a, step 4). This is similar to the work by Ozutemiz et al in finding that better electrical connections are formed when eGaIn surrounds the entirety (top, bottom and side) of the SMD pin after HCl treatment, versus a connection formed when pure eGaIn interfaces primarily with the bottom of an SMD pin.^[^ ^80^ ^]^

In addition to having sufficient material to interface with pins, we find that our emulsion ink requires exposure to air for chemical coalescence. To explore the utility of our ink to electrically interface with a coin cell battery, we print single LED devices with flat (∼200 µm thick) square pads on the top and bottom electrode connected by a vertical interconnect (Figure S29, Supporting Information). When subjecting the printed and assembled battery‐integrated single LED device to mild heat post processing at *T* = 80 °C overnight, it appears that LME printed geometries of the circuit (electrical interconnects, top current collector, vertical interconnect) are conductive and show signs of coalesced eGaIn by emerging through the structure (Figure S29a, Supporting Information). However, we observe incomplete coalescence of the square pad located below the battery, evidenced by the inhomogeneous color (Figure S29b, Supporting Information), which we confirm by performing electrical conductivity checks using an external coin cell battery with leads throughout the printed emulsion (Figure S29c–e, Supporting Information). Since the only part of the device containing non‐conductive liquid metal emulsion is underneath the battery, we hypothesize that an increased emulsion/air interface is needed for the chemical coalescing reaction to occur. Thus, we implement a simple cubic lattice design for the bottom current collector to elevate the battery to not only increase the emulsion/air interface but also to increase the air flow to the emulsion/battery bottom (Figure S29f, Supporting Information). As a result, we observe more coalescence evidenced by emerging eGaIn droplets from the newly designed printed geometry (Figure S29g, Supporting Information) and subsequently increased conductivity as LED devices utilizing this elevated bottom electrode design are all functional after being heated.

#### 2.3.2 Shape Retention, Ink Adhesion, and Stability Over Time

As mentioned previously, our emulsion retains its patterned shape, which can also be seen in the printed liquid metal emulsion patterns in the before and after heat images of the hybrid 3D printed LED array (Figure 4b). This can be seen on a bigger scale in a printed “BU” logo in Figure S30a (Supporting Information) where light gray bumpy textured layer is holding emerging liquid metal droplets within the print after being heat activated at 80 °C. We also find that an opaque layer forms underneath the print at the LME‐substrate interface with a smoother surface and occasional voids, as seen in Figure S30b, which is also observed in the aforementioned battery redesign study (Figure S29, Supporting Information). This structured layer is also observed in the microscope images in Figure S21 (Supporting Information), which exhibits an increased spatial occurrence of liquid metal striations with increased activation temperatures. Removing the activated BU logo print from the glass substrate requires scraping with razor blade, indicating a qualitatively a large amount of shear, thus a large degree of adhesion between the glass substrate and the activated emulsion. Notably, it is difficult to scrape the opaque layer left on the glass substrate (Figure S30c, Supporting Information). The relative difficulty in attempting to scrape the printed sample off the glass substrate may be a combination of the strong adhesion to glass by the oxide skin^[^ ^81^ ^]^ and D140 carboxylic acid groups.^[^ ^82^ ^]^ The generation of strong adhesion during coalescence can be explained by Doudrick et al^[^ ^83^ ^]^ through the wetting phenomena of a volume expanding liquid metal droplet whose oxide skin fractures, exposes bare liquid metal and forms new oxide on the substrate. Furthermore, carboxylic acid groups are known to coordinate with the oxide skin and glass,^[^ ^82^ ^]^ which likely contributes to this adhesive quality. This strong adhesion is also seen in the right of Figure 4b, where the hybrid 3D printed LED array can be held vertically without the device components falling off all the while remaining functional. Although the LED and resistor components are adhered to the substrate with a silicone adhesive, the relatively heavier (∼6.2 g) coin cell battery is stably bound to the substrate solely through the bottom LME printed current collector. We estimate the adhesive stress for the coalesced emulsion ink is at least 2.2 MPa (see Note S13, Supporting Information). This is similar in magnitude to measured lap shear strengths of state‐of‐the‐art electrically conductive adhesives (∼6–18 MPa).^[^ ^84^ ^]^ In contrast to findings on metal embrittlement by GaLMAs,^[^ ^6^ ^]^ metal degradation is not observed on the battery current collector.When our coalesced LME is integrated with PDMS and copper, we also find that it preferentially adheres to copper (Figure S31, Supporting Information), which suggests adhesion is substrate‐dependent. Lastly, we find that not only is the shape retention and adhesion is generally stable but the electrical conductivity of the printed and heat activated ink also increases when stored at ambient over the course of ten months (Note S14 and Figure S32, Supporting Information). This increase in conductivity is expected and consistent with our previous findings that the chemical coalescing reaction occurrs slowly at ambient (SI Note 5), which may be attributed to the metal halide salt hydrolysis reactions that generate acids and perform more oxide etching (Note S2, Supporting Information). Environmental conditions such as higher temperature and/or humidity may also contribute to this increase in conductivity by increasing droplet coalescence (Note S2, Figure S20, Supporting Information), slightly decreasing shape retention due to the liquid metal emergence from the sample (Figure S21, Supporting Information). Decreased shape retention, however, can be addressed by packaging the LME after it is printed and activated (Figure S34, Supporting Information). Figure S34a (Supporting Information) shows this assembly process for incorporating our LME into PDMS to make a dogbone device, which was electromechanically characterized (200% strain/1000 cycles, results shown in Figure S34b, Supporting Information). The electrical conductivity of the packaged activated LME is increased with additional mechanical forces (e.g., peeling it off the substrate, stretching during testing) which is expected and consistent with mechanical activation of packaged liquid metal inclusions.^[^ ^21^, ^27^ ^]^ Moreover, we find that these electromechanically tested devices are stable (Figure S35a, Supporting Information) and can withstand prolonged high temperatures (125 °C/2 days), given the absence of any packaging weaknesses (Figure S35b, Supporting Information).

## 3 Limitations and Future Work

While the covalent halide‐based LME emulsion in this work offers a material solution for simplifying GaLMA device fabrication requiring soft conductors, there are many opportunities to improve the post‐heat conductivity while retaining its patterned shape. For one, chemical oxide etching could be further and controlled by conserving 2B2c for the heat activation step. This can be done by dispersing eGaIn into a DE/D140 premixture through tip sonication or shear mixing to first make an LME largely stabilized by D140. 2B2c could be added to the eGaIn/DE/D140 emulsion to ensure it participates in oxide etching and not emulsification. Secondly, the generation of H_2_, which is needed for hydrohalide (HX) production, could be improved by including H_2_‐producing ingredients into the LME. This could include a systematic formulation study with controlled amounts of water to increase H_2_ yield (Equations S2 and S3, Supporting Information). Furthermore, inclusion of protic solvents with maximal hydrogen donating ability^[^ ^85^ ^]^ can help supply more hydrogens in the system, but this also must be investigated methodically as protic solvents assist in the hydrodehalogenation of covalent halide compounds,^[^ ^86^ ^]^which can prematurely start the chemical etching reaction and thus yield an unstable emulsion. To increase the shelf‐life of LMEs, alternate covalent halide activators with higher carbon‐halogen bond dissociation energies (BDEs) or increased levels of steric hindrance can be used (Table S13, Supporting Information), which would delay dehalogenation and thus oxide etching. Since our work shows that covalent halide activators promote shape retention for chemically coalescing liquid metal emulsions, studies for increasing electrical conductivity through higher oxide removal could be conducted by considering covalent halide compounds with lower BDEs (Table S13, Supporting Information), or even combining covalent halide compounds with halide salt activators, which we have shown to be more effective at oxide etching. A combination activator study could involve combining the right stoichiometric ratio of activator types for 100% oxide removal while considering BDE. One idea for achieving an LME is to create an LME/polymer composite through the use of silane‐based monomers. Silanization is a way to permanently bond to liquid metal surfaces,^[^ ^87^ ^]^ which can ensure droplet connectivity as the oxide is etched. Tetramethyldisiloxane, for one, can be used as an ingredient in co‐polymerization,^[^ ^88^ ^]^ and has also been found to release H_2_ in the presence of covalent halogen compounds and a palladium catalyst,^[^ ^89^ ^]^ which can assist with further etching. Beyond these additional oxide removal studies, more studies can be done to further elucidate the adhesion mechanisms between the activated emulsions and other materials. This would also involve quantifying adhesion and peel strength the activated emulsion against materials found in electronic applications. Studying the effect of high current on our coalesced ink with metals is also important in understanding its versatility as a printed conductor, as high electrical currents are known to cause intermetallic alloy formation between pure liquid metals and copper (which is predominantly found in electronic components).^[^ ^90^ ^]^ Additionally, metals can be incorporated into the continuous phase of the emulsion to generate solid alloys,^[^ ^11^, ^16^ ^]^ opening up an alternative way to mildly synthesize and pattern solid metal alloy geometries.

We note that the safety and environmental impact of organohalides is an open topic. Though it is known that some organohalides are ‘persistent’ and hazardous,^[^ ^91^ ^]^ thousands of organohalides are found in nature and are able to degrade naturally.^[^ ^92^, ^93^ ^]^ Furthermore, not all organohalides are toxic and are used for health benefits. For example, 2B2c is used in the process of making tulobuterol, a drug extensively used to treat asthma or chronic obstructive pulmonary disease,^[^ ^94^ ^]^ indicating a low level of safety and environmental concern. Moreover, since our chemical coalescing technology effectively neutralizes 2B2c, it should be relatively safe to use.

## 4 Conclusion

In this work, we synthesize a liquid metal emulsion ink that chemically activates with a unique catalytic dehalogenation and chemical oxide etching chemistry. We study the effect of altering D140 rosin and 2B2c concentration on emulsion post‐heat conductivity and printability, leading us to an optimum formulation (80 v/v% eGaIn, 0.7 v/v% D140, and 4.5 v/v% 2B2c) that balances functionality and manufacturability. Rheology characterization of our optimum ink shows that it has the required viscoelastic behavior for DIW, with shear thinning and shear yielding properties along with a high storage modulus and high yield stress. The emulsion ink requires high extrusion pressure and high print velocities, making it conducive to scaled up automated additive manufacturing methods. We also show that the emulsion ink is able to span millimeter long distances. Finally, we demonstrate the utility of our optimum ink as SMD electrical interconnects and battery current collector in the hybrid 3D printing and mild heat treatment of a complex LED array without destruction of device components. This technology not only enables simpler fabrication of GaLMA‐based applications but it also opens opportunities up for patterning soft metal catalysts.

## 5 Experimental Section

### Materials

Covalent halide‐based emulsion formulations consisted of the following ingredients: eGaIn (METSPEC60, 5N), diphenyl ether (DE) (Sigma Aldrich), highly dimerized gum rosin (D140) (Teckros®D140, Teckrez), and 2‐bromo‐2'‐chloroacetophenone (2B2c) (Sigma Aldrich). The emulsions with the halide salt activator (Figure S2, Supporting Information) consisted of eGaIn, DE, gum rosin (Sigma Aldrich), dimethylammonium chloride (DAC) (Sigma Aldrich) and deionized water (Direct Q3 UV, Millipore). The adhesive pads in Figure 4 were printed using DOWSIL^TM^ SE1700 part A (Dow).

### NMR Spectroscopy

All ^1^H NMR measurements were taken with 64 scans in Acetone‐D6 (99.9% atom D, Thermo Scientific Chemicals) with an Agilent 500 MHz VNMRS Spectrometer. Chemical shifts were measured relative to 0.03% tetramethyl silane. To monitor the reaction of 2B2c and eGaIn, a droplet of eGaIn (0.5 g) was combined with 2B2c (75 µL) and bath sonicated for 45 minutes. The mixture was heated for 1 h at 80 °C, and the reacted 2B2c (with possible trace amounts of eGaIn) (30 µL) was sampled for characterization by ^1^H NMR.

### X‐ray Photoelectron Spectroscopy (XPS)

Emulsion samples for XPS were synthesized as described in *Ink Preparation* and printed on a silicon wafer chip (1319, University Wafer) using a custom laser cut stencil made out from an acrylic film (0.01″ thickness, McMaster Carr). Heated samples were mounted onto a glass slide for ease of handling. Characterization through XPS was carried out on Nexsa G2, an integrated X‐ray system from Thermo Scientific Inc., allowing the high‐throughput surface analysis to be conducted on the material surface (<10 nm). The spot size of the X‐ray that shined on the material surface is 100 µm in diameter in these measurements, the equivalent focus voltage on the sample is 975 V. The survey spectrum was acquired under 200 eV pass energy, 5 times acquisition and 1.0 eV energy step size to give a general view of the surface composition. High‐resolution scans for each component, Ga, Br, Cl, and In, were acquired under 10 eV, 15 times acquisition and 0.3 eV energy step size to provide more precise information of the chemical states. As for other elements that also come from the environment, O and C, the high‐resolution scans were acquired under 10 eV, 10 times acquisition and 0.5 eV energy step size for calibration purpose.

### Ink Preparation

Covalent halide‐based emulsion formulations were prepared as follows. First, a premixture of DE and D140 rosin is made by grinding D140 rosin via mortar and pestle to create a fine powder. The D140 rosin and DE are subsequently shear mixed via dual asymmetric centrifuge (SpeedMixer model DAC 150.1 FVZ‐K, Flacktek) at 3500 RPM to dissolve the rosin until a particle‐free translucent mixture is obtained. Next, the DE/D140 rosin premixture is combined with 2B2c and shear mixed at 3500 RPM for 30 s. Last, eGaIn is added to the DE/D140/2B2c mixture and shear mixed at 3500 RPM in 2.5 min increments. For stencil‐printed samples in Figure 2b, formulations were synthesized to a volume of ∼1.2 mL and mixed until a homogeneous emulsion was achieved. These mixing times are listed in Table S3 (Supporting Information). For printability experiments (Figure 3a), formulations were synthesized to a volume of ∼1.75 mL and mixed longer than the stated mixing times in Table S3 (Supporting Information) to increase its stiffness. The halide salt activator‐based emulsion shown in Figure S2 (Supporting Information) as follows. First two mixtures are synthesized: 1) a ∼75 w/w% DAC/water solution (DAC (*aq*)), (which is close to saturation of DAC in water) and a (2) ∼52 w/w% rosin/DE mixture. A premixture was then made by combining DAC (*aq*) solution and rosin/DE mixture in a mass ratio of 9.5 g rosin/DE mixture to 1 g DAC solution and shear mixing at 3500 RPM for 1 min. EGaIn is then added to the premixture at a mass ratio of ∼24.2 g eGaIn to 1 g rosin/DE/DAC solution premixture and shear mixed (1750 RPM for 3 min followed by a high speed mix of 3500 RPM for 45 s).

### Ink Conductivity Testing and Analysis

Each formulated ink was patterned into a thin rectangle (35 mm × 2 mm) (*N* = 4) using a custom laser cut stencil made out from an acrylic film (0.01″ thickness, McMaster Carr). Each ink was patterned on top of two conductive copper foil tapes (25 mm long, $\frac{1}{4}$″ wide, 0.0035″ thick, McMaster Carr) spaced 25 mm apart and on top of black polyimide tape (1 mil thick, 3″ width, Bertech) adhered to a stainless‐steel plate. Black polyimide was used due to both ink chemistry compatibility and to provide sufficient optical contrast for 3D scanning. Each sample was heated in an oven (Heratherm, Thermo Scientific) at *T* ∼ 80 °C for one hour. Before and after heating, the two‐point probe resistance and 3D point cloud was collected for each sample. The two‐point probe resistance was measured using a source meter (Keithley 2401) with Kelvin probes (Keithley 5806) using the four‐point probe setting on the source meter. High resolution (12.5 µm) point clouds were measured using a laser profiler (LJ‐X8080, Keyence) mounted onto an axis of a custom gantry motion system (AGB 10000, Aerotech) with a motion controller (A3200, Aerotech). A custom Python code (using pandas, MeCode, and Keyence packages) controlled the movement of the laser profiler and synchronized the *y‐z* position from the sensor with the gantry's *x* position for point cloud construction. 3D scans were processed by implementing another custom Python code (pandas, NumPy, Open 3D and SciPy packages) that automated extraction of the patterned liquid metal trace through random sample consensus algorithm and point cloud outlier clean‐up through density‐based clustering. This workflow is summarized in Figure S13 (Supporting Information). Electrical conductivity (σ) on the processed point cloud utilized the following equation, which is a discretized form of the resistivity equation (this derivation can be found in the Supporting Information):

(1) $$\sigma =\frac{l}{R_{\text{meas}}}\sum_{i=1}^{N}\frac{1}{A_{i}}$$

where *l* is the conduction length of a trace slice that we set to 12.5 µm, *R*_meas_ is the measured resistance after heating, and *A*_*i*_ is the calculated area (through the trapezoidal rule) of a cross sectional slice of an emulsion sample's point cloud.

### Imaging

For scanning electron microscope (SEM) images, samples were stencil patterned using a thin acrylic stencil (as described in *Ink Conductivity Testing and Analysis* section) on a silicon wafer chip (1319, University Wafer). For the images shown in Figure 2, samples were imaged using a Zeiss Supra 55VP Field Emission SEM using an accelerating voltage = 15 kV, secondary electron and in‐lens detector, ∼11.2–11.7 mm working distance. To obtain droplet size distribution, samples were imaged using a ThermoFisher Phenom PROX Desktop SEM using an accelerating voltage = 10 kV, an electron backscatter detector (EBSD), and working distances between ∼6.5−7.5 mm. For optical images, shown in Figure S21 (Supporting Information), emulsion samples were stencil patterned the same (using a thin acrylic stencil) on a glass substrate covered with black Kapton tape and imaged using Keyence confocal microscope model: VK‐X3050 at 20x zoom.

### Droplet Size Distribution

Droplet size distribution shown in Figure S12 was carried out on unheated emulsions that contained ∼0.7 v/v% D140 rosin and 0, 2.5 and 4.5 v/v% 2B2c. FIJI (ImageJ) was used to characterize the collected SEM image. To obtain a clear segmentation of the droplets, the original image was enhanced by adjusting brightness and contrast and was despeckled 3–5 times to remove the detailed wrinkles on the oxide skin of each droplet. Trainable Waikato Environment for Knowledge Analysis [‘Weka'] Segmentation (TWS) plugin from FIJI (ImageJ), using the default classifier (FastRandomForest) was then applied to the enhanced image. The TWS plugin requires user input to define droplet and nondroplet regions. After the desirable segmentation is achieved (Figure S12, Supporting Information, third row), we obtained droplet diameter by using the “Analyze Particles” function in FIJI (ImageJ), which resulted in the blue histogram (Figure S12, Supporting Information, bottom) for each formulation. We applied a log‐normal fit to the calculated droplet diameters using Python (SciPy package) to obtain the mean and standard deviation reported in the figure. Mean droplet size is calculated through the equation: $e^{\left(\mu +\frac{\sigma^{2}}{2}\right)}$, where μ = ln(*m*) with μ and *m* defined as the mean of the lognormal distribution and median of the distribution, respectively.

### Ink Printability Experiments

All tested emulsions were synthesized to a volume of ∼1.75 mL as outlined in the *Ink Preparation* section. Print tests were performed on a custom three‐axis gantry motion system (AGB 10000, Aerotech) with a motion controller (A3200, Aerotech). To test for printability, each formulation is mixed ∼10 min longer than the stated mix times in Table S3 (Supporting Information) to achieve a more homogeneous consistency for 3D printing, but is kept to a mix time that will prevent it from creaming if mixed for too long (Figure S19, Supporting Information). Once a formulation reached an optimal consistency, it was loaded into a syringe equipped with a tapered nozzle (*D* = 200 µm; Nordson, EFD). A high pressure adapter (HPx High‐Pressure Dispensing Tool, Nordson EFD) connected to a pressure controller (Ultimus V High Precision Dispenser, Nordson, EFD) was used to extrude the ink into a serpentine pattern (66.2 mm × 38 mm) (see Figure S17, Supporting Information). The height between the nozzle and substrate was maintained at 196 µm and the print velocity was 70 mm s^−1^. The minimum pressure enabling consistent ink flow through the nozzle was chosen for printing multiple serpentine patterns and this pressure was kept constant until the ink was depleted and/or could no longer extrude ink. Pressures ranged from 35–84 PSI and varied between formulations due to particular formulations requiring higher pressures to maintain constant flow.

### Ink Rheology

The optimized emulsion (composition: ∼80 v/v% eGaIn, ∼0.7 v/v% D140 and ∼4.5 v/v% 2B2c) and premixture (continuous phase composition of ∼89.5 v/v% DE, ∼3.5 v/v% D140, and ∼27 v/v% 2B2c) were characterized at *T* = 25 °C using a hybrid rheometer (TA Instruments Discovery HR‐1). A parallel plate geometry (20 mm plate diameter, 500 µm working gap) was used for the emulsion, while a cone plate geometry (40 mm plate diameter, 1°, 28 µm working gap) was used for the premixture. Before every rheological measurement, the sample was pre‐sheared for 1 minute at 0.1% strain at an angular frequency (ω) of 10 rad s^−1^. Oscillation amplitude sweep experiments were performed using a frequency *f* = 1 Hz (ω = 6.3 rad s^−1^) and logarithmically sweeping the stress from 0.01 to 8952 Pa. Oscillation frequency sweep experiments were performed fixing the strain amplitude (γ) to 1%. Viscosity data was obtained by performing a flow sweep test, logarithmically sweeping the shear rate from 0.01–1000 s^−1^.

### Construction of Print Phase Diagram

The same emulsion ink composition used for rheology experiments (Figure 3b) was used for construction of the print phase diagram (Figure 3c,d). For printing, the same set‐up for ink extrusion was used as listed in the *Ink Printability Experiments* section. A velocity sweep ranging from *V* = 40–120 mm s^−1^ was carried out, keeping the nozzle to glass substrate distance (*H*) = 0.5 mm to print 50 mm long filaments. A microscope camera (uEye, IDS) was used to film the filament extrusion. The video frames shown in Figure 3c are taken at the midpoint of the printed filament. Filaments were then 3D scanned using same motion‐controlled laser profiler system mentioned in the *Ink Conductivity Testing and Analysis* section with a resolution of 12.5 µm. To estimate average filament diameter, *d*, for a filament sample, plane segmentation function using Python (Open3D package) is used for separating the filament points from the substrate/plane points. The *z*‐coordinates of the filament points are then normalized by subtracting the *z*‐coordinates from the substrate plane, and the maximum height along the length of the filament scan is calculated and aggregated for each sample (*d*). Python is then used to perform a nonlinear least squares fitting (optimize.curve_fit, SciPy) to all the *d* data (normalized by the nozzle diameter, *D*) at each print velocity (*V*) to the following piece‐wise equation (NumPy):

(2) $$\frac{d}{D}=\left\{\begin{array}{cc} \alpha ; & V\leq C\\ \frac{\alpha }{\sqrt{V/C}}; & V\geq C \end{array}\right.$$

Initial guesses for *C* ranging from 50–120 mm s^−1^ and α = 1.3 were supplied to the fitting function. For each *C* guessed, the calculated *C* = 88.91 ± 0.10 and α = 1.02 ± 0.0002.

### Determination of *G*′ from Spanning Structures

For the triangle with spanning lines shown in Figure 3e, the same emulsion ink composition used for rheology experiments (Figure 3b) was used, as well as the same print set‐up as listed in the *Ink Printability Experiments* section. The spanning structure shown in Figure 3e utilized a combination of slow and fast print speeds at a constant applied pressure. The base of the triangle, which was 0.59 mm high, utilized a *V* = 60 mm s^−1^ (V* = 0.67), while each spanning element of length *L* was printed at *V* = 90 mm s^−1^ (V* = 1.01) to decrease the effect of coiling. Interfilament layer height was 0.98*D*. The structure was then 3D scanned with the same motion‐controlled laser profiler system mentioned in the *Ink Conductivity Testing and Analysis* section with a resolution of 12.5µm. Spanned lines of different lengths *L* were segmented from the raw 3D scan of the printed triangle further cleaned using noise filter of k‐nearest neighbors = 6 to remove 3D scanning point artifacts using CloudCompare (www.cloudcompare.net). To calculate the *z*‐deflection (δz) of each spanned line, a custom Python code was used to fit a plane (*z*_topplane_) to the highest *z* value for every *x*, *y* coordinate above each beam support corresponding to each segmented spanned line. The raw *z* coordinates for each segmented spanned line were then subtracted from the *z* coordinates in (*z*_topplane_), resulting in a point cloud representing the *z*‐deflection in 3D space for each spanned line. To obtain the 2D δz profiles presented in Figure 3f, δz at a given x‐coordinate was averaged along the *y*‐coordinate for each spanned line's δz point cloud and cropped at the *x*‐coordinate of the inner part of the support on each end. The storage modulus, *G*′, for the printed emulsion was estimated by utilizing a custom Python code (optimize.curve fit, SciPy) to fit the 2D δz profile data of all spanned lines (prepared as one array containing *x*,*L*, and δz to the following piece‐wise equation^[^ ^76^ ^]^:

(3) $$\delta z=\left\{\begin{array}{ccc} -\frac{W}{24EI}{\left[{\left(\frac{L}{2}\right)}^{2}-x^{2}\right]}^{2}; & -\frac{L}{2}\leq x\leq -\frac{L}{2}+\delta & \text{(bending)}\\ & \frac{L}{2}-\delta \leq x\leq \frac{L}{2} & \\ \left(\frac{x^{2}}{2}-\frac{L^{2}}{8}\right){\left(\frac{24W}{EAL^{2}}\right)}^{1/3}; & -\frac{L}{2}+\delta \leq x\leq \frac{L}{2}-\delta & \text{(stretching)} \end{array}\right.$$

where the bending boundary layer is scaled by the relation $\delta ∼{\left(\frac{Ed^{5}}{WL}\right)}^{1/3}$. This equation represents the equilibrium viscoelastic catenary model derived by Zhu and Smay;^[^ ^76^ ^]^ where *W* is the distributed weight of the filament given by the equation *W* = 0.25(ρ_*ink*_ − ρ_*air*_)*g*_0_π*d* ^2^ (ρ is the density of ink or air, *g*_0_ is the gravitational constant and *d* is the filament diameter), *L* is the length of the spanned filament (noted in gray for each spanned line subplot), *I* is the area moment of inertia, assuming the filament has a circular cross section, *A* is the filament cross sectional area, and *E* is the Young's modulus of the ink provided by the equation *E* = 2*G*′(1 + ν). We assume the emulsion is incompressible and thus set the Poisson's ratio, ν, to 0.5, allowing us to estimate *G*′. More details can be found in Note S12 (Supporting Information) regarding estimation of δ and the values of the other parameters used in the viscoelastic catenary model.

### Hybrid 3D Printing

All inks were printed using a custom three axis gantry system (AGB 10000, Aerotech) with four independently controlled *z*‐axis (Aerotech Inc.). The emulsion ink and the adhesive ink (SE1700, part A) were both equipped with a tapered nozzle (Gauge 27, Nordson, EFD) and each loaded to a separate *z*‐axis. The emulsion ink was extruded at 91 PSI using a high‐pressure dispensing tool (HP3cc, Nordson, EFD) connected to a pressure controller (Ultimus V, Nordson EFD), and a speed of 60 mm s^−1^. The adhesive ink was printed at a pressure of 75.3 PSI using a custom solenoid (VQD1151‐5MO‐ M5, SMC Pneumatics) array, and a speed of 10 mm s^−1^. The solenoid array was connected to a pressure controller (Alicat), capable of pressures ranging from –14.7 to 85.3 PSI. Automated pick‐and‐place (AP+P) of electronic components was performed using two empty syringes mounted on two separate *z*‐axis, with each syringe connected to the solenoid array. For AP+P of LEDs and the resistors, the empty syringe is equipped with an 0.51 mm metal capillary nozzle (Gauge 21, Nordson, EFD). For AP+P of the battery, the empty syringe is connected to a 1.36 mm metal capillary nozzle (Gauge 15, Nordson, EFD). A custom 3D printed tray stores the electrical components, with each component having its own cavity. The pressure in the pressure controller connected to the solenoid array is set to –14.7 PSI to create a vacuum. The empty nozzles are then translated to the desired *x, y* position of the component and lowered in the *z* direction until it is right above the component. The solenoid valve connected to the desired empty syringe is then turned on, activating the vacuum, and picking up the component. The component is then translated to the desired position and the vacuum is released. All custom print‐paths were created using open‐source Python libraries (MeCode), and loaded to the Aerotech controller (A3200, Aerotech).

### LED Array

The process for hybrid 3D printing the LED array can be seen in Video S5 (Supporting Information). Briefly, the LED array consisted of 19 LEDs (Red, 0603, Kingbright), 19 resistors (300 Ohm, 0603, ROHM Semiconductor), and a high temperature (*T*_max_ = 85 °C) battery (3V, 2450X, Murata). Each LED is connected in series to a resistor, and all LED and resistor pairs are connected in parallel to the battery. The battery powered LED array was fabricated by first printing pads of the adhesive ink in the location of each LED and resistor. This was followed by printing a pad of the emulsion ink at each side of the adhesive pad, to interface with the LED and resistor electrodes. LEDs and resistors were then automatically picked‐and‐placed on top of the printed adhesive emulsion ink pads. The emulsion ink was then used to print electrical interconnects to all the components and interconnects to the LED array power and ground lines with the battery's power (cathode) and negative (anode) terminals. A lattice was then printed with the emulsion to connect with the bottom part of the battery (anode), and a pillar was printed to connect to the top part of the battery (cathode). The battery is then picked‐and‐placed on top of the lattice. The emulsion is subsequently printed on top of the battery to connect the battery cathode to the previously printed pillar. The device was then placed in an environmental chamber (Tenney, Model TJR‐A‐F4T) at 80 °C until all LEDs lit up (∼17 hours).

### Statistical Analysis

The post‐heat conductivity data in Figure 2b, was calculated by Equation S27 (Supporting Information). *R*_meas_ (measured resistance) was used as is. The cross sectional slice of an ink formulation sample point cloud, *A*_*i*_, was obtained by removing points belonging to the substrate by using RANSAC^[^ ^95^ ^]^ and DBSCAN^[^ ^96^ ^]^ algorithms. The reported data represents the mean ± S.E.M. (standard error of the mean) using a sample size of four (*N* = 4). Python (pandas, NumPy, Open 3D and SciPy packages) was used for all data processing.

The normalized filament data (*d*/*D*) in Figure 3d is obtained by performing plane segmentation on the sample point cloud using Python (Open3D package) to separate the printed filament points from the substrate/plane points. The *z*‐coordinates of the filament points are normalized by subtracting the *z*‐coordinates from the substrate plane, and the maximum height along the length of the filament scan is calculated and aggregated for each sample (d). The reported data represents the mean ± S.E.M. using a sample size of three (*N* = 3). Python (pandas, NumPy, Open 3D and SciPy packages) was used for all data processing.

## Conflict of Interest

The authors declare no conflict of interest.

## Supporting information

Supporting Information

Supplemental Video 1

Supplemental Video 2

Supplemental Video 3

Supplemental Video 4

Supplemental Video 5

## Data Availability

The data that support the findings of this study are available from the corresponding author upon reasonable request.

## References

[1] M. D. Dickey , R. C. Chiechi , R. J. Larsen , E. A. Weiss , D. A. Weitz , G. M. Whitesides , Adv. Funct. Mater. 2008, 18, 1097.

[2] S. Cheng , A. Rydberg , K. Hjort , Z. Wu , Appl. Phys. Lett. 2009, 94, 14.

[3] J. Koster , Cryst. Res. Technol. 1999, 34, 1129.

[4] M. Hodes , R. Zhang , L. S. Lam , R. Wilcoxon , N. Lower , IEEE T Comp. Pack. Man. 2013, 4, 46.

[5] D. Zrnic , D. Swatik , J. Less‐Common Met. 1969, 18, 67.

[6] M. Dickey , Adv. Mater. 2017, 29, 27. 10.1002/adma.201606425 28417536

[7] M. R. Khan , C. B. Eaker , E. F. Bowden , M. D. Dickey , PNAS 2014, 111, 14047. 25228767 10.1073/pnas.1412227111 PMC4191764

[8] S. Holcomb , M. Brothers , A. Diebold , W. Thatcher , D. Mast , C. Tabor , J. Heikenfeld , Langmuir 2016, 32, 12656. 27934511 10.1021/acs.langmuir.6b03501

[9] J. Liao , C. Majidi , M. Sitti , Adv. Mater. 2024, 36, 2300560. 10.1002/adma.202300560 37358049

[10] X. Ye , Z. Zheng , J. G. Werner , J. W. Boley , Adv. Funct. Mater. 2023, 2309177.

[11] K. Kalantar‐Zadeh , T. Daeneke , J. Tang , Science 2024, 385, 372. 39052810 10.1126/science.adn5871

[12] Y. Lin , J. Genzer , M. D. Dickey , Adv. Sci. 2020, 7, 2000192. 10.1002/advs.202000192 PMC7312306 32596120

[13] L. R. Bernstein , Pharmacol. Rev. 1998, 50, 665. 9860806

[14] J. Yan , Y. Lu , G. Chen , M. Yang , Z. Gu , Chem. Soc. Rev. 2018, 47, 2518. 29557433 10.1039/C7CS00309A

[15] N. Kazem , T. Hellebrekers , C. Majidi , Adv. Mater. 2017, 29, 1605985. 10.1002/adma.201605985 28425667

[16] G. Cao , J. Liang , Z. Guo , K. Yang , G. Wang , H. Wang , X. Wan , Z. Li , Y. Bai , Y. Zhang , et al., Nature 2023, 619, 73. 37316660 10.1038/s41586-023-06082-9

[17] J. W. Boley , E. L. White , R. K. Kramer , Adv. Mater. 2015, 27, 2355. 25728533 10.1002/adma.201404790

[18] M. G. Mohammed , R. Kramer , Adv. Mater. 2017, 29, 1604965. 10.1002/adma.201604965 28247998

[19] J. W. Boley , W. M. van Rees , C. Lissandrello , M. N. Horenstein , R. L. Truby , A. Kotikian , J. A. Lewis , L. Mahadevan , Proc. Natl. Acad. Sci. 2019, 116, 20856. 31578256 10.1073/pnas.1908806116 PMC6800333

[20] T. Neumann , E. Facchine , B. Leonardo , S. Khan , M. Dickey , Soft Matter 2020, 16, 6608. 32613217 10.1039/d0sm00803f

[21] R. Sánchez Cruz , S. F. Zopf , J. W. Boley , J. Compos. Mater. 2023, 57, 829.

[22] Z. Lin , X. Qiu , Z. Cai , J. Li , Y. Zhao , X. Lin , J. Zhang , X. Hu , H. Bai , Nat. Commun. 2024, 15, 4806. 38839743 10.1038/s41467-024-48906-w PMC11153652

[23] X. Li , M. Li , J. Xu , J. You , Z. Yang , C. Li , Nat. Commun. 2019, 10, 3514. 31383861 10.1038/s41467-019-11466-5 PMC6683165

[24] S. Ahmed , M. Momin , J. Ren , H. Lee , T. Zhou , Adv. Mater. 2024, 2400082. 10.1002/adma.202400082 38563579

[25] N. J. Morris , Z. J. Farrell , C. E. Tabor , Nanoscale 2019, 11, 17308. 31513218 10.1039/c9nr06369b

[26] J. D. Bernal , J. Mason , Nature 1960, 188, 910.

[27] A. Fassler , C. Majidi , Adv. Mater 2015, 27, 1928. 25652091 10.1002/adma.201405256

[28] C. J. Thrasher , Z. J. Farrell , N. J. Morris , C. L. Willey , C. E. Tabor , Adv. Mater. 2019, 31, 1903864. 10.1002/adma.201903864 31403234

[29] S. Liu , M. C. Yuen , E. L. White , J. W. Boley , B. Deng , G. J. Cheng , R. Kramer‐Bottiglio , ACS Appl. Mater. Inter. 2018, 10, 28232. 10.1021/acsami.8b08722 30045618

[30] E. J. Frey , S. Im , A. L. Bachmann , J. Genzer , M. D. Dickey , Adv. Funct. Mater. 2024, 34, 2308574.

[31] S. Liu , S. N. Reed , M. J. Higgins , M. S. Titus , R. Kramer‐Bottiglio , Nanoscale 2019, 11, 17615. 31274138 10.1039/c9nr03903a

[32] S. Liu , D. S. Shah , R. Kramer‐Bottiglio , Nat. Mater. 2021, 20, 851. 33603186 10.1038/s41563-021-00921-8

[33] W. Lee , H. Kim , I. Kang , H. Park , J. Jung , H. Lee , H. Park , J. S. Park , J. M. Yuk , S. Ryu , J. Jiang , J. Kang , Science 2022, 378, 637. 36356149 10.1126/science.abo6631

[34] W. M. Haynes , CRC handbook of chemistry and physics, CRC Press, Boca Raton, FL 2014.

[35] G.‐F. Chen , Prog. Organic Coatings 1992, 20, 139.

[36] Teckrez, LLC , Resin Product Guide , Rev 2‐24.

[37] L. Spitz , Soap manufacturing technology, Elsevier, New York 2016.

[38] P. Benson , D. Brining , D. Perrin , Marine fouling and its prevention Marine Technology Society 1973, p. 30–37.

[39] S. Jadhav , Open Chem. 2011, 9, 369.

[40] Q. Wei , M. Sun , Z. Wang , J. Yan , R. Yuan , T. Liu , C. Majidi , K. Matyjaszewski , ACS Nano 2020, 14, 9884. 32649179 10.1021/acsnano.0c02720

[41] N.‐C. Lee , Reflow Soldering Processes and Troubleshooting, Newnes, Australia 2002.

[42] H. Nozaki , T. Shirafuji , K. Kuno , Y. Yamamoto , B. Chem. Soc. Jpn. 1972, 45, 856.

[43] L. Park , G. Keum , S. B. Kang , K. Soo Kim , Y. Kim , J. Chem. Soc. Perk. T. 1 2000, 4462.

[44] A. Cañete , C. Salas , F. Zacconi , Molecules 2012, 18, 398. 23275048 10.3390/molecules18010398 PMC6270286

[45] T. Daeneke , K. Khoshmanesh , N. Mahmood , I. A. De Castro , D. Esrafilzadeh , S. J. Barrow , M. D. Dickey , K. Kalantar‐Zadeh , Chem. Soc. Rev. 2018, 47, 4073. 29611563 10.1039/c7cs00043j

[46] P. C. St. John, Y. Guan , Y. Kim , S. Kim , R. S. Paton , Nat. Commun. 2020, 11, 2328. 32393773 10.1038/s41467-020-16201-z PMC7214445

[47] P. C. St. John, Y. Guan , Y. Kim , B. D. Etz , S. Kim , R. S. Paton , Sci. Data 2020, 7, 244. 32694541 10.1038/s41597-020-00588-x PMC7374734

[48] L. Cademartiri , M. M. Thuo , C. A. Nijhuis , W. F. Reus , S. Tricard , J. R. Barber , R. N. S. Sodhi , P. Brodersen , C. Kim , R. C. Chiechi , G. M. Whitesides , J. Phys. Chem. C 2012, 116, 10848.

[49] B. H. Freeland , J. J. Habeeb , D. G. Tuck , Can. J. Chem. 1977, 55, 1527.

[50] D. Kim , P. Thissen , G. Viner , D. Lee , W. Choi , Y. Chabal , J. Lee , ACS Appl. Mater. Inter. 2013, 5, 179. 10.1021/am302357t 23206334

[51] E. Papirer , R. Lacroix , J.‐B. Donnet , G. Nanse , P. Fioux , Carbon 1994, 32, 1341.

[52] F. Scharmann , G. Cherkashinin , V. Breternitz , C. Knedlik , G. Hartung , T. Weber , J. A. Schaefer , Surf. Interface Anal. 2004, 36, 981.

[53] J. C. Fan , J. B. Goodenough , J. Appl. Phys. 1977, 48, 3524.

[54] P. Kowalik , P. Bujak , M. Penkala , A. Maroń , A. Ostrowski , A. Kmita , M. Gajewska , W. Lisowski , J. Sobczak , A. Pron , Chem. Mater. 2022, 34, 809. 35095188 10.1021/acs.chemmater.1c03800 PMC8794001

[55] A. Rossi , B. Elsener , N. Spencer , Spectroscopy Europe 2004, 16, 14.

[56] T. Mason , J. Bibette , D. Weitz , Phys. Rev. Lett. 1995, 75, 2051. 10059196 10.1103/PhysRevLett.75.2051

[57] O. Behrend , K. Ax , H. Schubert , Ultrason. Sonochem. 2000, 7, 77. 10769874 10.1016/s1350-4177(99)00029-2

[58] Y. Lin , C. Cooper , M. Wang , J. J. Adams , J. Genzer , M. D. Dickey , Small 2015, 11, 6397. 26568095 10.1002/smll.201502692

[59] J. Yan , M. H. Malakooti , Z. Lu , Z. Wang , N. Kazem , C. Pan , M. R. Bockstaller , C. Majidi , K. Matyjaszewski , Nat. Nanotechnol. 2019, 14, 684. 31110266 10.1038/s41565-019-0454-6

[60] L. R. Finkenauer , Q. Lu , I. F. Hakem , C. Majidi , M. R. Bockstaller , Langmuir 2017, 33, 9703. 28845991 10.1021/acs.langmuir.7b01322

[61] J. Park , S. Wang , M. Li , C. Ahn , J. K. Hyun , D. S. Kim , D. K. Kim , J. A. Rogers , Y. Huang , S. Jeon , Nat. Commun. 2012, 3, 916. 22735444 10.1038/ncomms1929

[62] S. Zhu , J.‐H. So , R. Mays , S. Desai , W. R. Barnes , B. Pourdeyhimi , M. D. Dickey , Adv. Funct. Mater. 2013, 23, 2308.

[63] U. Daalkhaijav , O. D. Yirmibesoglu , S. Walker , Y. Mengüç , Adv. Mater. Technol. 2018, 3, 1700351.

[64] R. Xing , J. Yang , D. Zhang , W. Gong , T. V. Neumann , M. Wang , R. Huang , J. Kong , W. Qi , M. D. Dickey , Matter 2023, 6, 2248.

[65] T. R. Lear , S.‐H. Hyun , J. W. Boley , E. L. White , D. H. Thompson , R. K. Kramer , EML 2017, 13, 126.

[66] T. Mason , J. Bibette , D. Weitz , J. Colloid Interface Sci. 1996, 179, 439.

[67] M. R. Khan , C. Trlica , J.‐H. So , M. Valeri , M. D. Dickey , ACS Appl. Mater. Inter. 2014, 6, 22467. 10.1021/am506496u 25469554

[68] S. Datta , D. Gerrard , T. Rhodes , T. Mason , D. Weitz , Phys. Rev. E 2011, 84, 041404. 10.1103/PhysRevE.84.041404 22181143

[69] H. Yuk , X. Zhao , Adv. Mater. 2018, 30, 6. 10.1002/adma.201704028 29239049

[70] A. Franck , Understanding rheology of structured fluids , Technical report, TA Instruments.

[71] N. Spenley , M. Cates , T. McLeish , Phys. Rev. Lett. 1993, 71, 939. 10055406 10.1103/PhysRevLett.71.939

[72] G. Taylor , in Applied Mechanics: Proceedings of the Twelfth International Congress of Applied Mechanics, Stanford University, August 26–31, 1968, Springer, New York 1969, pp. 382–388.

[73] A. Geffrault , H. Bessaies‐Bey , N. Roussel , P. Coussot , Addit. Manuf. 2023, 75, 103752.

[74] N. M. Ribe , M. Habibi , D. Bonn , Annu. Rev. Fluid Mech. 2012, 44, 249.

[75] I. Girotto , A. Scagliarini , R. Benzi , F. Toschi , J. Fluid Mech. 2024, 986, A33.

[76] C. Zhu , J. E. Smay , J. Mater. Process. Tech. 2012, 212, 727.

[77] J. E. Smay , J. Cesarano , J. A. Lewis , Langmuir 2002, 18, 5429.

[78] A. D. Valentine , T. A. Busbee , J. W. Boley , J. R. Raney , A. Chortos , A. Kotikian , J. D. Berrigan , M. F. Durstock , J. A. Lewis , Adv. Mater. 2017, 29, 1703817. 10.1002/adma.201703817 28875572

[79] R. S. Khandpur , Printed Circuit Boards Design, Fabrication, and Assembly, McGraw‐Hill, New York 2006.

[80] K. B. Ozutemiz , J. Wissman , O. B. Ozdoganlar , C. Majidi , Adv. Mater. Interfaces 2018, 5, 1701596.

[81] T. Liu , P. Sen , C.‐J. Kim , J. Microelectromech. S. 2011, 21, 443.

[82] D. Aubrey , S. Ginosatis , J Adhes. 1981, 12, 189.

[83] K. Doudrick , S. Liu , E. M. Mutunga , K. L. Klein , V. Damle , K. K. Varanasi , K. Rykaczewski , Langmuir 2014, 30, 6867. 24846542 10.1021/la5012023

[84] F. Tan , X. Qiao , J. Chen , H. Wang , Int. J. Adhes. Adhes. 2006, 26, 406.

[85] Y. Marcus , Chem. Soc. Rev. 1993, 22, 409.

[86] F. Alonso , I. Beletskaya , M. Yus , Chem. Rev. 2002, 102, 4009. 12428984 10.1021/cr0102967

[87] Z. J. Farrell , C. J. Thrasher , A. E. Flynn , C. E. Tabor , ACS Appl. Nano Mater. 2020, 3, 6297.

[88] L. Zhao , Y. Yin , B. Jiang , Z. Guo , C. Qu , Y. Huang , J. Colloid. Interface Sci 2020, 573, 105. 32278169 10.1016/j.jcis.2020.03.125

[89] A. Bhattacharjya , P. Klumphu , B. H. Lipshutz , Org. Lett. 2015, 17, 1122. 25679825 10.1021/ol5037369

[90] D. K. Sarfo , R. R. Taylor , A. P. O'Mullane , ACS Appl. Electron. Mater. 2020, 2, 2921.

[91] P. R. S. Kodavanti , L. G. Costa , M. Aschner , Adv. Neurotoxicol. 2023, 10, 1. 37920427 10.1016/bs.ant.2023.06.001 PMC10622110

[92] G. W. Gribble , J. Nat. Prod. 2024, 87, 1285. 38375796 10.1021/acs.jnatprod.3c00803

[93] P. Peng , T. Goris , Y. Lu , B. Nijsse , A. Burrichter , D. Schleheck , J. J. Koehorst , J. Liu , D. Sipkema , J. S. Sinninghe Damste , et al., ISME J. 2020, 14, 815. 31896791 10.1038/s41396-019-0573-y PMC7031245

[94] H. Song , J. Lin , H. Tan , L. Shen , N. Zhang , Y. Zhang , X. Tan , Y. Yang , X. Pan , W. Zheng , J. Chromatogr. Sci. 2019, 57, 299. 30722025 10.1093/chromsci/bmy083

[95] M. A. Fischler , R. C. Bolles , Commun. ACM 1981, 24, 381.

[96] M. Ester , H.‐P. Kriegel , J. Sander , X. Xu , et al., in Proceedings of International Conference on Knowledge Discovery and Data Mining , vol. 96, 1996, pp. 226–231.

