# Supplementary material for: Self‐Catalyzed Chemically Coalescing Liquid Metal Emulsions
Source: Adv Sci (Weinh). 2025 Apr 26;12(20):2413116. doi: 10.1002/advs.202413116 (PMC12120753; doi:10.1002/advs.202413116)
Supplement: Supplementary file 1 — Supporting Information [file ADVS-12-2413116-s005.pdf]

ADVANCED SCIENCE
Open Access

Supporting Information

for *Adv. Sci.*, DOI 10.1002/advs.202413116

Self-Catalyzed Chemically Coalescing Liquid Metal Emulsions

*Stephanie F. Zopf, Ramón E. Sánchez Cruz, Chloe Kekedjian, Lu Ping, Javier M. Morales Ferrer, Jean Paul Soto Aquino, Rongxuan Xie, Xi Ling and J. William Boley**

Supporting Information for
Self-Catalyzed Chemically Coalescing Liquid Metal Emulsions

Stephanie F. Zopf, Ramon E. Sanchez Cruz, Chloe Kekedjian, Lu Ping, Javier Morales
Jean Paul Soto Aquino, Rongxuan Xie, Xi Ling, J. William Boley*

List of Supplementary Notes

Supplementary Note 1: Halide salt activator etching chemistry . . . 5
Supplementary Note 2: Chemical reaction mechanism for covalent halide-based LMEs . . . 6
Supplementary Note 3: DE and 2B2c do not react with one another . . . 7
Supplementary Note 4: Gallium and Indium Catalyze 2B2c Dehalogenation . . . 8
Supplementary Note 5: EGaIn-catalyzed Dehalogenation at Ambient Overtime . . . 9
Supplementary Note 6: Calculation for Volume Concentration in LMEs . . . 11
Supplementary Note 7: Derivation of discretized form of the resistivity equation . . . 19
Supplementary Note 8: Estimation of 2B2c Concentration Assuming 100 and 50% Dehalogenation 20
Supplementary Note 9: Calculation of LME Shrinkage . . . 23
Supplementary Note 10: Oscillation Frequency Sweep of Optimal LME . . . 31
Supplementary Note 11: Gravitational and inertial effects on filament extrusion . . . 33
Supplementary Note 12: Estimation of bending boundary layer, δ . . . 33
Supplementary Note 13: Estimation of adhesive stress for coalesced LME on SMD components . . 39
Supplementary Note 14: Calculation of LME conductivity after 10 months . . . 42

List of Supplementary Figures

S1 Carboxylic acid groups help with emulsification of eGaIn . . . 3
S2 Previous liquid metal emulsion work using halide salt activators . . . 4
S3 DE and 2B2c do not react with each other (NMR) . . . 7
S4 Detailed 2B2c dehalogenation reaction products . . . 7
S5 2B2c dehalogenation favors gallium and indium (NMR) . . . 8
S6 EGaIn catalyzed 2B2c dehalogenation at ambient over time (NMR) . . . 9
S7 Emulsion attainability formulation study based on ∼80 v/v% eGaIn . . . 10
S8 Additional XPS data for emulsion before and after being heated . . . 12
S9 XPS data for eGaIn exposed to HBr at $T = 80$°C . . . 13
S10 XPS data for eGaIn exposed to HCl at $T = 80$°C. . . . 14
S11 XPS data for eGaIn. . . . 14
S12 Droplet size distribution of unheated emulsion inks with and without 2B2c . . . 17
S13 Process for calculating conductivity of amorphous geometry . . . 18
S14 Dimensions of ideal eGaIn droplet in 80 v/v% LME . . . 20
S15 Comparison of LME to other researched liquid metal conductors . . . 22
S16 Shape fidelity shrinkage calculations . . . 23
S17 Schematic for ink extrudability testing . . . 24
S18 Effect of droplet size on rheology and printability of optimum LME . . . 25
S19 Effect of relative humidity on mixing time of liquid metal emulsion . . . 26
S20 Contact Resistance of Emulsion Ink at Higher Temperatures . . . 27
S21 Emulsion Inks at Higher Temperatures . . . 28
S22 Yield stress calculation of liquid metal emulsion . . . 29

S23 Lissajous plots . . . 30
S24 Oscillation frequency sweep . . . 31
S25 Flow curves for LME ink and ink components . . . 32
S26 Estimation of scaling factor, β, for viscoelastic spanning model . . . 33
S27 LED Schematic for BU Array . . . 35
S28 Printing LME on top of SMD electrical pins results in variable electrical connection . . . 36
S29 Design rationale for bottom lattice current collector . . . 37
S30 Structural layers contributing to shape retention and adhesion in activated LMEs . . . 38
S31 Peeling PDMS composites from coalesced LME . . . 41
S32 Comparison of 10-month aged LME to freshly soldered PCB . . . 42
S33 Circuit schematic used for calculating conductivity of 10-month aged LME . . . 42
S34 Printed LME packaged dogbone device and electromechanical testing . . . 44
S35 Thermally testing packaged LME dogbone devices . . . 44

List of Supplementary Tables

S1 Emulsion continuous phase volume concentrations . . . 11
S2 Standard enthalpies of formation for compounds in ink . . . 15
S3 Total mixing times for achieving a homogeneous liquid metal emulsion for 80 v/v% eGaIn formulations listed in main **Figure 2b**. . . . 15
S4 Variables and values used in **Equations S28 - S33**. Italicized values are constants used in calculations. Calculated values assume a total emulsion volume of 1 cm^3. . . . 21
S5 Electrical conductivity (S/cm) of liquid metal-based conductors shown in **Figure S15** . . . 22
S6 Calculated values for mean droplet size and G' for optimum LME at different mix times . . . 26
S7 Measured Copper Resistance (R_{Cu}), Calculated Contact Resistance (R_c) and Conductivity (σ) from **Figure S20** . . . 27
S8 Relaxation modulus and times obtained from model fit of **Equation S42**. . . . 31
S9 Calculated Constants used in **Equations 3, S45**, and **S48**. . . . 34
S10 Constants used in **Table S9, Equations 3, S45** and **S48**. . . . 34
S11 Variables and values used for calculating $\tau_{\text{s, battery}}$ . . . 39
S12 Variables and values used for calculating $\tau_{\text{s, LED}}$ . . . 40
S13 Alternate covalent halide activators . . . 45
S14 List of Supplementary Videos . . . 45

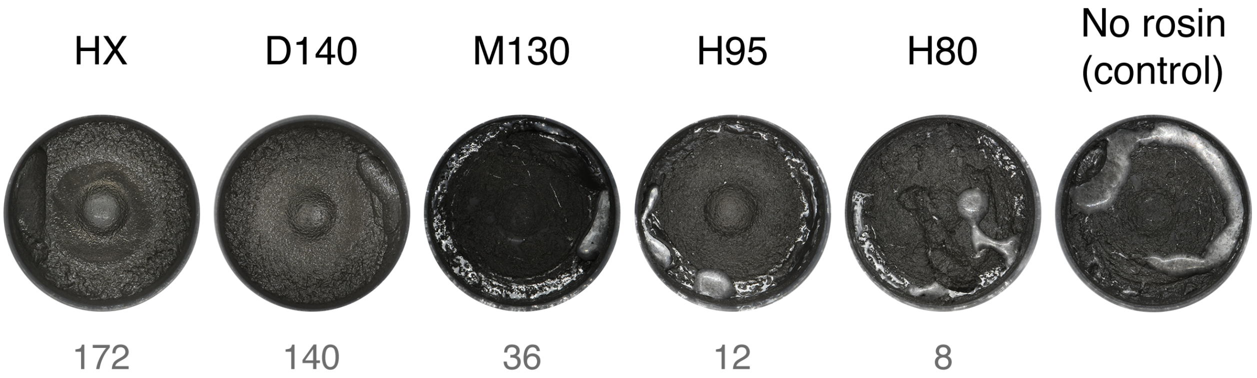

Figure S1: Top view of emulsion inks in their mixing cups, synthesized with ~80.2 v% eGaIn, ~19.7 v% and ~0.1v% rosin and shear mixed at 3500 RPM for 10 min. The type of rosin used in each emulsion is indicated above each picture and the reported acid value number (AVN), an indication of the amount of carboxylic acids present, is indicated in gray below each image. Emulsions using higher AVN (HX and D140) emulsify more readily as evidenced by the lack of unmixed eGaIn. All rosins were obtained from Teckrez, Inc.

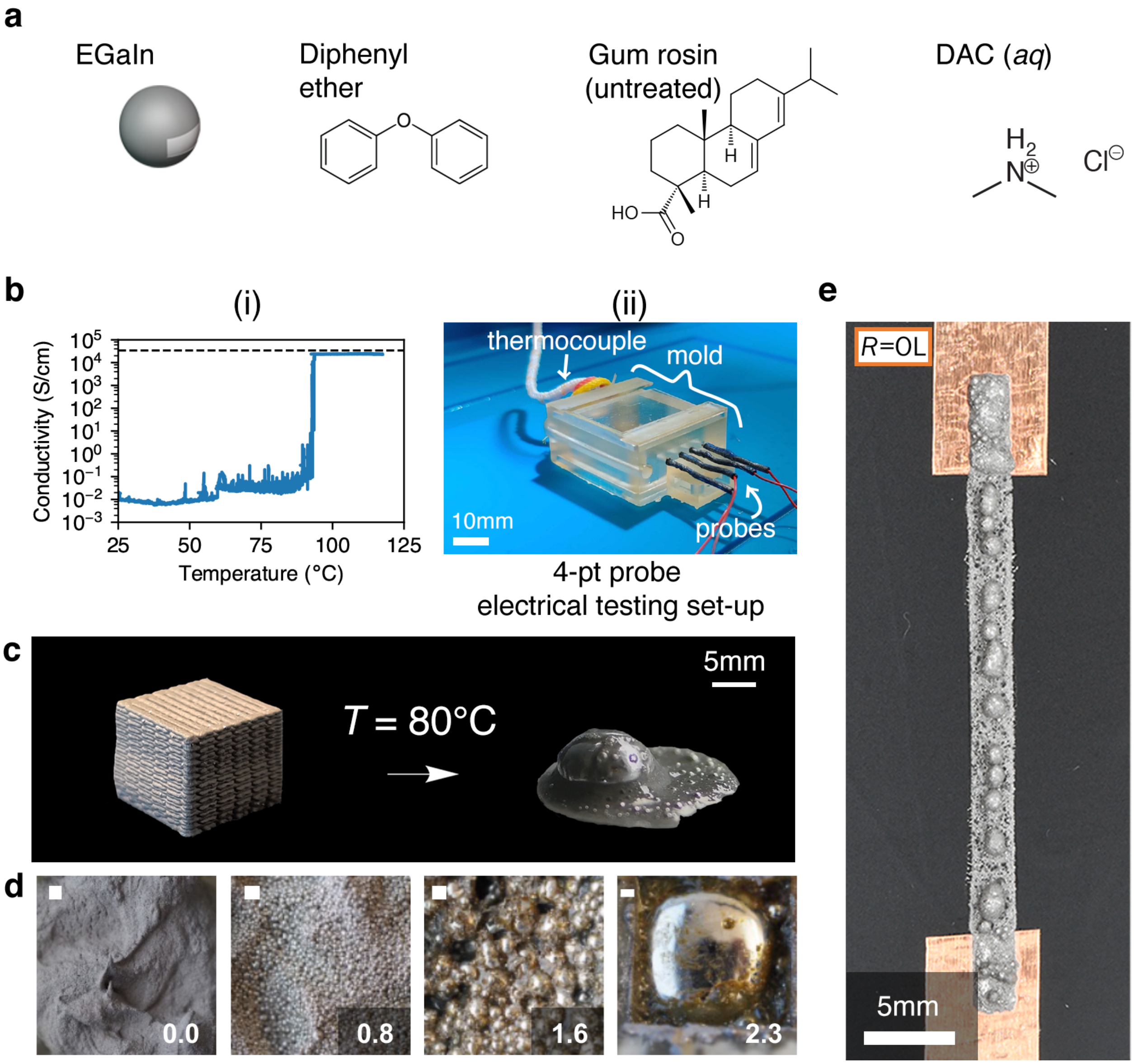

Figure S2: Liquid metal emulsion ink formulated with 80 v/v% eGaIn, DE, gum rosin (untreated), dimethylammonium chloride (DAC) salt and water. (a) Schematic and molecular structures of emulsion ink ingredients. When this emulsion is heated, droplets coalesce to conductive network of eGaIn droplets. (b-i) Four-point probe measurement results - coalescence (as indicated by conductivity increase) is initiated at 50°C, and fully conductive at ∼ 80°C. Dotted line indicates bulk conductivity of eGaIn. (b-ii) Experimental set-up of for four-point probe electrical conductivity experiment of (b-i). (c) Picture of printed lattice using emulsion ink and resulting product after being heated at T = 80°C. (d) Effect of tuning DAC activator concentration in the emulsion (number in lower right of each photo denotes v/v% DAC) The emulsion formulations were comprised of 79 v/v% eGaIn, 9.5 v/v% gum rosin (untreated), 8.3 v/v% diphenyl ether and 3 v/v% aqueous DAC salt activator solution. The scale bars on the top left denote 1mm. (e) When not confined by an external shape, the patterned halide salt liquid metal emulsions coalesce into disconnected eGaIn droplets, necessitating a solution for an activator that keeps the eGaIn droplets intact.

Supplementary Note 1: Halide salt activator etching chemistry

In halide salt activators, the halogen is readily available for combining with available hydrogen to form hydrohalide acid for chemical etching. Counter to the free halogen is an organic group, which is typically amine-based.[1] This amine-based group participates in oxidation-reduction reactions for metal oxide removal by acting as a hard Lewis base to reduce metal oxides (Ga_2O_3, In_2O_3), which are hard Lewis acids[2] to their bulk liquid metal form. One proposed scheme for the role of the organic amine in our DAC salt activator is outlined below, based on the oxidation-reaction reaction that can occur with amines and metal oxides.[1]

$$2\,(CH_3)_2NH + Ga_2O_3 \rightarrow 2\,Ga + 2\,(CH_3)CH_2OH + 2\,N_2 + H_2O \qquad (S1)$$

Supplementary Note 2: Chemical reaction mechanism for covalent halide-based LMEs

We propose that eGaIn catalyzes the dehalogenation and chemical coalescence through the following reactions. First, previous researchers[3],[4],[5] have shown that hydrogen gas is generated from the reaction of gallium-based compounds with water, namely:

$$2\,\mathrm{Ga} + 3\,\mathrm{H_2O} \rightarrow \mathrm{Ga_2O_3} + 3\,\mathrm{H_2} \tag{S2}$$

$$2\,\mathrm{Ga_2O} + 2\,\mathrm{H_2O} \rightarrow \mathrm{Ga_2O_3} + 2\,\mathrm{H_2} \tag{S3}$$

We postulate that hydrogen gas (H_2) generated from the aforementioned reactions reacts with our activator molecule, 2-bromo-2'-chloroacetophenone (2B2c) – $ClC_6H_4COCH_2Br$, with the assistance of eGaIn as a catalyst and dehalogenates (*i.e.*, releases the halogens, bromine [Br] and chlorine [Cl]). This is represented by the following reaction, where 2B2c is converted to acetophenone ($C_6H_4COCH_2$) as hydrobromic acid (HBr) and hydrochloric acid (HCl) are generated:

$$\mathrm{ClC_6H_4COCH_2Br} + 2\,\mathrm{H_2} \rightarrow \mathrm{C_6H_5COCH_3} + \mathrm{HBr} + \mathrm{HCl} \tag{S4}$$

The aforementioned reaction is validated by our NMR results in main **Figure 1**. EGaIn is known to be a liquid metal catalyst for facilitating many reactions,[6] and our NMR data in **Figure S6** with 2B2c dehalogenation in the presence of eGaIn at ambient confirms this.

It is known that acids etch metal oxides, and with our binary liquid metal alloy and dual halogen activator, the following reactions are possible:

$$\mathrm{Ga_2O_3} + 6\,\mathrm{HCl} \rightarrow 2\mathrm{GaCl_3} + 3\,\mathrm{H_2O} \tag{S5}$$

$$\mathrm{Ga_2O_3} + 6\,\mathrm{HBr} \rightarrow 2\mathrm{GaBr_3} + 3\,\mathrm{H_2O} \tag{S6}$$

$$\mathrm{In_2O_3} + 6\,\mathrm{HCl} \rightarrow 2\mathrm{InCl_3} + 3\,\mathrm{H_2O} \tag{S7}$$

$$\mathrm{In_2O_3} + 6\,\mathrm{HBr} \rightarrow 2\mathrm{GaBr_3} + 3\,\mathrm{H_2O} \tag{S8}$$

We posit that, since water is present, the metal halide salts ($\mathrm{GaBr_3}, \mathrm{GaCl_3}, \mathrm{InBr_3}\,\mathrm{and}\,\mathrm{InCl_3}$, listed in **Equations S5-S8**) undergo hydrolysis (some known reactions from references [7], [8], [9],[10] are listed below):

$$2\,\mathrm{GaCl_3} + 3\,\mathrm{H_2O} \rightarrow \mathrm{GaOHCl_2} + \mathrm{Ga(OH)_2Cl} + 3\,\mathrm{HCl} \tag{S9}$$

$$\mathrm{GaBr_3} + 2\,\mathrm{H_2O} \rightarrow \mathrm{GaOOH} + 3\,\mathrm{HBr} \tag{S10}$$

$$\mathrm{InCl_3} + \mathrm{H_2O} \rightarrow \mathrm{(InOH)Cl_2} + \mathrm{HCl} \tag{S11}$$

$$\mathrm{InBr_3} + \mathrm{H_2O} \rightarrow \mathrm{(InOH)Br_2} + \mathrm{HBr} \tag{S12}$$

These reactions generate HBr and HCl and can perform more oxide etching and thus more droplet coalescence.

The above reactions assume an oxidation state of 3+ for gallium and indium, which is dominant in water.[11] However, complexation of gallium and indium ions at other oxidation states may also be possible.[7],[10] 2B2c can also be directly reduced to produce chloride (Cl^-) and bromide (Br^-) ions, as halogenated acetophenones have been shown to undergo electrochemical reduction, forming acetylphenyl radicals and halide ions.[12] In this proposed chemical scheme, gallium (Ga^0) or indium (In^0) metal would be simultaneously oxidized to form metal halide salts (**Equations S13** and **S14**) or metal-hydroxide-halogen complexes (**Equations S15** and **S16**):

$$2\,\mathrm{Ga^0} + 3\,\mathrm{ClC_6H_4COCH_2Br} + 3\,\mathrm{H_2} \rightarrow \mathrm{GaCl_3} + \mathrm{GaBr_3} + 3\,\mathrm{C_6H_5COCH_3} \tag{S13}$$

$$2\,\mathrm{In^0} + 3\,\mathrm{ClC_6H_4COCH_2Br} + 3\,\mathrm{H_2} \rightarrow \mathrm{InCl_3} + \mathrm{InBr_3} + 3\,\mathrm{C_6H_5COCH_3} \tag{S14}$$

$$\begin{aligned} 4\,\mathrm{Ga^0} + 3\,\mathrm{ClC_6H_4COCH_2Br} + 6\,\mathrm{H_2O} \rightarrow\ & \mathrm{Ga(OH)Br_2} + \mathrm{Ga(OH)Cl_2} \\ & + \mathrm{Ga(OH)_2Br} + \mathrm{Ga(OH)_2Cl} + 3\,\mathrm{C_6H_5COCH_3} \end{aligned} \tag{S15}$$

$$4\,\mathrm{In}^0 + 3\,\mathrm{ClC_6H_4COCH_2Br} + 6\,\mathrm{H_2O} \rightarrow \mathrm{In(OH)Br_2} + \mathrm{In(OH)Cl_2} + \mathrm{In(OH)_2Br} + \mathrm{In(OH)_2Cl} + 3\,\mathrm{C_6H_5COCH_3} \quad \text{(S16)}$$

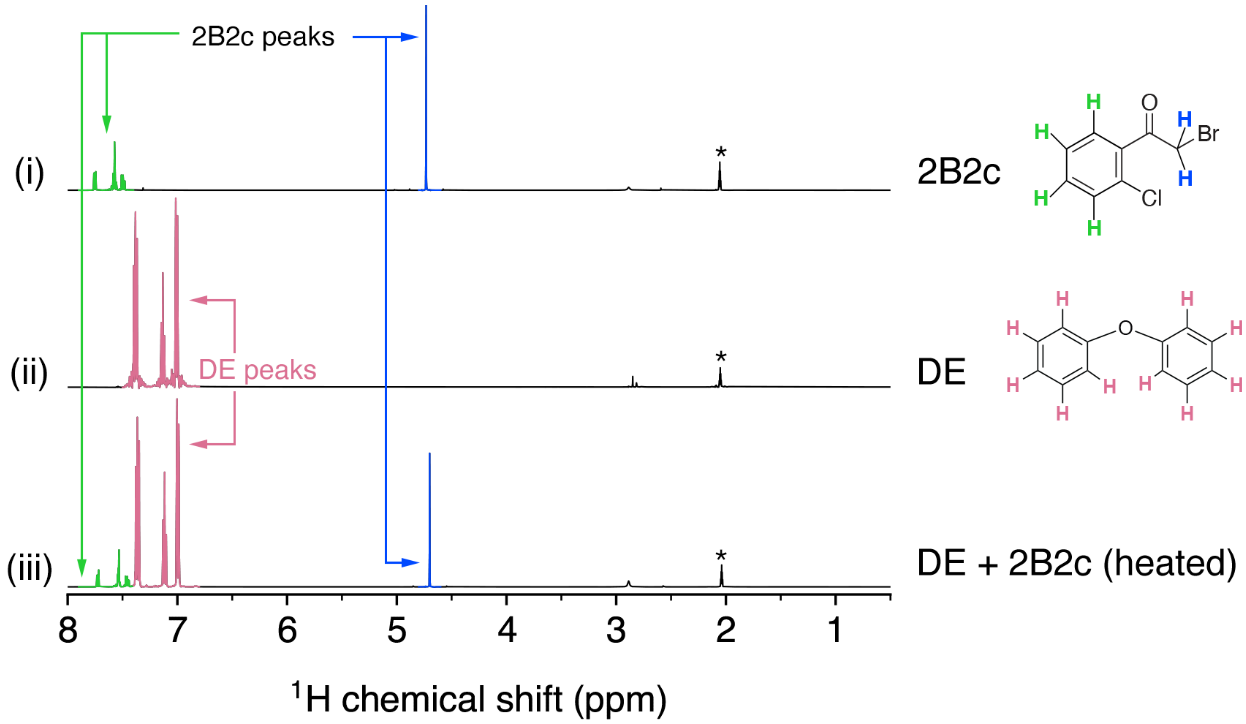

Figure S3: NMR spectra of (i) 2-bromo-2'-chloroacetophenone (2B2c), (ii) diphenyl ether (DE), and (iii) heated DE + 2B2c. This data shows that DE and 2B2c do not react with one another, based on the presence of 2B2c peaks and DE peaks present in the heated DE+2B2c spectrum. There is a small water peak present at ~2.84 ppm on all spectra. The acetone solvent peak is denoted with a (*).

Supplementary Note 3: DE and 2B2c do not react with one another

To understand if diphenyl ether and 2B2c reacted in the ink, they were mixed in the same ratios as used in the ink and heated together under heat activation conditions. As highlighted in **Figure S3**, no visual changes were noticed in the reaction vial and the combined DE + 2B2c ^{1}H NMR spectra, as evidenced by the display of similar peaks in the individual chemicals spectra.

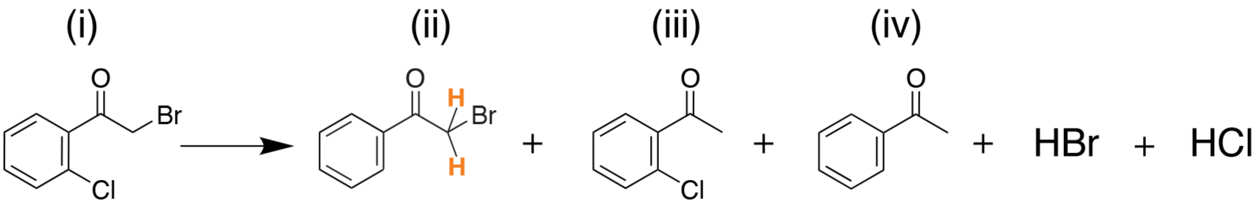

Figure S4: Dehalogenation reaction of 2B2c (reaction 1 in main **Figure 1** showing more detailed products as suggested by NMR spectra, where (i) 2B2c, (ii) 2-bromoacetophenone / phenacyl bromide, (iii) 2'-chloroacetophenone / 1-(2-chlorophenyl)ethanone, (iv) acetophenone, hydrobromic acid and hydrochloric acid.

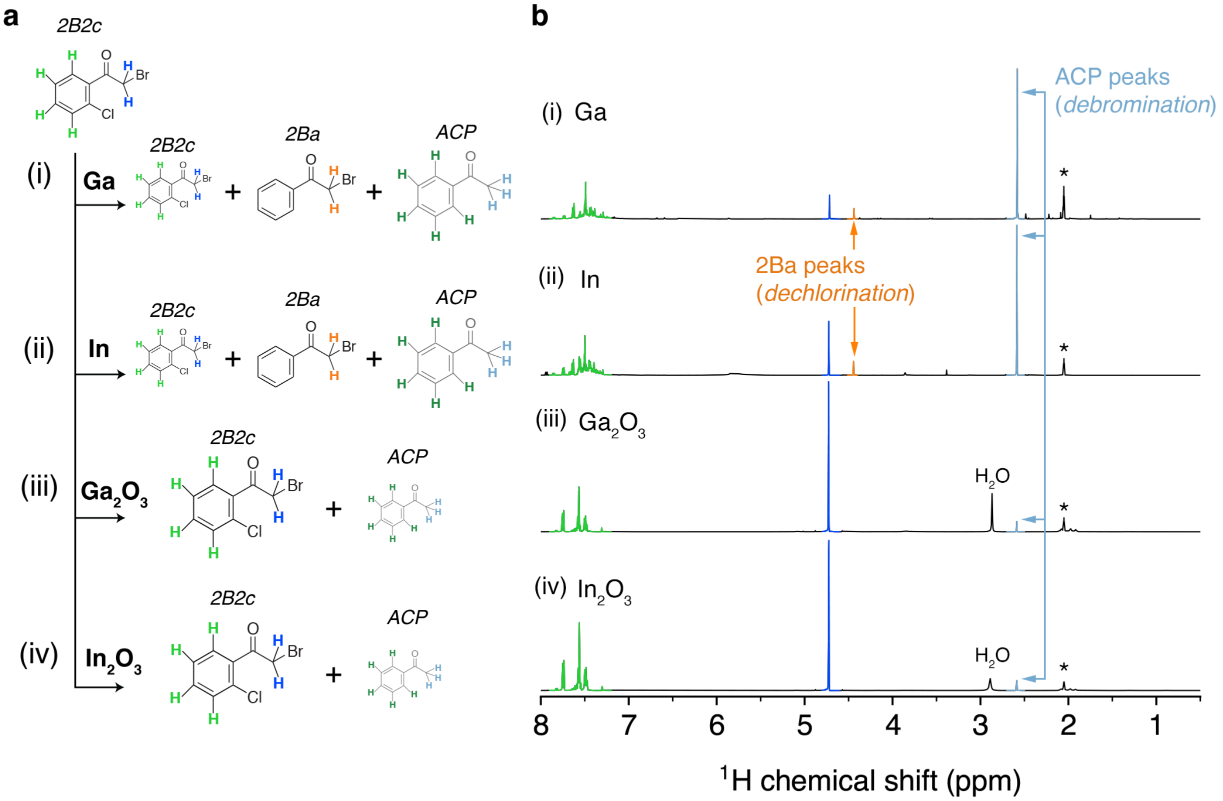

Figure S5: NMR spectra of 2B2c when reacted with (i) gallium (Ga), (ii) indium (In), (iii) gallium oxide (Ga_2O_3), and (iv) indium oxide (In_2O_3). The acetone solvent peak is denoted with a (*). (a) Dehalogenation reaction schemes to accompany NMR results (b) showing the reaction products for each metal catalyzed 2B2c reaction. Molecules are sized according to their relative concentration in the NMR spectra (*e.g.* more acetophenone (ACP). Since a high concentration of dehalogenated product (ACP) as well as dechlorinated product (phenacyl bromide, 2Ba) was found for Ga and In catalyzed reactions, we conclude that dehalogenation strongly favors metals over the metal oxides.

Supplementary Note 4: Gallium and Indium Catalyze 2B2c Dehalogenation

To determine which components in eGaIn were catalyzing dehalogenation, reactivity tests were performed using gallium (99.99%, Thermo Scientific Chemicals), indium powder (-325 mesh 99.99%, Thermo Scientific Chemicals), indium (III) oxide powder (99.99%, Thermo Scientific Chemicals) and gallium (III) oxide powder (99.99%, Thermo Scientific Chemical) using the same sonication, heating, and sampling method as described for eGaIn. The gallium metal was heated to 30°C to melt prior to use. **Figure S5** shows the ^{1}H NMR spectra for these reactivity tests.

The characteristic peaks of dehalogenation appear when the 2B2c is reacted with gallium and indium, indicating that both metals can catalyze the reaction. The metal oxides showed far less catalytic activity, with a large peak at 4.7 ppm signaling the presence of unreacted 2B2c, no de-chlorination peak at 4.5 ppm and a very small debromination peak at 2.6 ppm, which is likely due to either trace metals in the oxides or a very low catylitic activity of the oxides themselves[13].

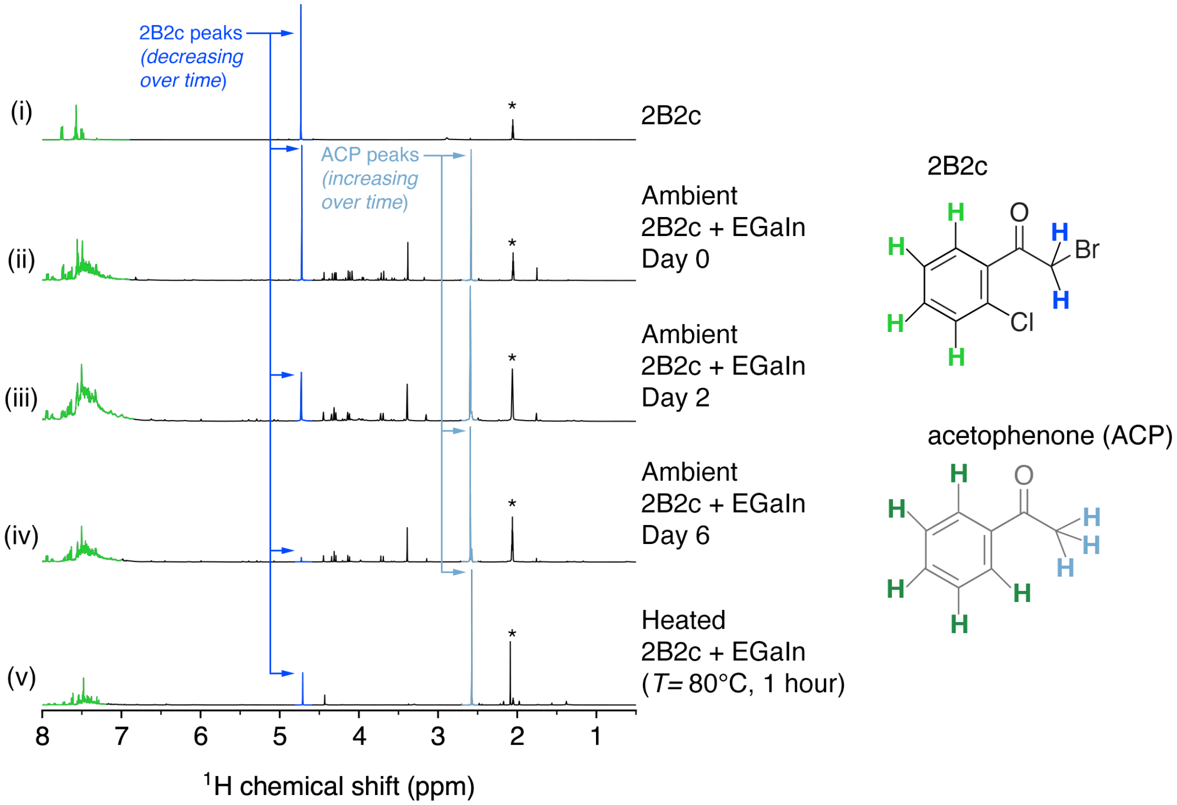

Figure S6: NMR spectra of 2B2c with eGaIn overtime. (i) plain 2B2c (no eGaIn, for comparison), (ii) at ambient with eGaIn on day 0, (iii) at ambient with eGaIn on day 2, and (iv) at ambient with eGaIn on day 6, and (v) heated with eGaIn (T = 80°C, 1 hour). The acetone solvent peak is denoted with a (*). The peaks in (ii), (iii) and (iv) are impurities and described in **SI Note 4**. This data shows the dehalogenation is catalyzed by eGaIn at ambient, but very slowly.

Supplementary Note 5: EGaIn-catalyzed Dehalogenation at Ambient Overtime

Figure S6 shows NMR spectra of plain 2B2c (i), 2B2c bath sonicated with eGaIn and analyzed at different time points [(ii), (iii) and (iv)], and 2B2c sonicated with eGaIn and heated (T = 80°C, 1 hour). The three samples [(ii), (iii) and (iv) above] were prepared as follows: 1 g sample of eGaIn was combined with 75 μL of 2B2c and was bath sonicated for 3 minutes. This mixture was loaded into a syringe and 0.5 g were added to an NMR tube with 600 μL of deuterated acetone. NMR measurements were taken at 0, 2 and 6 days with the same NMR parameters. The sample in (v) was prepared as described in the **Experimental Section**.

The peak at 4.7 ppm (highlighted in faint orange) decreases over the course of the experiment, demonstrating the decrease in unreacted 2B2c, while the debromination peak at 2.5 ppm (highlighted in faint red) increases and levels off. This indicates that the reaction occurs without heating. The peak around 3.38 ppm corresponds to residual ethanol from glassware cleaning.[14] Additional peaks between 1-2 and 3-4.5 ppm are likely residual additives and slip agents from the plastic syringe used to transfer the material.[14] The spectra for plain 2B2c is shown in (i) for comparison. Heat speeds up the reaction ((v) above), as demonstrated by the similar peak heights on Day 2 in the unheated sample.

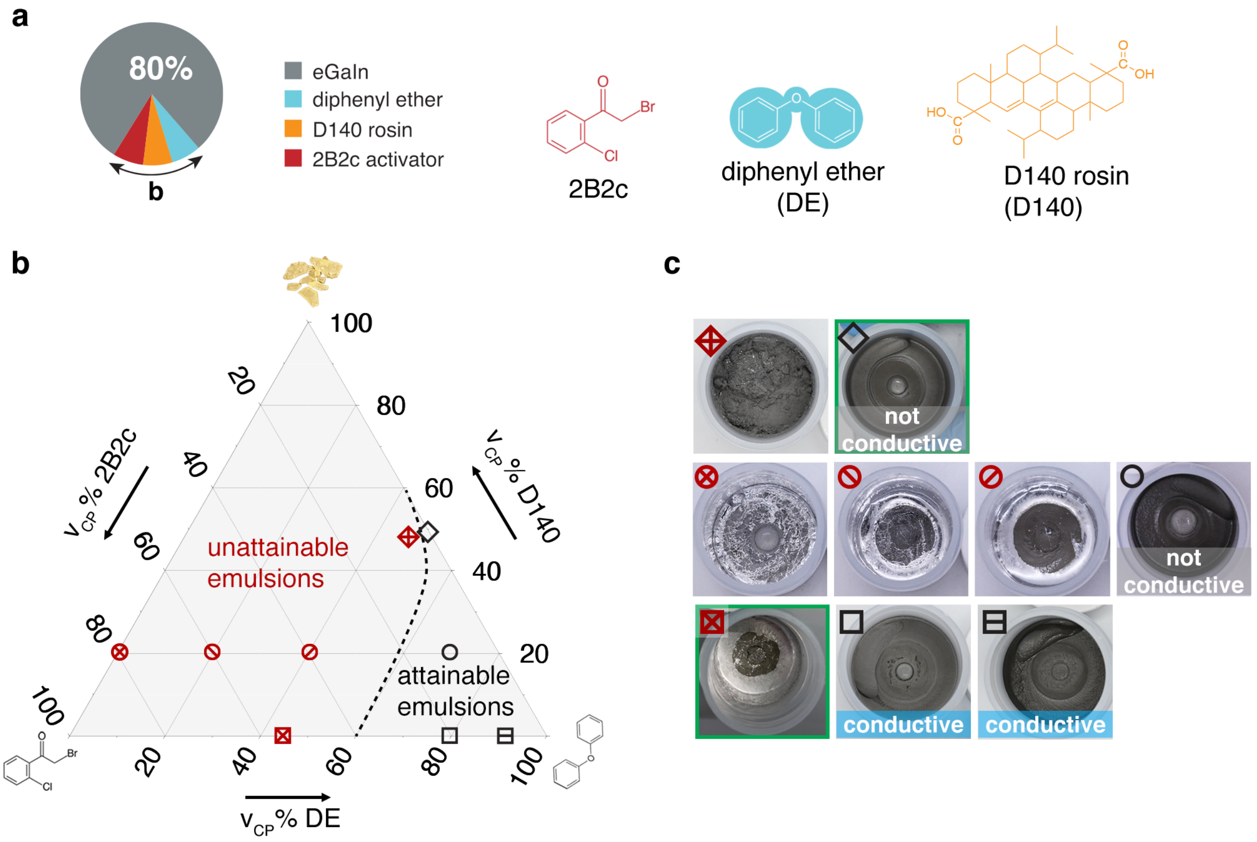

Figure S7: (a) Schematic showing emulsion ink formulation study by fixing the dispersed phase, eGaIn, to ∼80 v/v% while adjusting the continuous phase – diphenyl ether (DE), D140 rosin (D140) and 2-bromo-2'chloroacetophenone (2B2c). (b) Ternary diagram for continuous phase (CP) volume concentrations (v_{CP}) showing regions of attainable (black text and symbols) and unattainable (dark red text and symbols) divided by a line at ∼60 v_{CP}% DE. (c) Top view of mixing cup containing emulsion inks indicated in b). Generally, D140 rosin helps to emulsify eGaIn, but too much D140 yields nonconductive emulsions. Increasing 2B2c for a given D140 rosin concentration yields rigid emulsions with unmixed eGaIn. The highlighted emulsion show that D140 is better at creating homogeneous dispersions of eGaIn since each emulsion was formulated at a similar concentration of D140 or 2B2c.

Table S1: Emulsion continuous phase volume concentrations (v_{CP}) shown in **Figure S7**

	**Volume concentration in continuous phase, v_{CP} (%)**		
**Figure S7 Ternary Diagram Symbol**	**DE**	**D140**	**2B2c**
⊗	0.00	20.00	80.00
⦸	20.00	20.00	60.00
⊘	40.00	20.00	40.00
⊠	45.00	0.00	55.00
⟐	48.75	48.75	2.50
◇	50.00	50.00	0.00
○	70.00	20.00	10.00
□	80.00	0.00	20.00
⊟	90.00	0.00	10.00

Supplementary Note 6: Calculation for Volume Concentration in LMEs

The reported volumetric concentration of components in the emulsion inks are calculated using the following equations:

$$\%v_{ink,eGaIn} = \frac{v_{eGaIn}}{v_{eGaIn} + v_{DE} + v_{D140} + v_{2B2c}} \tag{S17}$$

$$\%v_{ink,DE} = \frac{v_{DE}}{v_{eGaIn} + v_{DE} + v_{D140} + v_{2B2c}} \tag{S18}$$

$$\%v_{ink,D140} = \frac{v_{D140}}{v_{eGaIn} + v_{DE} + v_{D140} + v_{2B2c}} \tag{S19}$$

$$\%v_{ink,2B2c} = \frac{v_{2B2c}}{v_{eGaIn} + v_{DE} + v_{D140} + v_{2B2c}} \tag{S20}$$

To construct the ternary map of continuous phase (CP) components in **Figure S7**, the volume of eGaIn was not included, as shown in the following equations:

$$\%v_{ink,DE} = \frac{v_{DE}}{v_{DE} + v_{D140} + v_{2B2c}} \tag{S21}$$

$$\%v_{ink,D140} = \frac{v_{D140}}{v_{DE} + v_{D140} + v_{2B2c}} \tag{S22}$$

$$\%v_{ink,2B2c} = \frac{v_{2B2c}}{v_{DE} + v_{D140} + v_{2B2c}} \tag{S23}$$

The volume, v_i, of an ink component i, is determined by m_i/ρ_i, where m is the weighed mass of an ink component and ρ is the density of the ink component at $T = 25°$C.

Figure S7 shows the ternary map of the continuous phase of initial ink formulation exploration. Although the D140 rosin and 2B2c could both emulsify eGaIn, the rosin was better than 2B2c at dispersing eGaIn. This can be seen in **Figure S7** where a homogeneous emulsion was achieved when formulated with a continuous phase consisting of 50 v/v% D140 and 50 v/v% DE (black open diamond), while a mixture containing a continuous phase consisting of 55 v/v% 2B2c and 45 v/v% DE (red square with 'X') yielded a nonhomogeneous, two-phase mixture. This may be due to the more reactive interaction that 2B2c has with eGaIn (either through dehalogenation and/or chemical etching of the oxide). Although the D140 rosin was

better at emulsification (likely due to the presence of carboxyl groups which have an affinity for binding with eGaIn[15]) inclusion of 2B2c was necessary for yielding conductive formulations.

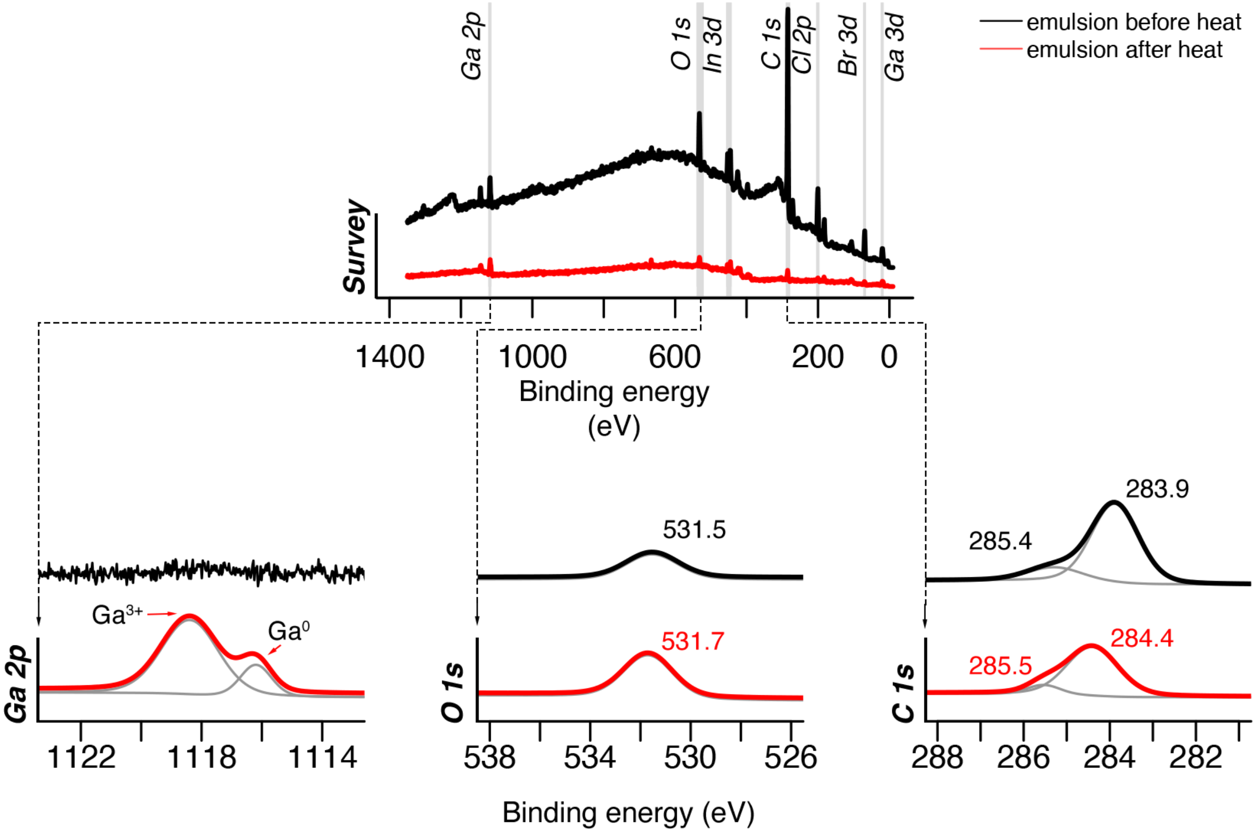

Figure S8: Additional XPS data for emulsion before and after being heated

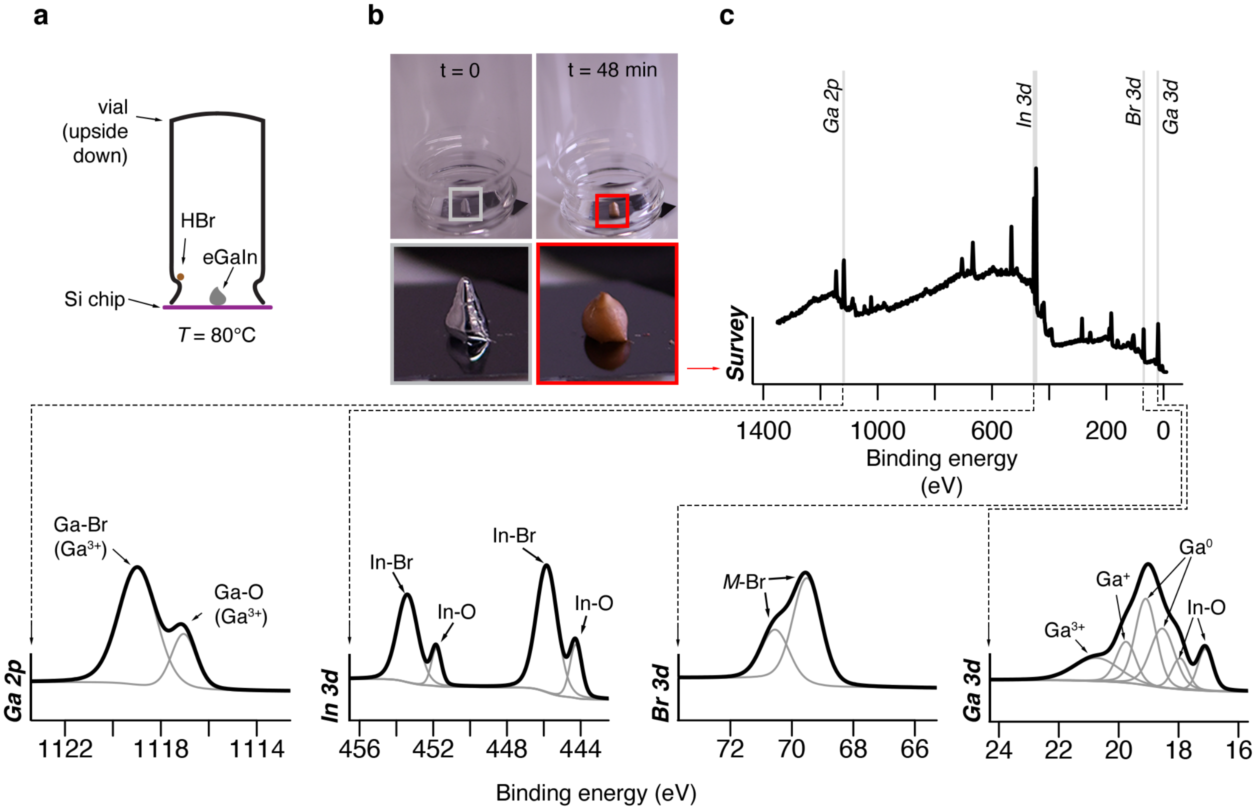

Figure S9: XPS data for eGaIn exposed to HBr at $T = 80°C$

.

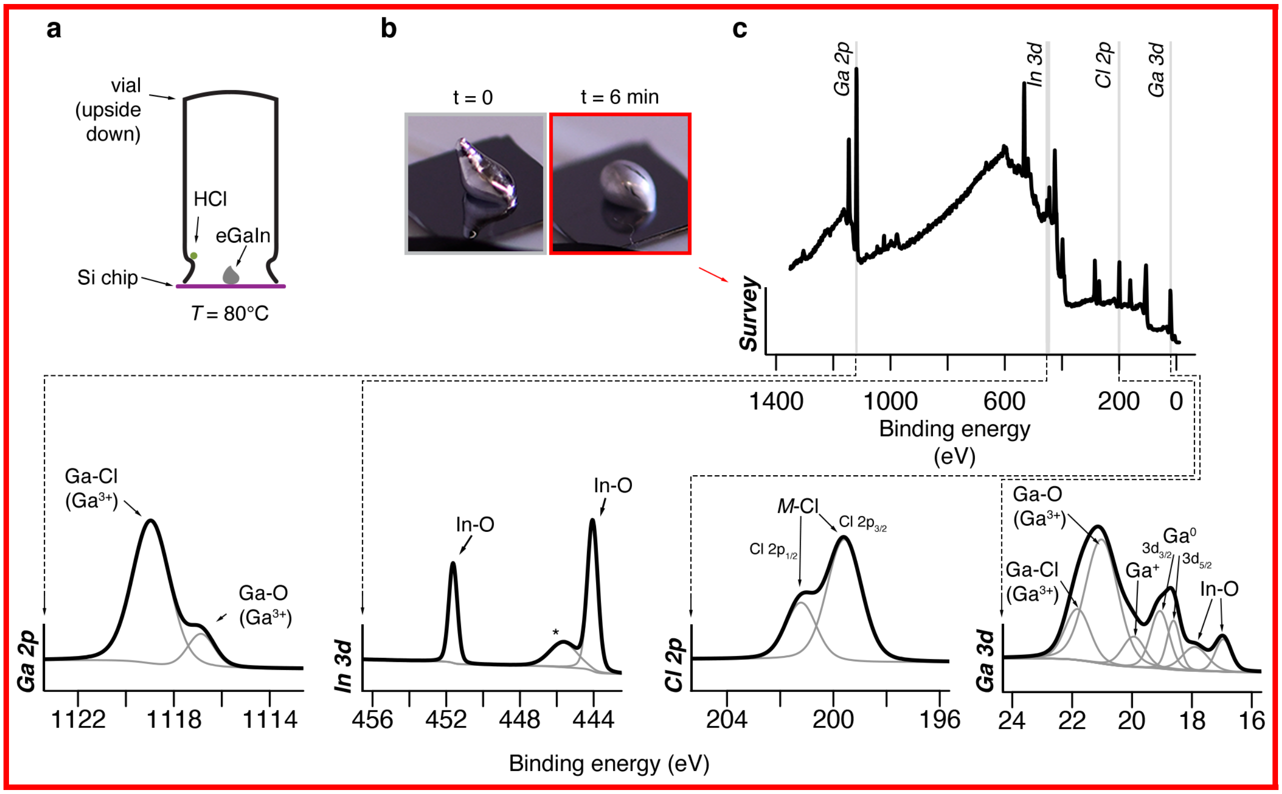

Figure S10: XPS data for eGaIn exposed to HCl at $T = 80°C$.

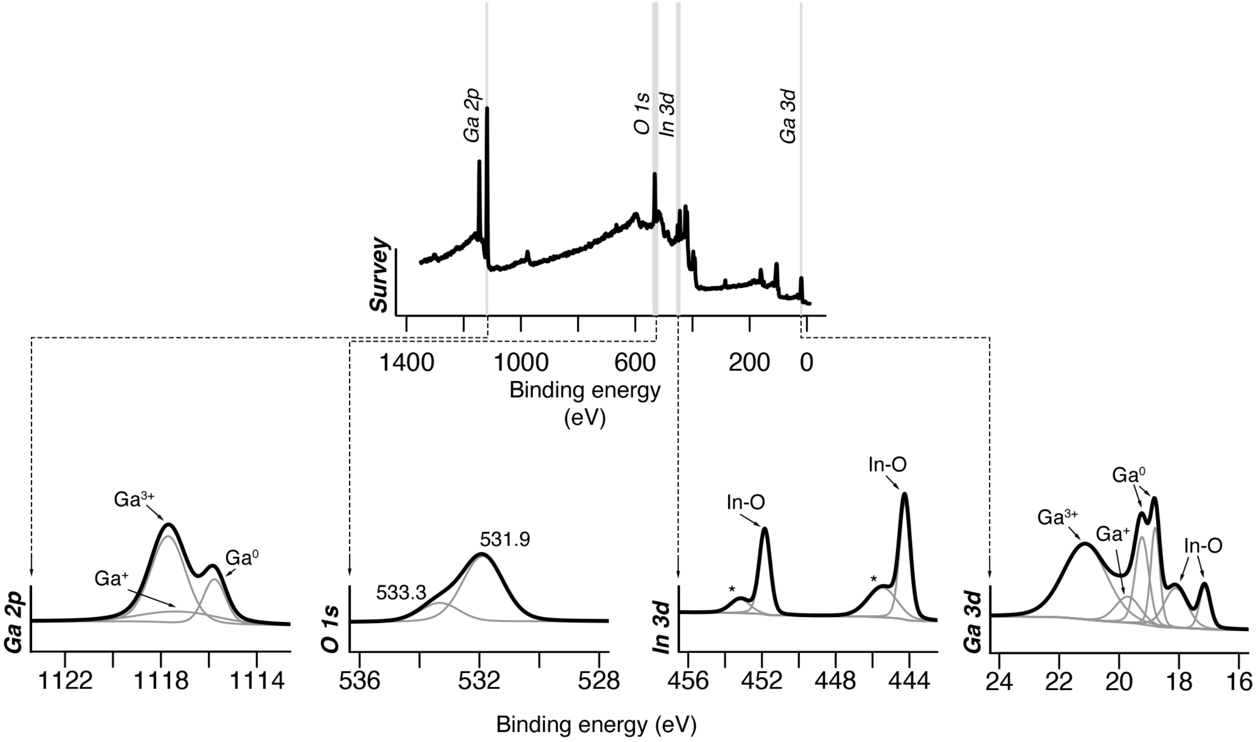

Figure S11: XPS data for eGaIn.

Table S2: Standard enthalpies of formation for reactants and products involved in chemical etching reaction.

**Compound**	ΔH_f° (kJ/mol)	**Ref.**
Ga_2O_3	-1089.1	[16]
GaOOH	-705.3	[17]
In_2O_3	-925.8	[16]
$GaCl_3$	-524.7	[16]
$InCl_3$	-537.2	[16]
$GaBr_3$	-386.6	[16]
$InBr_3$	-428.9	[16]

Table S3: Total mixing times for achieving a homogeneous liquid metal emulsion for 80 v/v% eGaIn formulations listed in main **Figure 2b**.

**v% D140**	**v% 2B2c**	**Mixing times** (min)
0.00%	-	-
	0.05%	10.00
	0.09%	10.00
	0.13%	15.00
	0.25%	15.00
	0.50%	25.00
	1.00%	27.50
	1.50%	35.00
	2.00%	20.00
	2.50%	17.50
	3.00%	30.00
	3.50%	12.50
	4.00%	10.00
	4.50%	15.00
	5.00%	5.00
	5.50%	5.00
0.10%	0.00%	15.00
	0.05%	10.00
	0.09%	12.50
	0.13%	15.00
	0.25%	25.00
	0.50%	22.50
	1.00%	27.50
	1.50%	35.00
	2.00%	30.00
	2.50%	17.50
	3.00%	20.00
	3.50%	15.00
	4.00%	5.00
	4.50%	5.00
	5.00%	5.00
	5.50%	17.50

**v% D140**	**v% 2B2c**	**Mixing times** (min)
0.20%	0.00%	10.00
	0.05%	12.50
	0.09%	12.50
	0.13%	12.50
	0.25%	17.50
	0.50%	15.00
	1.00%	25.00
	1.50%	35.00
	2.00%	45.00
	2.50%	20.00
	3.00%	20.00
	3.50%	12.50
	4.00%	17.50
	4.50%	15.00
	5.00%	10.00
	5.50%	10.00
0.50%	0.00%	10.00
	0.05%	15.00
	0.09%	15.00
	0.13%	17.50
	0.25%	30.00
	0.50%	32.50
	1.00%	20.00
	1.50%	17.50
	2.00%	22.50
	2.50%	15.00
	3.00%	15.00
	3.50%	15.00
	4.00%	7.50
	4.50%	5.00
	5.00%	7.50
	5.50%	5.00

Continued on next page

Table S3 *(Continued from previous page)*

**v% D140**	**v% 2B2c**	**Mixing times** (min)	**v% D140**	**v% 2B2c**	**Mixing times** (min)
0.70%	0.00%	15.00	1.25%	0.00%	20.00
	0.05%	12.50		0.05%	10.00
	0.09%	12.50		0.09%	12.50
	0.13%	15.00		0.13%	22.50
	0.25%	17.50		0.25%	20.00
	0.50%	32.50		0.50%	27.50
	1.00%	15.00		1.00%	40.00
	1.50%	22.50		1.50%	20.00
	2.00%	17.50		2.00%	17.50
	2.50%	15.00		2.50%	17.50
	3.00%	15.00		3.00%	17.50
	3.50%	15.00		3.50%	17.50
	4.00%	12.50		4.00%	10.00
	4.50%	12.50		4.50%	12.50
	5.00%	5.00		5.00%	5.00
	5.50%	5.00		5.50%	5.00
0.90%	0.00%	10.00	2.00%	0.00%	12.50
	0.05%	15.00		0.05%	12.50
	0.09%	17.50		0.09%	17.50
	0.13%	17.50		0.13%	20.00
	0.25%	20.00		0.25%	20.00
	0.50%	20.00		0.50%	32.50
	1.00%	25.00		1.00%	25.00
	1.50%	25.00		1.50%	20.00
	2.00%	20.00		2.00%	22.50
	2.50%	17.50		2.50%	15.00
	3.00%	17.50		3.00%	15.00
	3.50%	15.00		3.50%	15.00
	4.00%	12.50		4.00%	7.50
	4.50%	10.00		4.50%	7.50
	5.00%	7.50		5.00%	5.00
	5.50%	5.00		5.50%	5.00

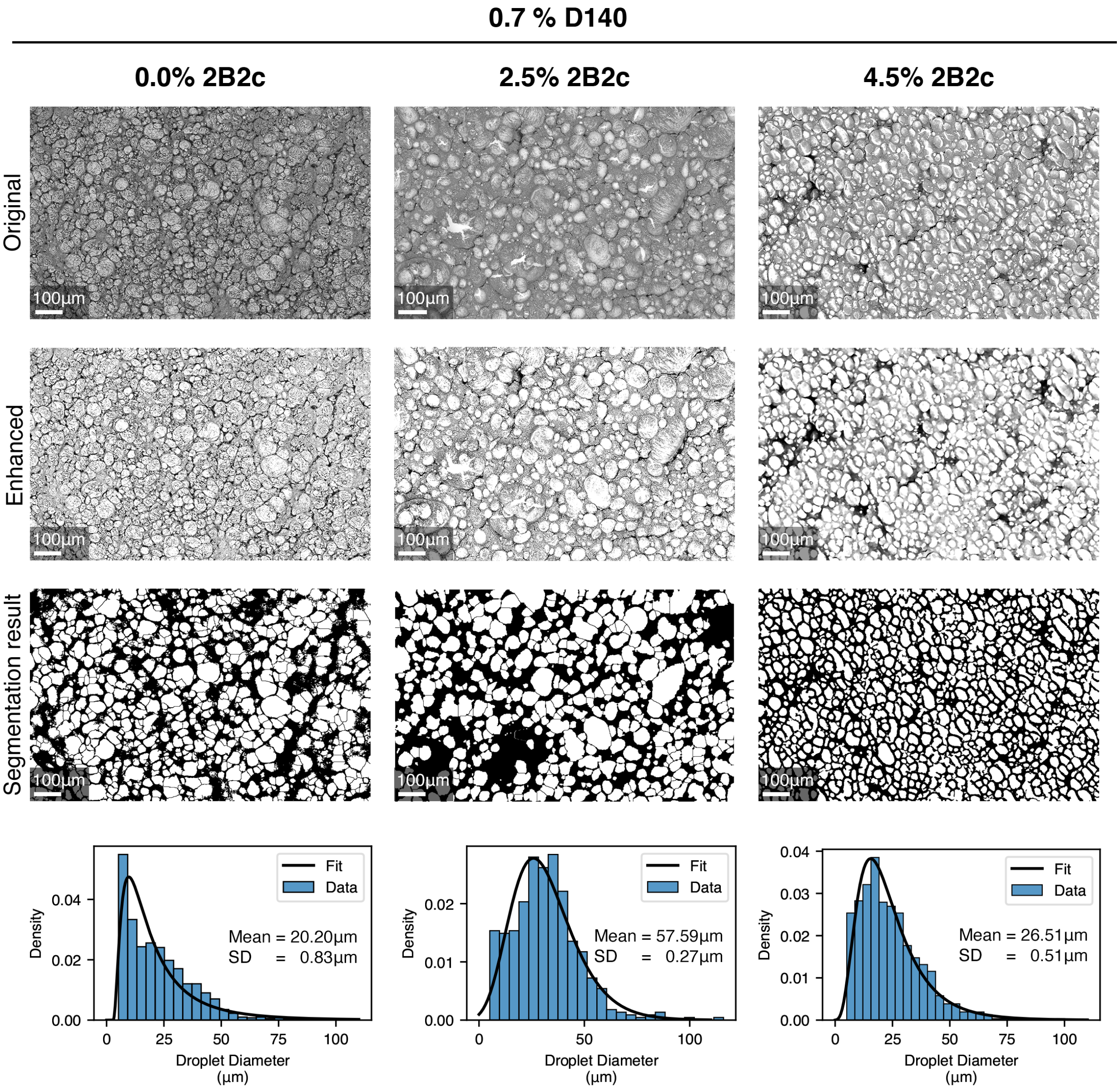

Figure S12: Droplet size distribution of eGaIn droplets in unheated 0.7 v/v% D140 emulsion inks with varying concentration (0, 2.5, and 4.5 v/v% 2B2c) All emulsions were mixed at 3500 RPM for 15 minutes. Droplet diameter was obtained through image analysis using FIJI (ImageJ) as described in the methods section.

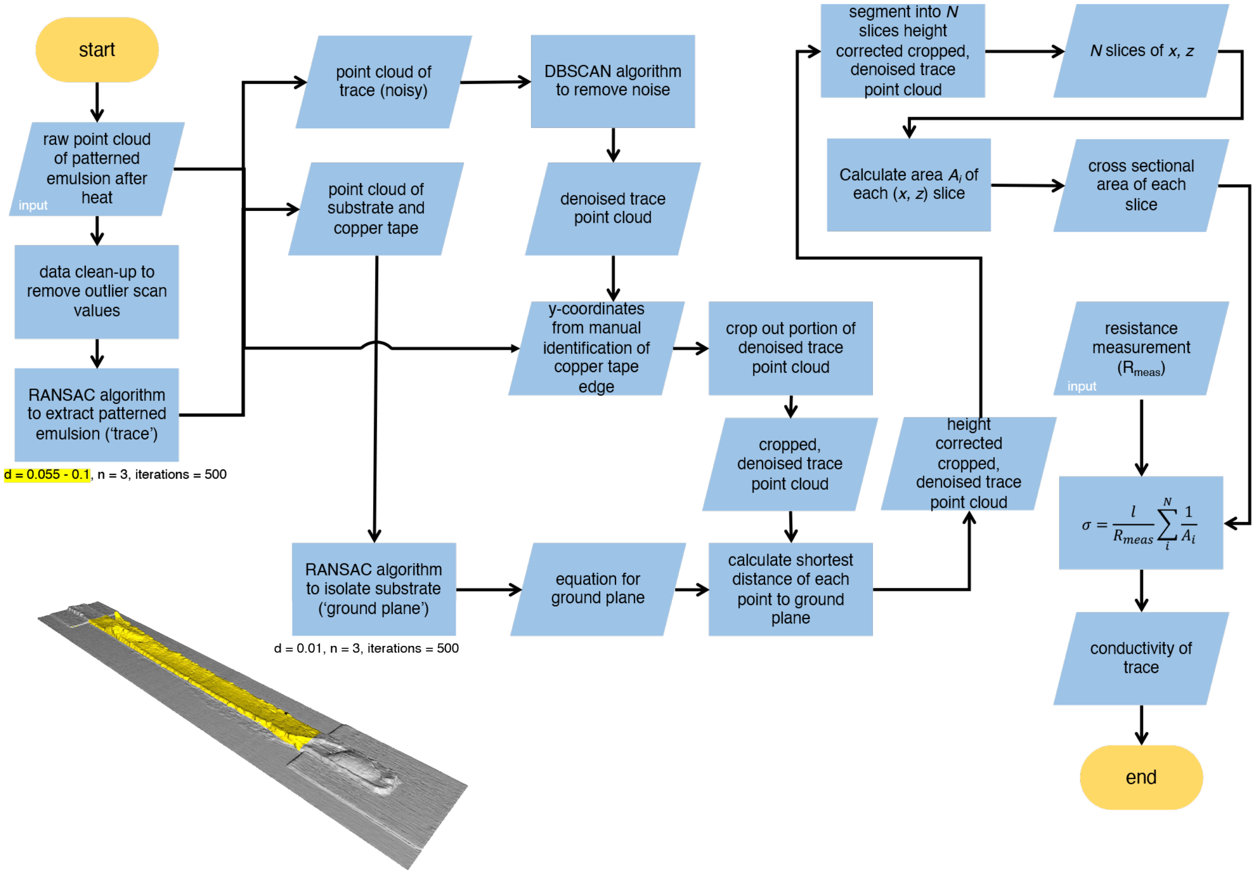

Figure S13: Process flowchart for calculating conductivity of amorphous geometry, which supports the *Ink Conductivity Testing and Analysis* text in the **Experimental Section**. Derivation of the equation used for calculating electrical conductivity (σ) is described in **SI Note 6**.

Supplementary Note 7: Derivation of Discretized Form of the Resistivity Equation

We define the resistance measurement of the trace, R_{meas} as a series of N discrete resistors R_i represented by slices of length l_i of the trace:

$$R_{meas} = \sum_{i=1}^{N} R_i \tag{S24}$$

Plugging in the definition of resistivity (ρ) into R_i, Equation S24 then becomes:

$$R_{meas} = \sum_{i=1}^{N} \frac{\rho_i l_i}{A_i} \tag{S25}$$

If we assume that (ρ) is constant throughout the whole trace if we use the same slice thickness (l), then we can write $\rho_i = \rho$ and $l_i = l$ and Equation S25 becomes:

$$R_{meas} = \rho l \sum_{i=1}^{N} \frac{1}{A_i} \tag{S26}$$

To calculate conductivity (σ), Equation S26 is then rearranged as follows, which is used in the workflow in Figure S13:

$$\sigma = \frac{1}{\rho} = \frac{l}{R_{meas}} \sum_{i=1}^{N} \frac{1}{A_i} \tag{S27}$$

Supplementary Note 8: Estimation of 2B2c Concentration Assuming 100 and 50% Dehalogenation

To estimate the amount of 2B2c needed to react with all the oxides in an 80 v/v% liquid metal emulsion, we consider two cases: (1) 100% dehalogenation, where both bromine (Br) and chlorine (Cl) are completely removed, and (2) 50% dehalogenation (100% debromination) where only Br is removed. We assume an emulsion with liquid metal (LM) droplets as spheres (**Figure S14**) and a 3 nm oxide.[18]

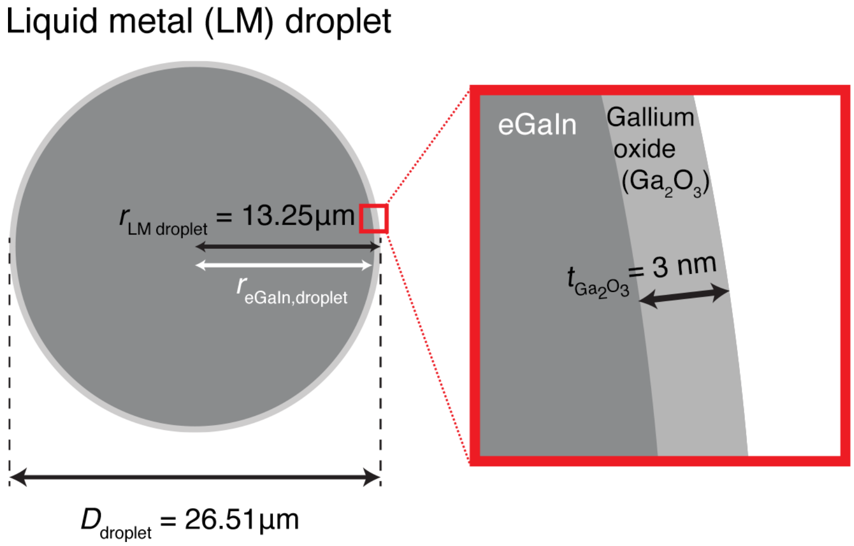

Figure S14: Dimensions of ideal eGaIn droplet in 80 v/v% liquid metal emulsion. The diameter of the droplet (D_{droplet}) was taken from mean droplet diameter calculated from **Figure S12**.

For each case, the amount of required 2B2c is calculated stoichiometrically by first determining the total number of moles of gallium oxide ($N_{\text{Ga}_2\text{O}_3,\text{tot}}$) through the following equations assuming a total volume (V_{tot}) of 1 mL:

$$N_{\text{Ga}_2\text{O}_3,\text{ tot}} = \frac{m_{\text{Ga}_2\text{O}_3,\text{ tot}}}{M_{\text{Ga}_2\text{O}_3}} \tag{S28}$$

$$m_{\text{Ga}_2\text{O}_3,\text{tot}} = n_{\text{droplet}} \times m_{\text{Ga}_2\text{O}_3,\text{droplet}} \tag{S29}$$

$$m_{\text{Ga}_2\text{O}_3,\text{droplet}} = \rho_{\text{Ga}_2\text{O}_3} \times V_{\text{Ga}_2\text{O}_3,\text{droplet}} \tag{S30}$$

$$\begin{aligned} V_{\text{Ga}_2\text{O}_3,\text{droplet}} &= V_{\text{LM droplet}} - V_{\text{eGaIn,droplet}} \\ &= \frac{4}{3}\pi r^3_{\text{LM droplet}} - \frac{4}{3}\pi r^3_{\text{eGaIn,droplet}} \end{aligned} \tag{S31}$$

$$r_{\text{eGaIn,droplet}} = r_{\text{LM droplet}} - t_{\text{Ga}_2\text{O}_3} \tag{S32}$$

$$n_{\text{droplet}} = \frac{V_{\text{LM}}}{V_{\text{LM droplet}}} = \frac{0.8 \times V_{\text{tot}}}{\frac{4}{3}\pi r^3_{\text{LM droplet}}} \tag{S33}$$

where the variables and values for **Equations S28 - S33** are listed in **Table S4** and the total number of moles of Ga_2O_3 to react is 1.87×10^{-5} moles.

Table S4: Variables and values used in **Equations S28 - S33**. Italicized values are constants used in calculations. Calculated values assume a total emulsion volume of 1 cm^3.

Variable	Description	Value	Units
$N_{Ga_2O_3,tot}$	Total moles of Ga_2O_3	1.87×10^{-5}	mol
$m_{Ga_2O_3,\ tot}$	Total mass of Ga_2O_3	3.50×10^{-3}	g
$M_{Ga_2O_3}$	Molar mass of Ga_2O_3	*187.44*	g mol^{-1}
$n_{droplet}$	Number of LM droplets in emulsion	8.20×10^{7}	droplets
V_{LM}	Volume of liquid metal in total emulsion in emulsion	0.8	cm^3
V_{tot}	Total emulsion volume	1.0	cm^3
$m_{Ga_2O_3,droplet}$	Mass of Ga_2O_3 in a droplet of eGaIn, assuming $D_{droplet} = 26.51\mu m$	4.26×10^{-11}	g $droplet^{-1}$
$\rho_{Ga_2O_3}$	Density of Ga_2O_3	*6.44*	g cm^{-3}
$V_{Ga_2O_3,droplet}$	Volume of Ga_2O_3 in one droplet	6.62×10^{-12}	cm^3 $droplet^{-1}$
$V_{LM\ droplet}$	Volume of liquid metal droplet (eGaIn + oxide)	9.76×10^{-9}	cm^3 $droplet^{-1}$
$V_{eGaIn,droplet}$	Volume of eGaIn in one droplet (no oxide)	9.75×10^{-9}	cm^3 $droplet^{-1}$
$r_{LM\ droplet}$	Radius of liquid metal droplet (eGaIn + oxide)	13.255	μm
$r_{eGaIn,droplet}$	Radius of eGaIn in liquid metal droplet (without oxide)	13.252	μm
$t_{Ga_2O_3}$	Thickness of oxide	*3*[18]	nm

Case 1: 100% Dehalogenation

In 100% dehalogenation, we assume the following dehalogenation and oxide etching reactions:

$$ClC_6H_4COCH_2Br + 2\,H_2 \rightarrow C_6H_4COCH_2 + HBr + HCl \tag{S34}$$

$$Ga_2O_3 + 3\,HBr + 3\,HCl \rightarrow GaBr_3 + GaCl_3 + 3\,H_2O \tag{S35}$$

The molar ratio of 2B2c:Ga_2O_3 can be seen from **Equations S34** and **S35** above to be 3 moles 2B2c : 1 mole Ga_2O_3. Thus, the estimated 2B2c concentration ($v/v_{2B2c,100}\%$) to remove all oxides is:

$$v/v_{2B2c,100}\% = \frac{V_{2B2c}}{V_{tot}} = \frac{N_{2B2c} \times M_{2B2c}}{\rho_{2B2c}} \cdot \frac{1}{V_{tot}} = \frac{3 \times N_{Ga_2O_3,tot} \times M_{2B2c}}{\rho_{2B2c}} \cdot \frac{1}{V_{tot}} = 0.82\% \tag{S36}$$

where N_{2B2c} is the calculated number of moles of 2B2c to react, M_{2B2c} is the molar mass of 2B2c (233.49 g mol^{-1}), and ρ_{2B2c} is the density of 2B2c (1.602 g cm^{-3}).

Case 2: 50% Dehalogenation (100% Debromination)

In 50% dehalogenation, we assume the following dehalogenation and oxide etching reactions:

$$ClC_6H_4COCH_2Br + 1.5\,H_2 \rightarrow ClC_6H_4COCH_2 + HBr \tag{S37}$$

$$Ga_2O_3 + 6\,HBr + 3\,HCl \rightarrow 2\,GaBr_3 + 3\,H_2O \tag{S38}$$

The molar ratio of 2B2c:Ga_2O_3 can be seen from **Equations S37** and **S38** above to be 6 moles 2B2c : 1 mole Ga_2O_3. Thus, the estimated 2B2c concentration ($v/v_{2B2c,50}\%$) to remove all oxides is:

$$v/v_{2B2c,50}\% = \frac{V_{2B2c}}{V_{tot}} = \frac{N_{2B2c} \times M_{2B2c}}{\rho_{2B2c}} \cdot \frac{1}{V_{tot}} = \frac{6 \times N_{Ga_2O_3,tot} \times M_{2B2c}}{\rho_{2B2c}} \cdot \frac{1}{V_{tot}} = 1.63\% \tag{S39}$$

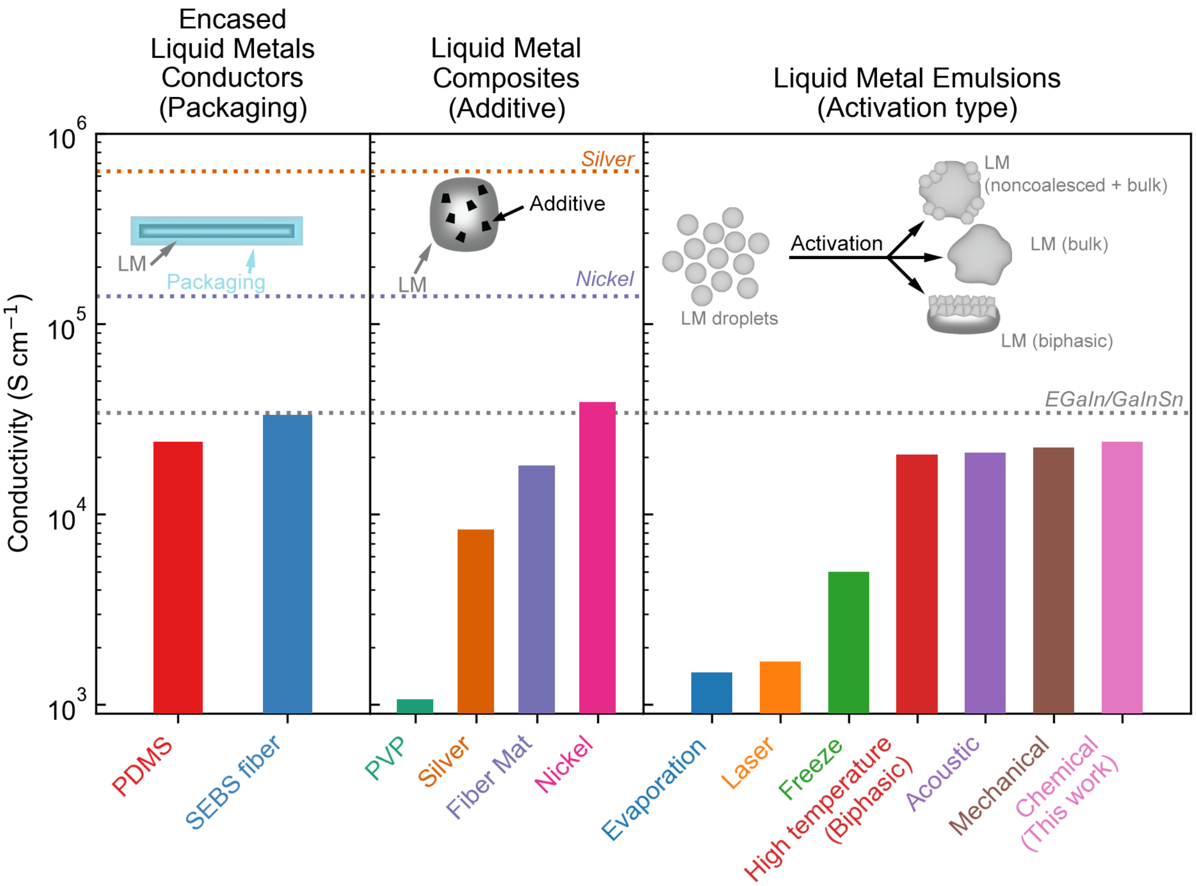

Figure S15: Comparison of LME to other researched liquid metal conductors (highest reported conductivity plotted). Dotted lines indicate conductivity values for the stated bulk metal. Schematics in each subplot show how each liquid metal conductor is classified.

Table S5: Electrical conductivity (S/cm) of liquid metal-based conductors shown in **Figure S15**

**ID**	**Type**	**Electrical Conductivity** (S cm^{-1})	**Reference**
PDMS	Encased	2.41×10^4	[19]
SEBS fiber	Encased	3.33×10^4	[20]
PVP	Composite	1.07×10^3	[21]
Silver	Composite	8.33×10^3	[22]
Fiber Mat	Composite	1.80×10^4	[23]
Nickel	Composite	3.90×10^4	[24]
Evaporation	Emulsion	1.48×10^3	[15], [25]
Laser	Emulsion	1.68×10^3	[26]
Freeze	Emulsion	5.00×10^3	[27]
High temperature (biphasic)	Emulsion	2.06×10^4	[28]
Acoustic	Emulsion	2.10×10^4	[29]
Mechanical	Emulsion	2.25×10^4	[30],[31],[32],[33],[34],[35],[27],
Chemical	Emulsion	2.40×10^4	This work

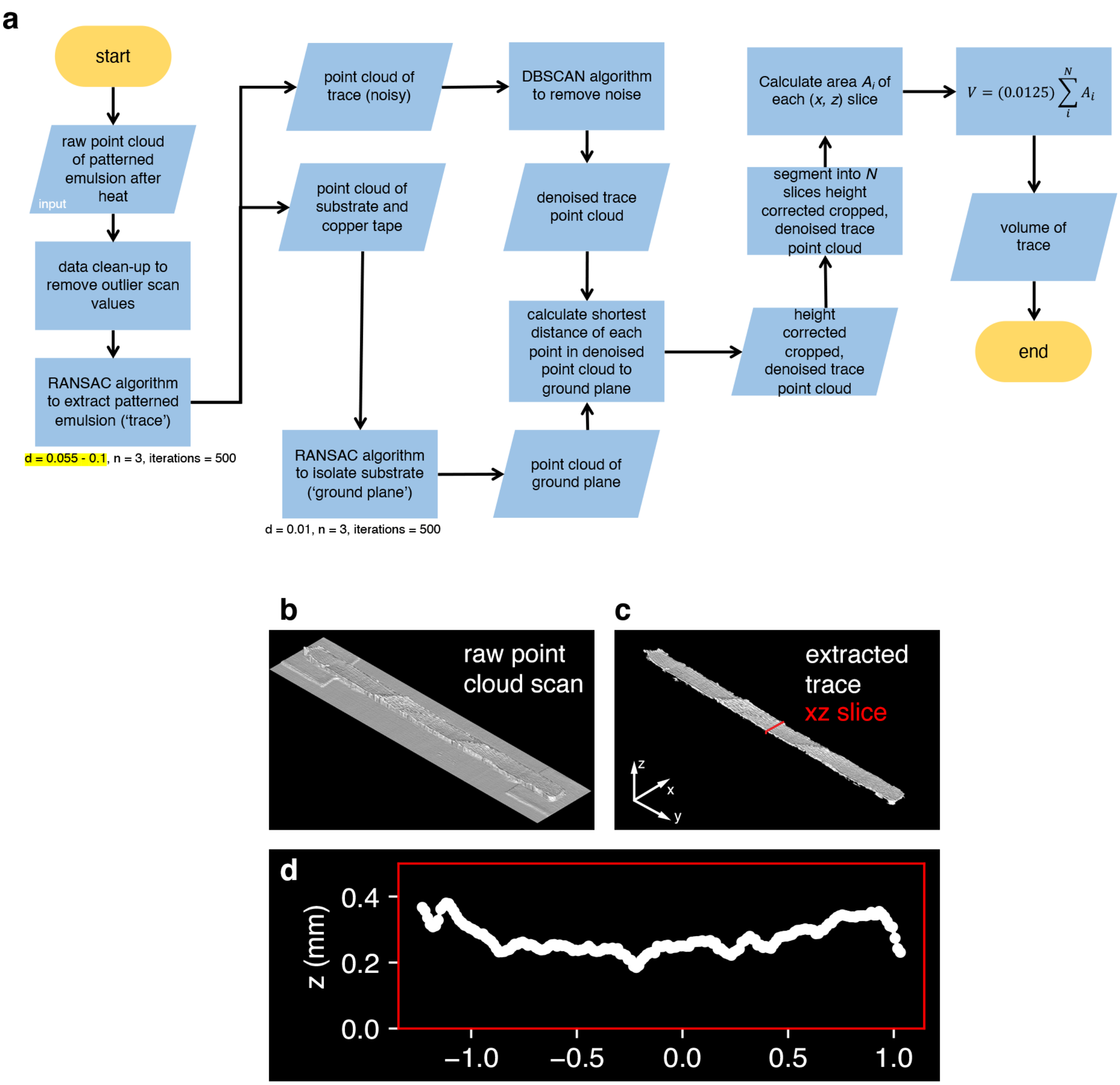

Figure S16: Shrinkage calculation variables for emulsion ink shown in main **Figure 2**. (a) Code workflow for extracting patterned emulsion trace from raw point cloud which utilizes RANSAC[36] and DBSCAN[37] algorithms for cleaning 3D scans. (b) Point cloud of raw scan. (c) Denoised trace point cloud showing slice of points used for calculating area. The denoised trace point cloud is segmented in slices spaced 12.5 μm apart (the resolution of the scan). (d) x and z coordinates of slice shown in (c).

Supplementary Note 9: Calculation of LME Shrinkage

Shrinkage was calculated by the following equations:

$$S = \frac{V_{\text{before heat}} - V_{\text{after heat}}}{V_{\text{before heat}}} \times 100\% \tag{S40}$$

$$S = \frac{(\sum_i^N A_i)_{\text{before heat}} - (\sum_i^N A_i)_{\text{after heat}}}{(\sum_i^N A_i)_{\text{before heat}}} \times 100\% \tag{S41}$$

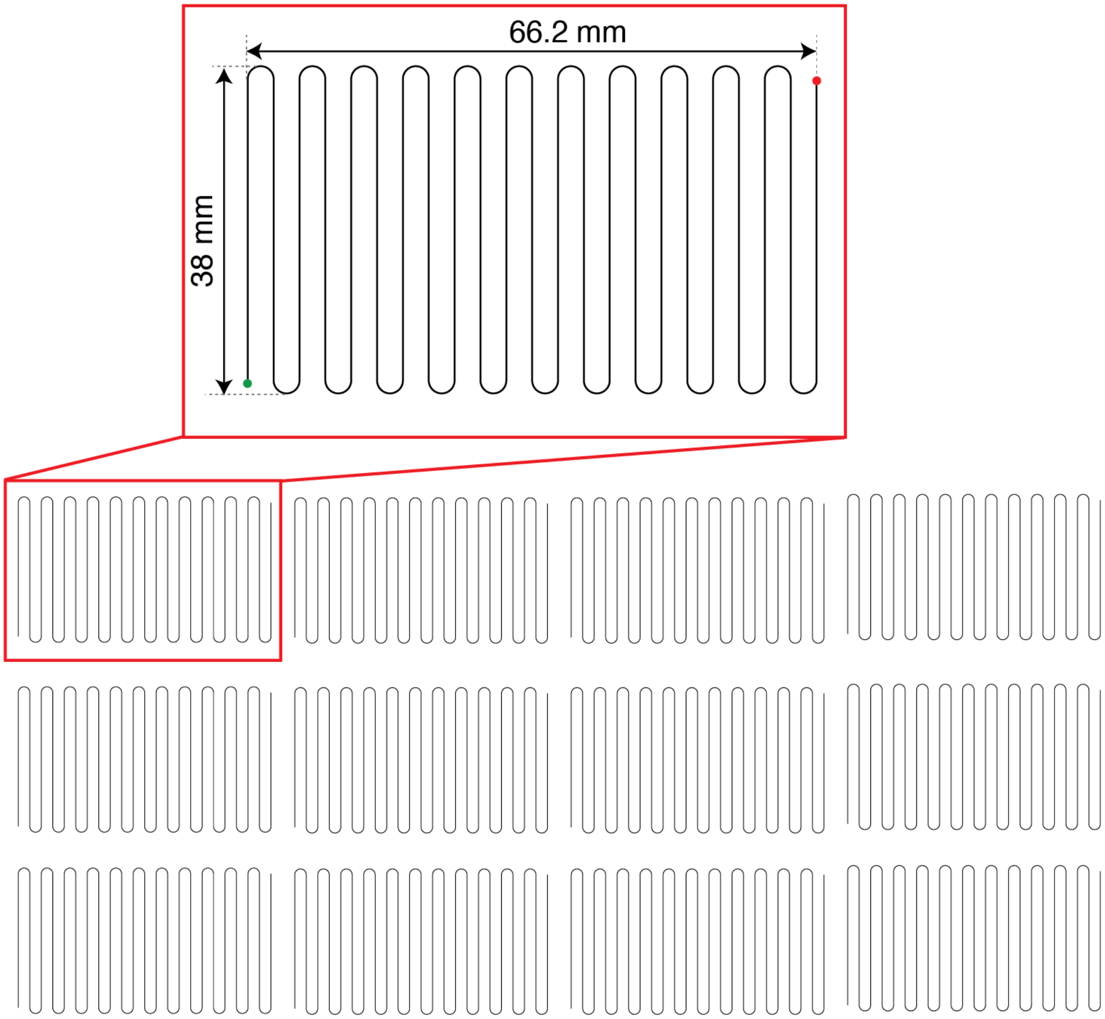

Figure S17: Schematic showing print path for testing the extrudability of liquid metal emulsion formulations. The serpentine pattern in the red outlined inset shows the repeated serpentine pattern used to extrude each formulation, with the start and end of print denoted by the green and red circle, respectively.

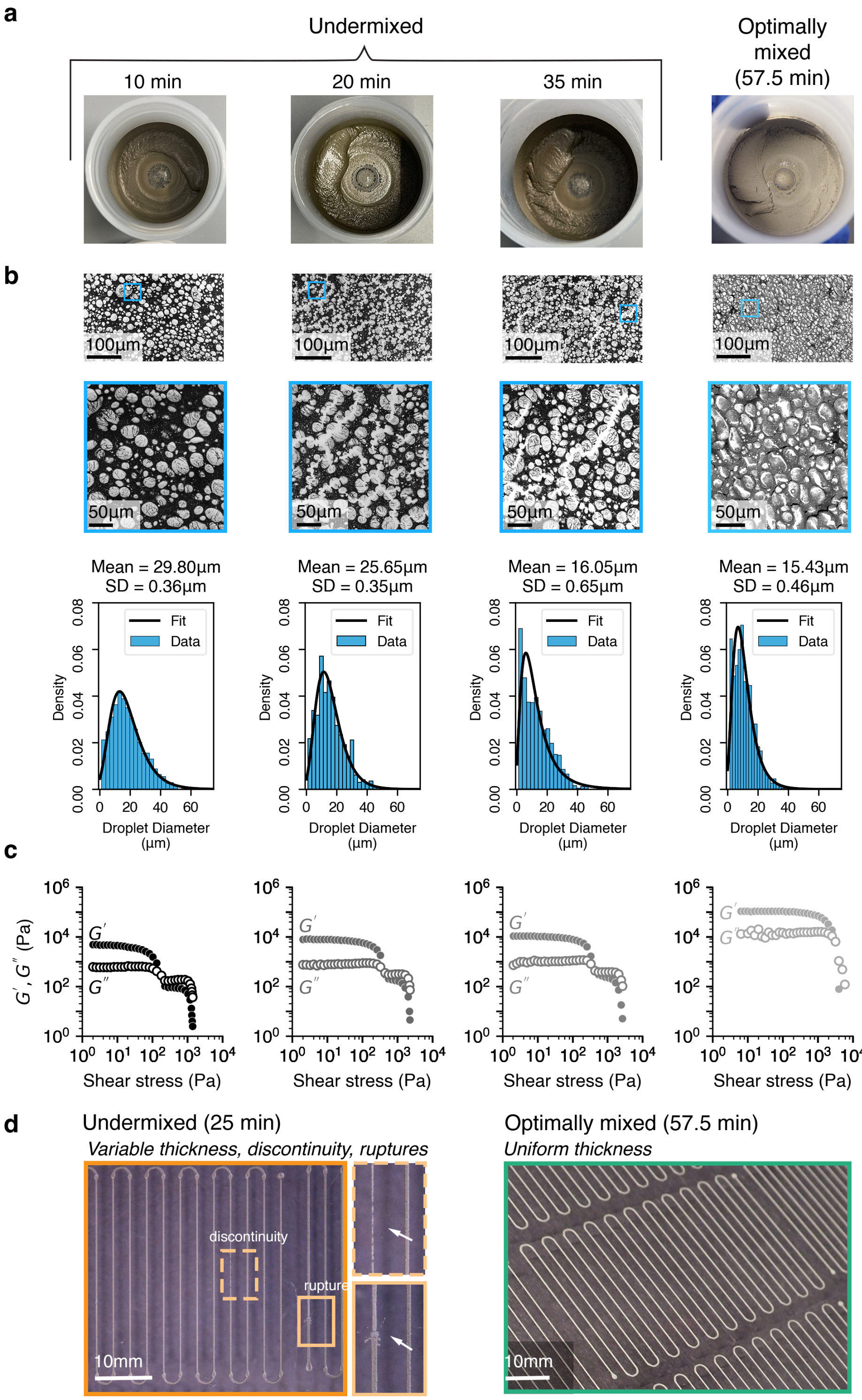

Figure S18: Effect of droplet size on rheology and printability. (a) Optimal LME formulation (80 v/v% eGaIn, 0.7 v/v% D140, 4.5 v/v%2B2c) at different mix times (b) SEM images of LMEs in (a) and the corresponding droplet size distribution. (c) Oscillation amplitude data for LMEs in (a) and (b). (d) Printability results of undermixed and optimally mixed LME.

Table S6: Calculated values for droplet size log-normal distribution data and oscillation amplitude data from **Figure S18**, for optimum LME (80 v/v% eGaIn, 0.7 v/v% D140, 4.5 v/v% 2B2c) at different mix times.

	**10 min mixed**	**20 min mixed**	**35 min mixed**	**Optimally mixed (57.5 min)**
Median, m (μm)	27.88	24.13	13.02	13.89
Mean, μ (μm)	29.80	25.65	16.05	15.43
Standard deviation (SD)	0.36	0.35	0.64	0.46
G' (Pa)	4.7×10^3	7.9×10^3	1.0×10^4	1.0×10^5

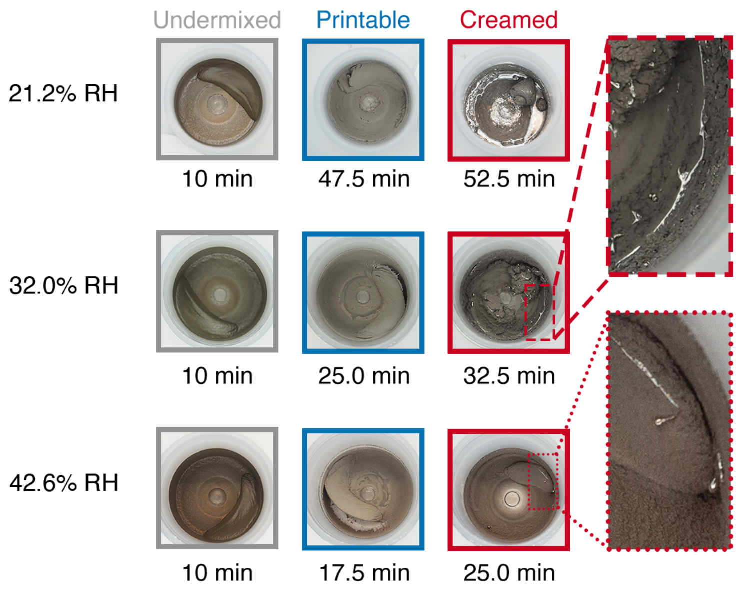

Figure S19: Effect of relative humidity (RH) on mixing (3500 RPM) time of printable liquid metal emulsion (LME) formulation (∼80v/v% eGaIn, ∼0.7v/v% D140, ∼4.5v/v% 2B2c). At 21.2% RH, the LME is undermixed when mixed for 10 minutes (grey outline). When mixed for 47.5 minutes, an emulsion with printable consistency is achieved (blue outline). When mixed beyond the time needed to achieve printable consistency (52.5 minutes), the emulsion creams (red outline). Mixing past the onset of a printable consistency renders an emulsion that is no longer usable. At higher %RH (middle and bottom row, 32.0 and 42.6% RH, respectively), mixing times for the same formulation yields shorter mix times. Furthermore, creaming still occurs when the emulsion is further mixed past a printable consistency.

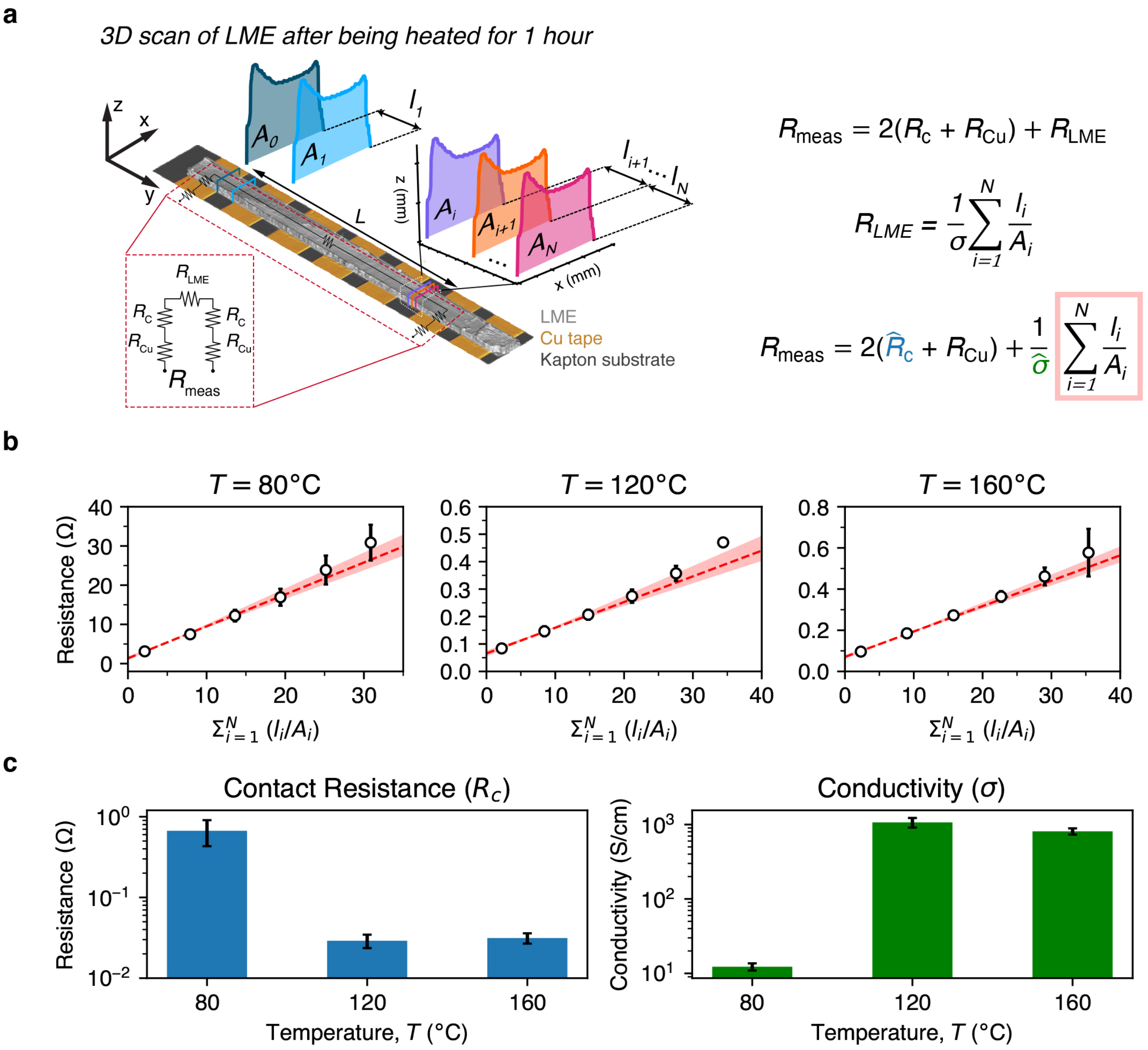

Figure S20: Contact Resistance of Emulsion Ink at Higher Temperatures (80, 120, and 160°C). (a) Schematic of contact resistance experiment using copper (Cu) tapes and transmission line equation,([38],[39]) utilizing our derived definition (**Equation S27**) of calculating conductivity from the volume of an amorphous geometry. (b) Mean resistance values obtained at varying volumes, L (circles) for a particular temperature, T(°C). Error bars denote standard error of the mean. The dashed red line in each subplot is the line fit to the transmission line equation. The transparent red region denote the model fit using the calculated parameters along with their errors. (c) Calculated values of contact resistance (left) and conductivity (right) from the model fit to the data, values listed in **Table S7**.

Table S7: Measured Copper Resistance (R_{Cu}), Calculated Contact Resistance (R_c) and Conductivity (σ) from **Figure S20**

**Temperature** (°C)	$R_{\mathbf{Cu}}$ (Ω)	R_c (Ω)	σ (S/cm)
80	0.0038	0.669 ± 0.238	$1.228 \pm 0.135 \times 10^1$
120	0.0037	0.029 ± 0.005	$1.068 \pm 0.158 \times 10^3$
160	0.0039	0.031 ± 0.004	$8.106 \pm 0.775 \times 10^2$

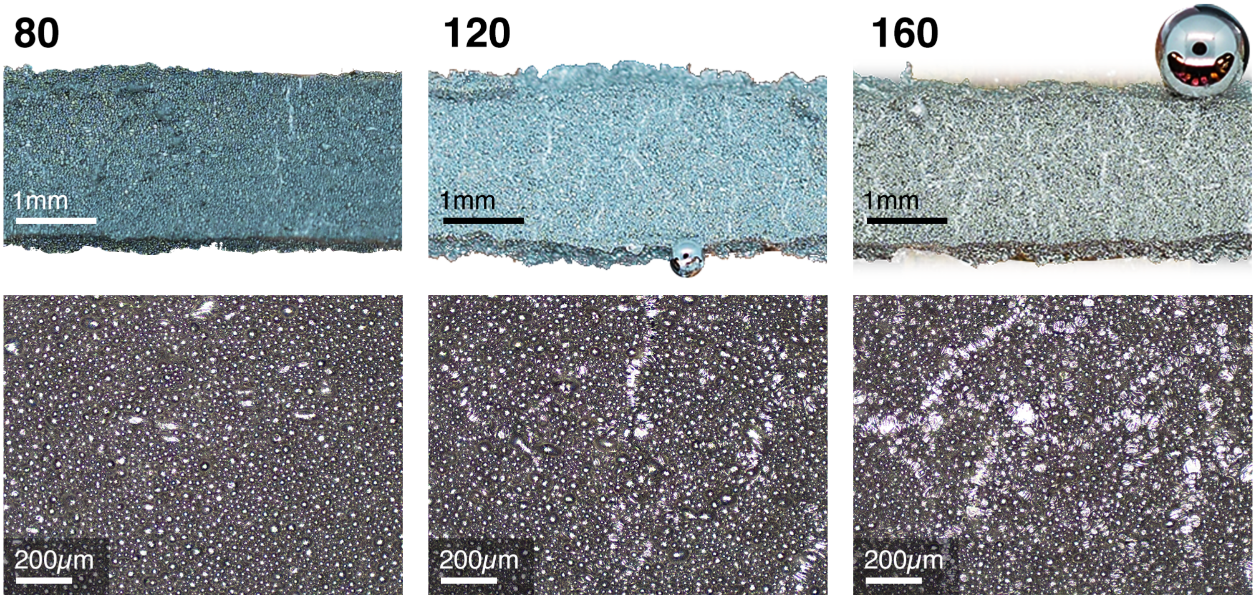

Figure S21: Pictures of Liquid Metal Emulsion Ink at Higher Temperatures (80, 120, and 160°C) during contact resistance measurements. Top row: Macroscale image of emulsion ink traces. Bottom row: Microscope images of emulsion inks. In each row, it can be seen that the degree of coalescence (shown by increase of emerging cracks of liquid metal) increases with temperature.

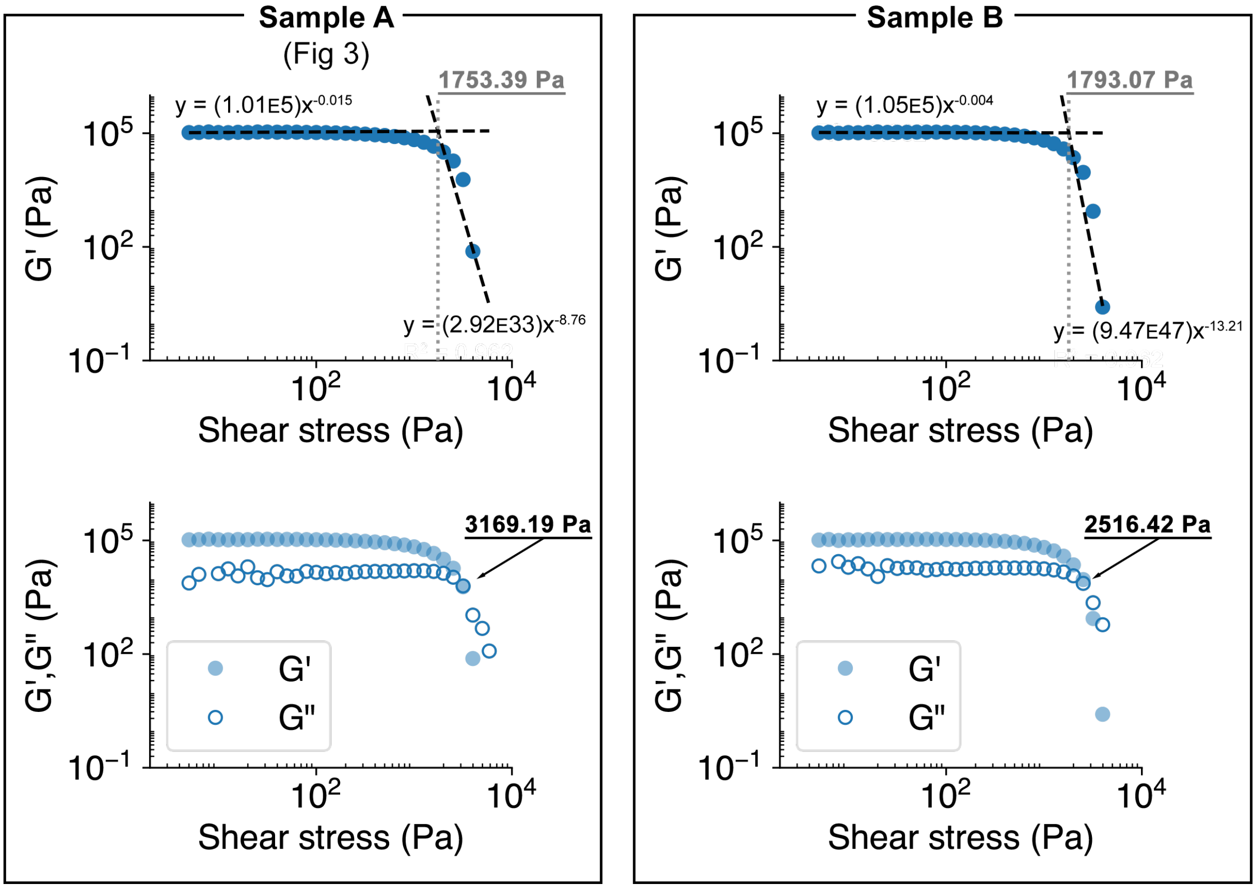

Figure S22: Yield stress calculation of liquid metal emulsion (same formulation, two different samples) using two methods to determine yield stress. Top figure in each inset: Using G' data and finding the point at which the horizontal line through linear G' data intersects with the power law line fit to the G' data where the G' drops off at a steep rate. Bottom figure in each inset: Identifying shear stress at which $G' \approx G''$.

Sample A
(Fig 3)

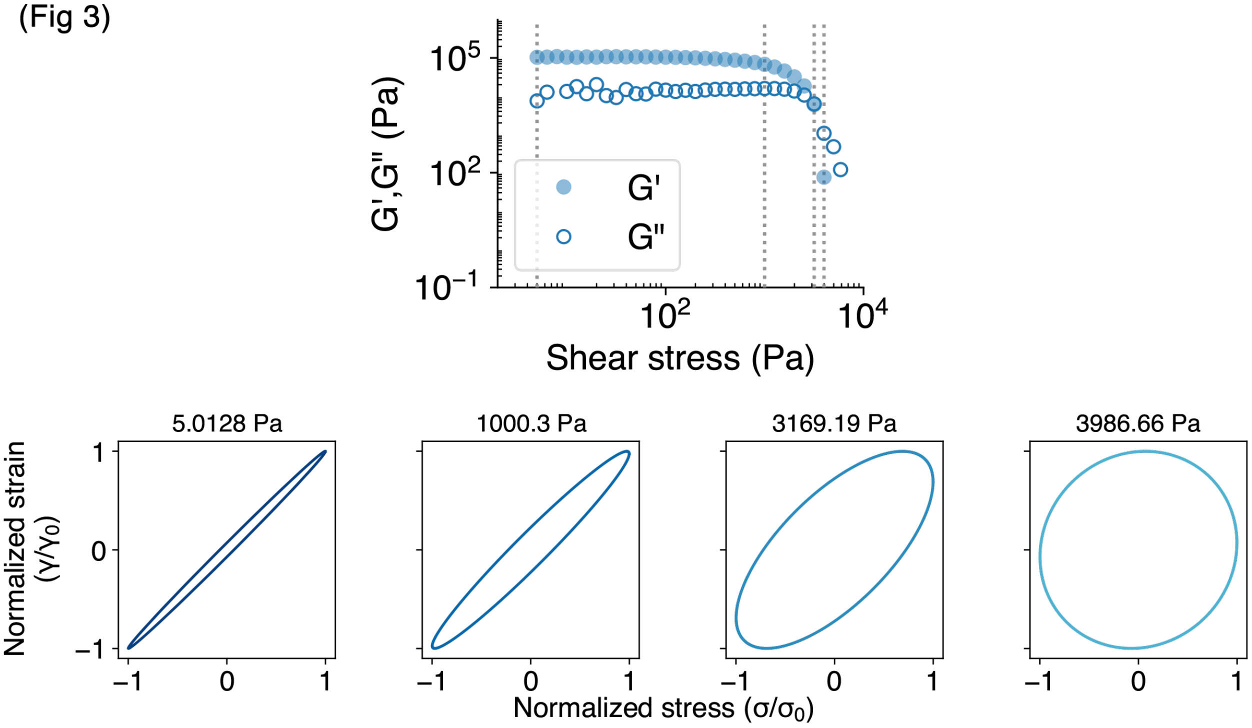

Sample B

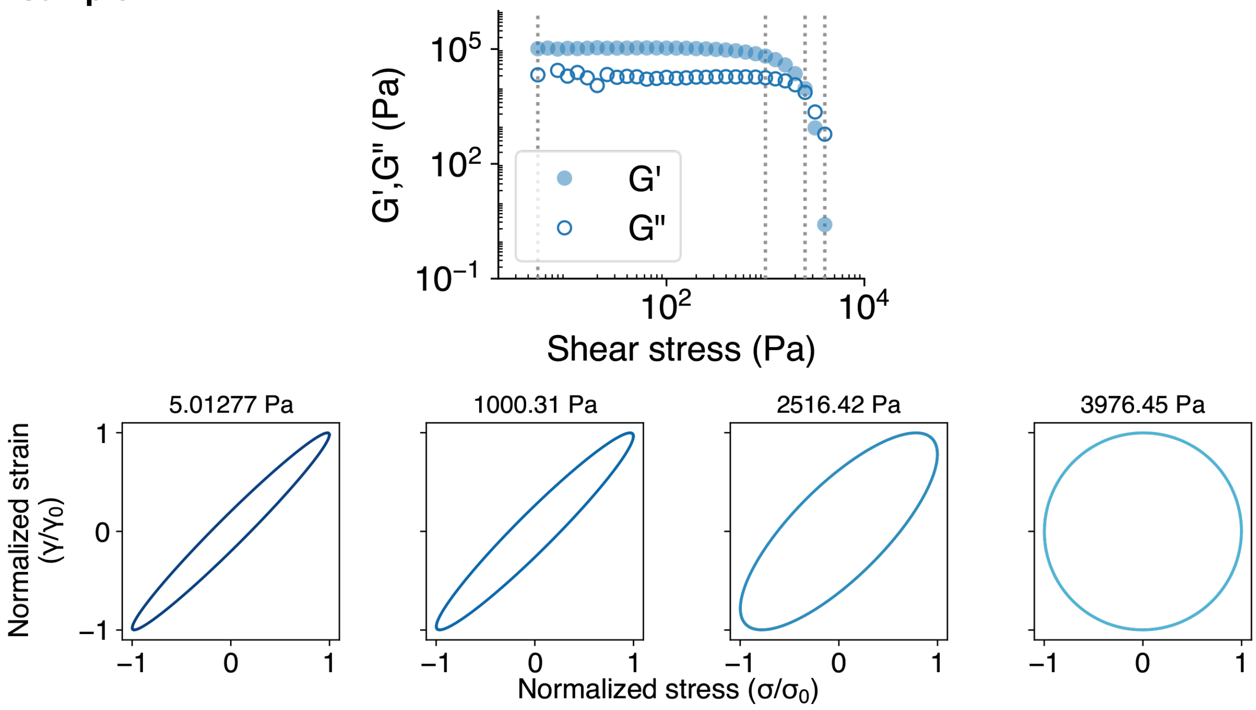

Figure S23: Lissajous plots from controlled stress oscillation experiments showing that the emulsion has a largely elastic behavior at low shear stress. After high stress above the yield stress, close to perfect viscous behavior (circular Lissajous plot) can be seen. The data shown for both samples represents an ink tested at 15.2% relative humidity.

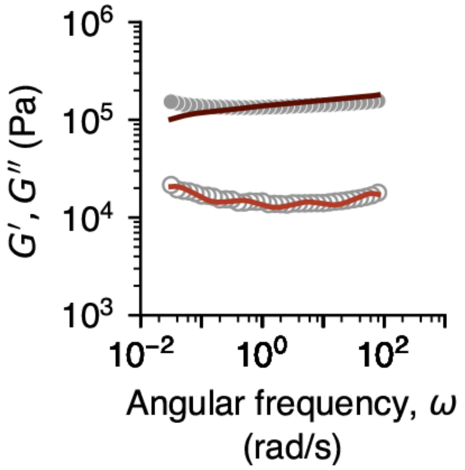

Figure S24: Oscillation frequency sweep of emulsion ink.

Supplementary Note 10: Oscillation Frequency Sweep of Optimal LME

Figure S24 shows a plateau G' vs. angular freq and is consistent with oscillation sweep data collected for highly concentrated emulsions concentration. [40],[34]. The data was fit to the discrete form of the relaxation modulus, $G(t)$, with four elements (*i.e.*, $N = 4$) to the G' and G'' data using the following equation[41]:

$$G'(\omega) = G_e + \sum_{i=1}^{4} g_i \frac{(\omega\lambda_i)^2}{1 + (\omega\lambda_i)^2} \tag{S42}$$

$$G''(\omega) = \sum_{i=1}^{4} g_i \frac{\omega\lambda_i}{1 + (\omega\lambda_i)^2} \tag{S43}$$

where G_e is the equilibrium modulus (where $t \to \infty$) and is calculated to be 8.29 $\times 10^4$ Pa, g_i is characteristic modulus, ω is the angular frequency, λ is the relaxation time. The following table shows results of the model fitting.

Table S8: Relaxation modulus and times obtained from model fit of **Equation S42**.

i	g (Pa)	λ (s)
1	3.18×10^4	0.014
2	2.00×10^4	0.187
3	2.08×10^4	2.081
4	3.84×10^4	30.37

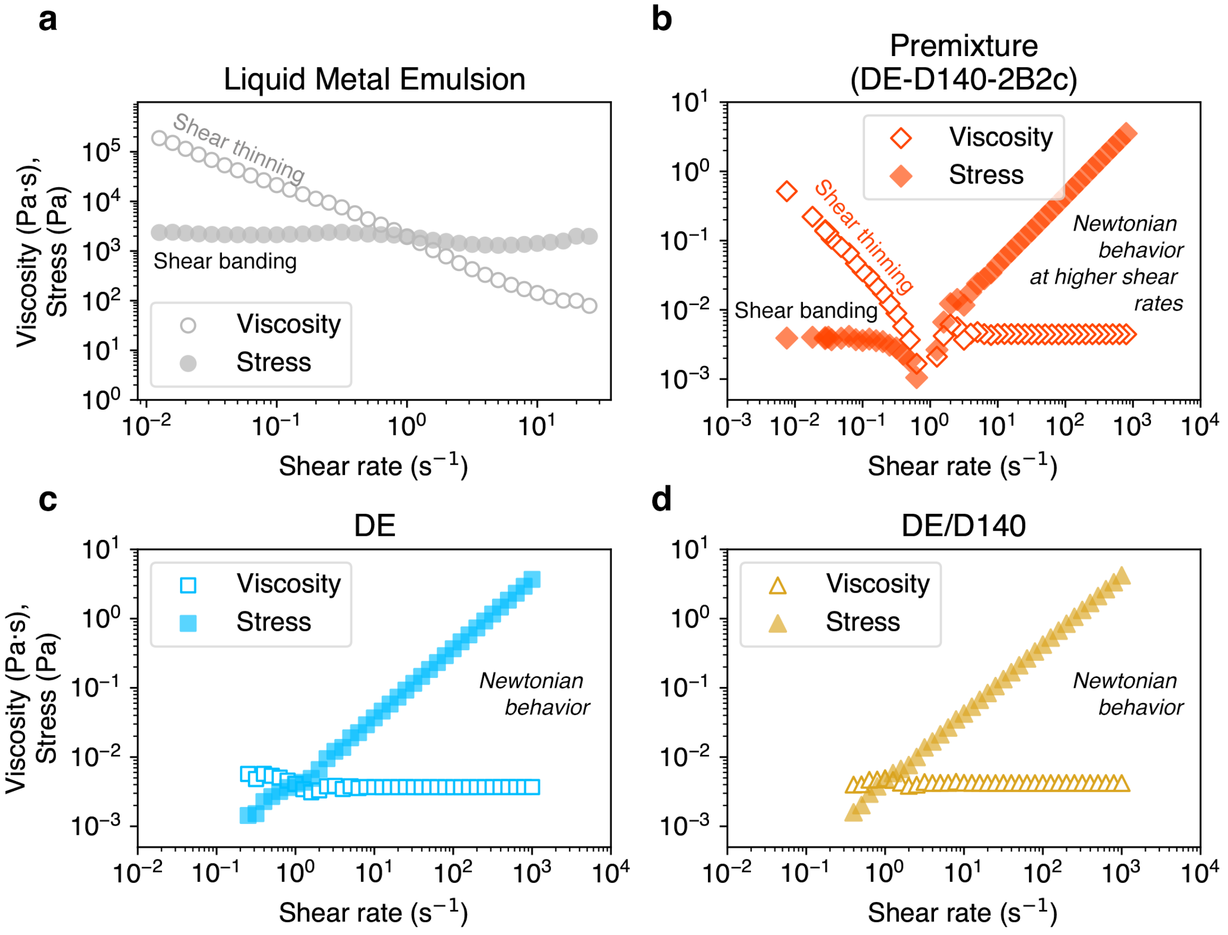

Figure S25: Flow curves for liquid metal emulsion and premixture. Viscosity data is represented by white with colored outline and accompanying stress curves are represented by filled in symbols for each dataset. (a) The liquid metal emulsion (LME) flow curve shows shear banding across the ∼3 decades of shear rates tested, which is consistent with past observations for highly concentrated emulsions.[42] (b) Flow curves for the premixture (DE-D140-2B2c), with their concentrations as present in the actual emulsion ink. The premixture is the only combination that displayed shear thinning and corresponding shear banding behavior, then transitions to having Newtonian behavior. (c) DE flow curves, and (d) and DE-D140 (concentration used in premixture and LME). The DE and DE-D140 flow curves display Newtonian behavior, with constant viscosity across all shear rates and linear stress curve. A stable signal for DE and DE-D140 below $\dot{\gamma} \sim 0.1$ s^{-1} could not be reliably measured by the instrument and is not available.

Supplementary Note 11: Gravitational and inertial effects on filament extrusion

The characteristic height at which gravity can affect the ink extrusion behavior is given by:[43]

$$H_{\text{vg}} = \left(\frac{\eta_0 Q}{\rho g}\right)^{1/4} \tag{S44}$$

and was calculated to be $H_{\text{vg}} = 10$ mm. This calculation utilized the following values for the equation above: the zero shear viscosity, $\eta_0 = 181.17$ kPa·s (measured from rheology flow experiments), the volumetric flow $Q = 2.89 \times 10^{-9}$ m^3 s^{-1} (calculated through $Q = \pi C(\alpha D)^2/4$, where C=88.912 mm s^{-1}, α= 1.017, [obtained from ink extrusion experiments] and $D = 0.2$ mm), the ink density $\rho = 5239.22$ kg m^{-3} and the gravitation acceleration $g = 9.81$ m s^{-2}.

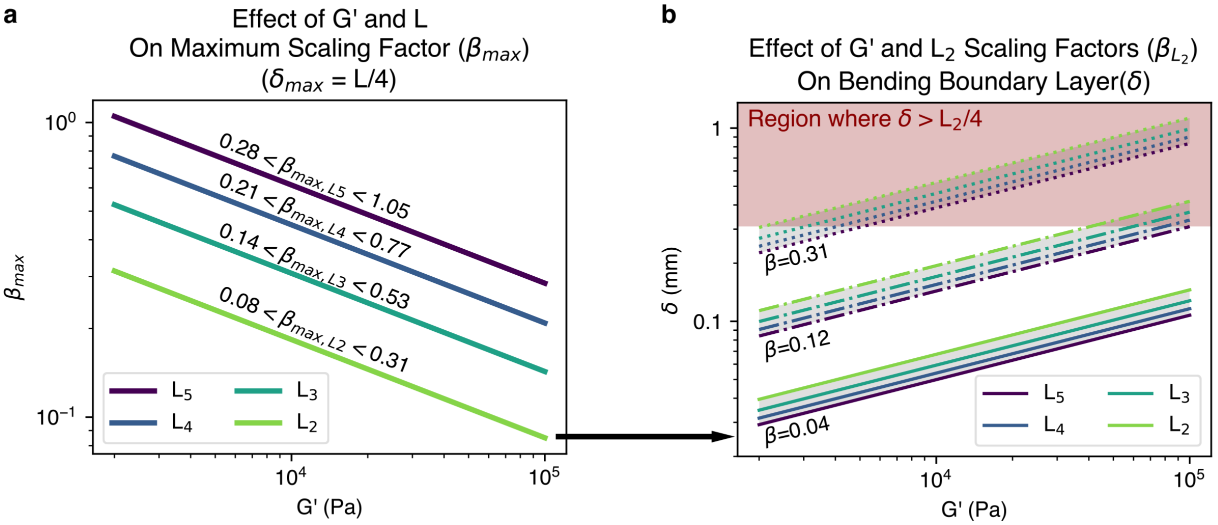

Figure S26: Estimation of scaling factor, β, for viscoelastic spanning model, based on spanning lines shown in **Figure 3f**. The lengths (L) of the lines with ID L_2, L_3, L_4, and L_5 are 1.2, 1.8, 2.4, and 3.1 mm, respectively. (a) Effect of the storage modulus, G' and length L of the spanning line on the calculated maximum values of the scaling factor, β_{max}, using **Equation S45**. (b) Calculated values for δ using β values within the calculated range shown in (a), showing how higher β can result in δ values that violate experimental results.

Supplementary Note 12: Estimation of bending boundary layer, δ

In order to estimate G' through **Equation 3**, δ must be fixed. The bending boundary layer for spanning viscoelastic lines scales through the equation $\delta \sim (\frac{Ed^5}{WL})^{1/3}$. We rewrite this scaling equation as:

$$\delta = \beta \left(\frac{Ed^5}{WL}\right)^{1/3} = \beta \left(\frac{3G'd^5}{WL}\right)^{1/3} \tag{S45}$$

introducing β as a scaling factor in order to calculate an actual value for δ for each spanned line, which is needed for estimating G'. To make sense of typical β values to fit, maximum beta ($\beta_{\max}$) for each line was found by assuming $\delta < 0.25L$, based on the end-bending catenary shape of the experimental data. This definition transforms **Equation S45** to:

$$\delta_{max} = \beta_{max} \left(\frac{3G'd^5}{WL} \right)^{1/3} \tag{S46}$$

$$\frac{L}{4} = \beta_{max} \left(\frac{3G'd^5}{WL} \right)^{1/3} \tag{S47}$$

$$\beta_{max} = \frac{1}{4} \left(\frac{WL^4}{3G'd^5} \right)^{1/3} \tag{S48}$$

Figure S26a shows that $\beta_{\max}$ varies depending on the chosen G' and L. To further narrow our search for β values, we plot δ vs G' and L using Equation (S45) using $\beta = 0.04, 0.12$ and 0.31 in **Figure S26b**, values that fall in the range of $\beta_{\max}$ for L_2, the shortest spanned line. As seen in Figure S26b, choosing β values of 0.12 and 0.31 would provide $\delta > 0.25L_2$ (shaded red region). Thus, we limited our search to $\beta <\sim 0.1$. Since it is known that a time dependent viscoelastic filament is has a bending boundary layer that approximately scales with $t^{-1/3}$,[44],[45], we utilized a value of $\beta = (3600)^{-1/3} = 0.065$ since ~1 hour elapsed between printing and 3D scanning.

Table S9: Calculated Constants used in **Equations 3, S45**, and **S48**.

**Constant**	**Description**	**Equation**	**Value**	
W	Distributed load	$0.25(\rho_{ink} - \rho_{air})g_0\pi d^2$	1.61×10^{-3}	N/m
ρ_{ink}	Ink density	$\left(\frac{w_{\text{eGaIn}}}{\rho_{\text{eGaIn}}} + \frac{w_{\text{DE}}}{\rho_{\text{DE}}} + \frac{w_{\text{D140}}}{\rho_{\text{D140}}} + \frac{w_{\text{2B2c}}}{\rho_{\text{2B2c}}}\right)^{-1}$	5239.223	kg/m^3
I	Area moment of inertia	$\frac{\pi d^4}{64}$	7.85×10^{-17}	m^4
A	Filament cross sectional area	$\pi(d/2)^2$	3.14×10^{-8}	m^2

Table S10: Constants used in **Table S9, Equations 3, S45** and **S48**.

**Constant**	**Description**	**Value**	
w_{eGaIn}	EGaIn mass fraction	0.954	
w_{DE}	Diphenyl ether mass fraction	0.031	
w_{D140}	D140 rosin mass fraction	0.001	
w_{2B2c}	2B2c mass fraction	0.014	
ρ_{eGaIn}	EGaIn density	6250	kg/m^3
ρ_{DE}	Diphenyl ether density	1080	kg/m^3
ρ_{D140}	D140 rosin density	1034	kg/m^3
ρ_{2B2c}	2B2c density	1602	kg/m^3
ρ_{air}	Air density	1.225	kg/m^3
g_0	Gravitational constant	9.8	m/s^2
d	Filament diameter	0.0002	m

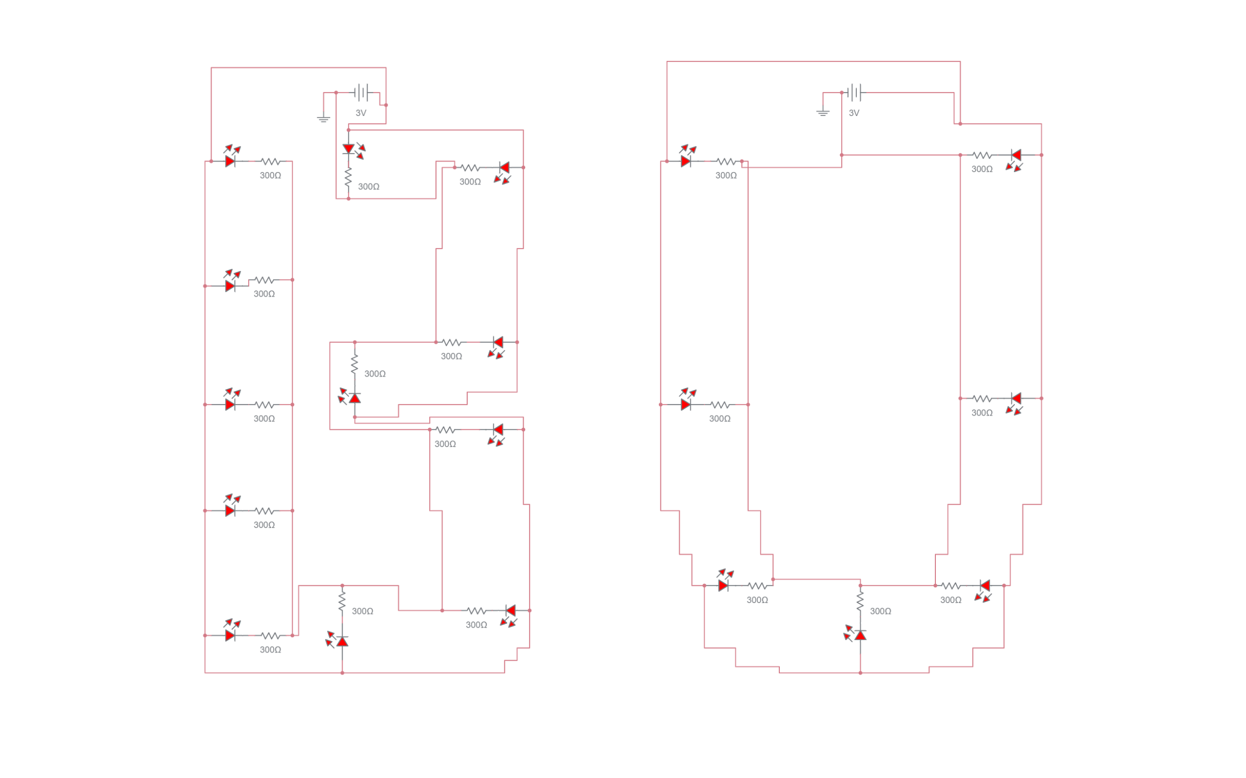

Figure S27: Circuit diagram for BU LED array presented in **Figure 4**.

Printing liquid metal emulsion (LME) ink on top of surface mount device (SMD) electrical pins results in variable electrical connection

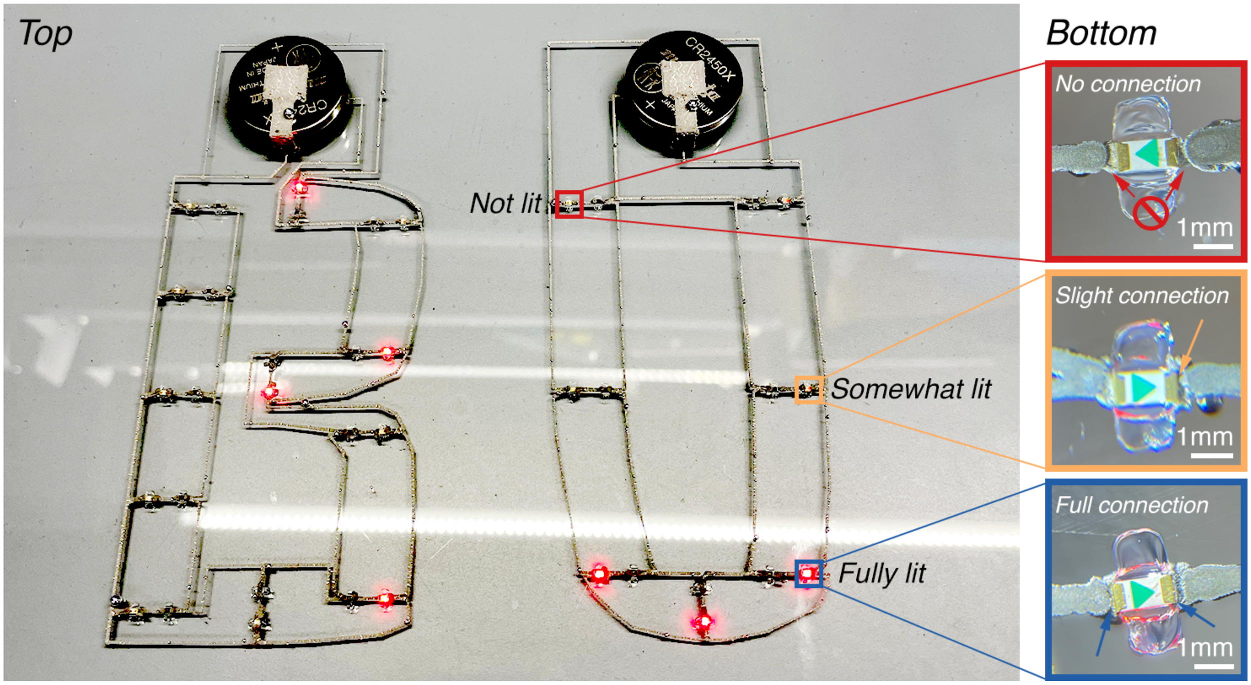

Figure S28: Printing LME on top of surface mount device electrical pins results in variable electrical connection, evidenced by inconsistent lighting of LED array.

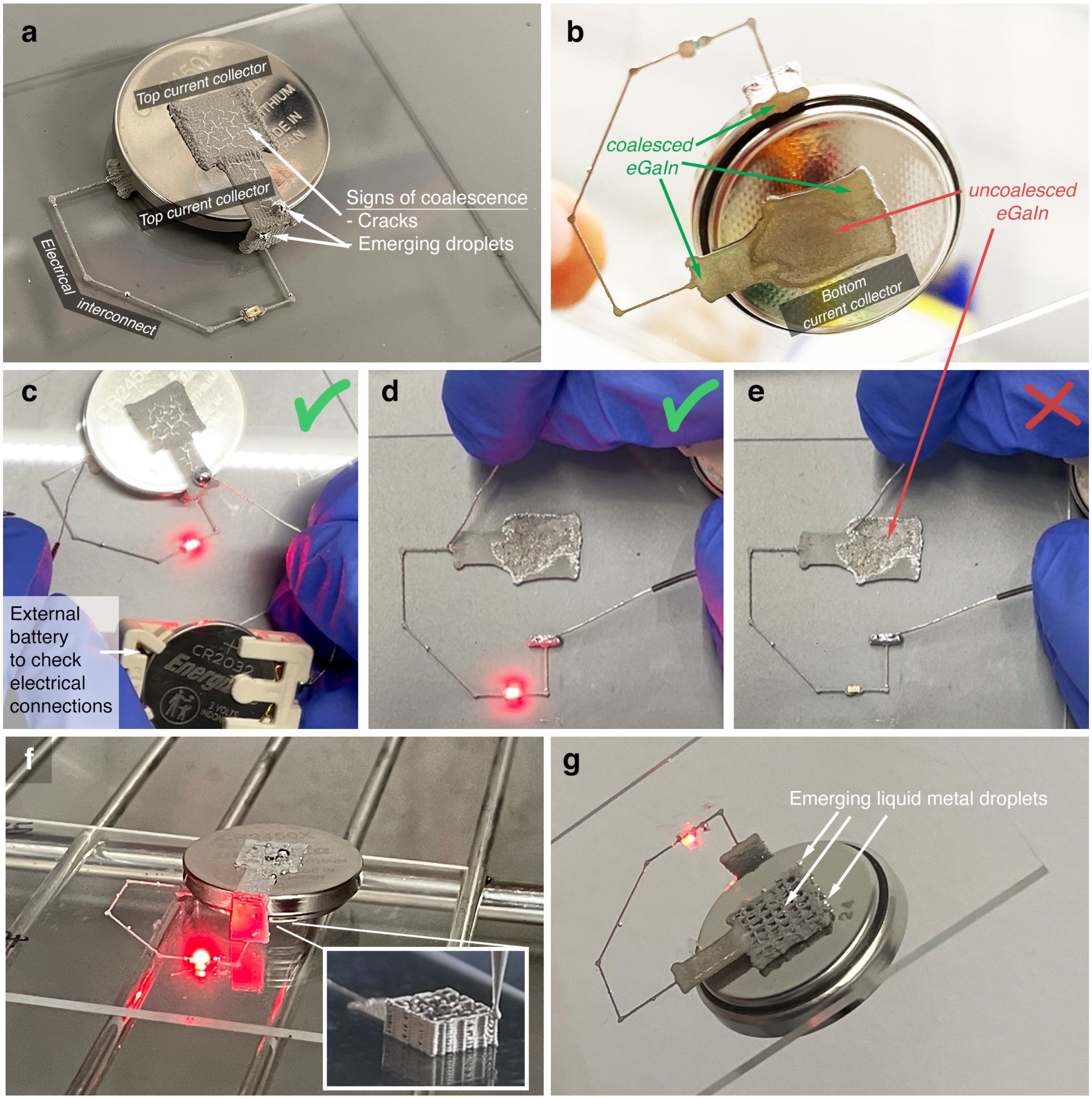

Figure S29: Design rationale for bottom lattice current collector. (a) Top of single LED device printed with liquid metal emulsion (LME) ink using a $\sim 200 \mu m$ thick square geometry after being heated at $T = 80°C$. Chemical coalescence is apparent from cracks and emerging eGaIn droplets. (b) Bottom of device shown in (a), where incomplete coalescence occurred, apparent by different color. (c)-(e) Conductivity of printed LME geometries are tested with an external coin cell battery and thin wire leads. If the LED lights up, the LME trace is conductive. (c) The connection between the top current collector and the end of the negative electrical interconnect is conductive, lighting up the LED. For (d)-(e), we removed the coin cell battery. (d) The connection between the vertical interconnect and the end of negative terminal of the electrical interconnect is conductive, lighting up the LED. (e) The connection between the vertical interconnect and the bottom current collector did not light up the LED. The lighting up of the LED in (d) as well as the reduced appearance of bulk eGaIn show the lack of coalescence with the flat square design. (f) Single LED device activated in $T = 80°C$ with the redesign of the bottom current collector to be a square lattice (printing shown in the inset). The LED lights up, indicating coalescence occurred throughout the print. (g) Bottom side of activated single LED device in (a), showing emerging eGaIn droplets (evidence of successful coalescence).

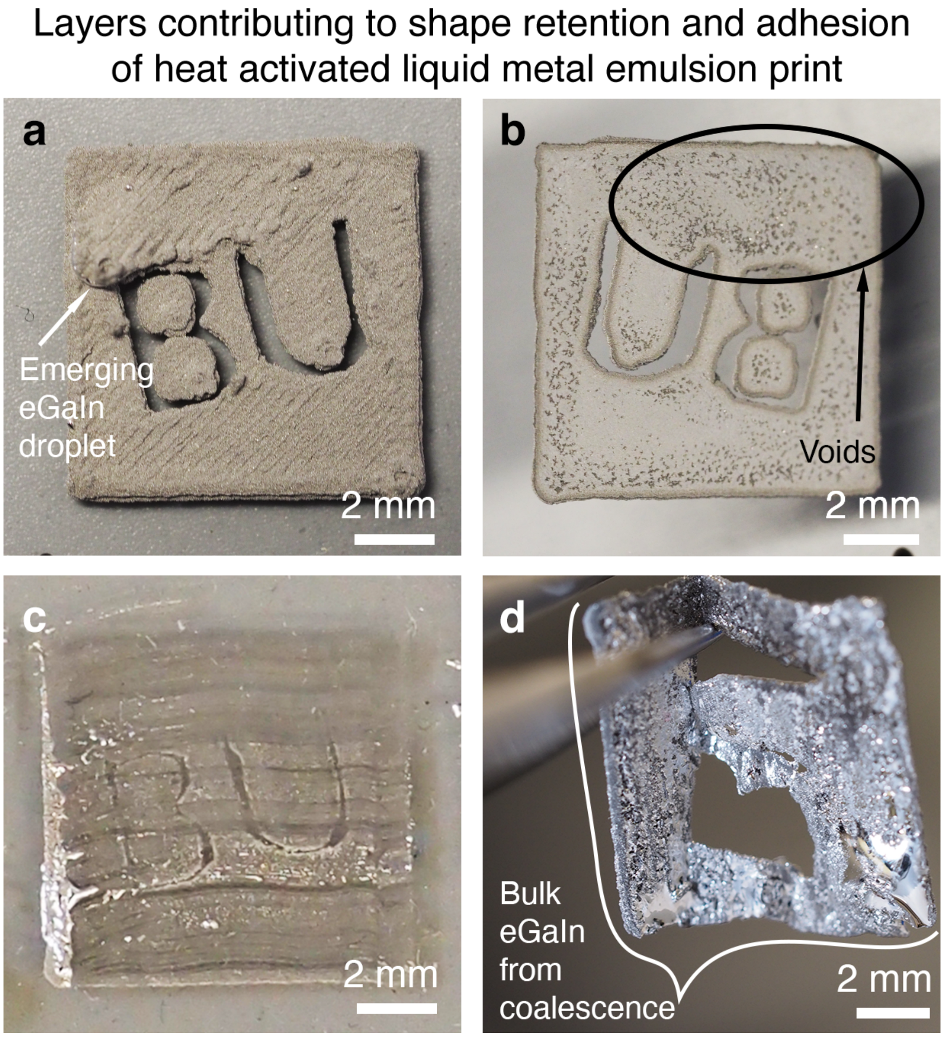

Figure S30: Structural layers that contribute to shape retention and adhesion in activated LMEs. (a) Top of activated 3D printed BU logo on glass, which shows a light gray, bumpy crust of uncoalesced eGaIn droplets. An emerging eGaIn droplet is sign that coalescence occurred. (b) Bottom side of (a). Opaque layer and voids suggest formation of oxide on the substrate. (c) Residue left over from glass slide of removed print. (d) Printed logo removed from the glass substrate showing structural integrity.

Supplementary Note 13: Estimation of adhesive stress for coalesced LME on SMD components

The adhesive stress on the battery ($\tau_{\text{s, battery}}$) is calculated as follows (using variables listed in **Table S11**):

$$\tau_{\text{s, battery}} = F/A_{\text{c,battery}}$$

where F is the weight of the battery given by:

$$\begin{aligned} F &= m_{\text{battery}} g \\ &= (0.062\text{kg})(9.8\text{m s}^{-2}) \\ &= \mathbf{0.61}\text{N} \end{aligned}$$

$A_{\text{c,battery}}$ is the contact area between the battery and LME. Since the bottom electrode is a lattice design with 7 filaments printed in each direction, we used the following equation to calculate the contact area:

$$\begin{aligned} A_{\text{c,battery}} &= \text{(total area of printed filaments) - (area of filament cross points)} \\ A_{\text{c,battery}} &= (n_{\text{filament}} \times (l_{\text{filament}} w_{\text{filament}})) - ((0.5 n_{\text{filament}})^2 \times (w_{\text{filament}})^2) \\ &= \mathbf{2.8 \times 10^{-7}}\text{m}^{\mathbf{2}} \end{aligned}$$

Thus, $\tau_{\text{s, battery}}$ is:

$$\begin{aligned} \tau_{\text{s, battery}} &= F/A_{\text{c,battery}} \\ \tau_{\text{s, battery}} &= 0.61\text{N}/(2.8 \times 10^{-7}\text{m}^2) \\ &= \mathbf{2.2 \times 10^{6}}\text{Pa} \end{aligned}$$

Table S11: Variables and values used for calculating $\tau_{\text{s, battery}}$

**Variable** (Units)	**Description**	**Value**
m_{battery} (kg)	Mass of battery	6.2×10^{-2}
l_{filament} (m)	Single filament length	8.4×10^{-4}
w_{filament} (m)	Single filament width	2.0×10^{-4}
n_{filament}	Number of filaments printed	14

The adhesive stress on the LED ($\tau_{\text{s, LED}}$) is calculated as follows (using variables listed in **Table S12**):

$$\tau_{\text{s, LED}} = F/A_{\text{c,LED}}$$

where F is the weight of the LED given by:

$$\begin{aligned} F &= m_{\text{LED}} g \\ &= (1.8 \times 10^{-6}\text{kg})(9.8\text{m s}^{-2}) \\ &= \mathbf{1.8 \times 10^{-5}}\text{N} \end{aligned}$$

$A_{c,LED}$ is the contact area between the LED and printed LME. We used the following equation to calculate the contact area:

$$A_{c,LED} = 2 \times (\text{area of contact pad})$$
$$A_{c,LED} = 2 \times (l_{pad} \times w_{pad})$$
$$= \mathbf{4.8 \times 10^{-7}} \text{m}^{\mathbf{2}}$$

Thus, $\tau_{s,\ LED}$ is:

$$\tau_{s,\ LED} = F/A_{c,LED}$$
$$\tau_{s,\ LED} = 1.8 \times 10^{-5} \text{N}/(4.8 \times 10^{-7} \text{m}^2)$$
$$= \mathbf{3.7 \times 10^{1}} \text{Pa}$$

Table S12: Variables and values used for calculating $\tau_{s,\ LED}$

**Variable** (Units)	**Description**	**Value**
m_{LED} (kg)	Mass of LED	1.8×10^{-6}
l_{pad} (m)	Length of LED contact pad	8.0×10^{-4}
w_{pad} (m)	Width of LED contact pad	3.0×10^{-4}

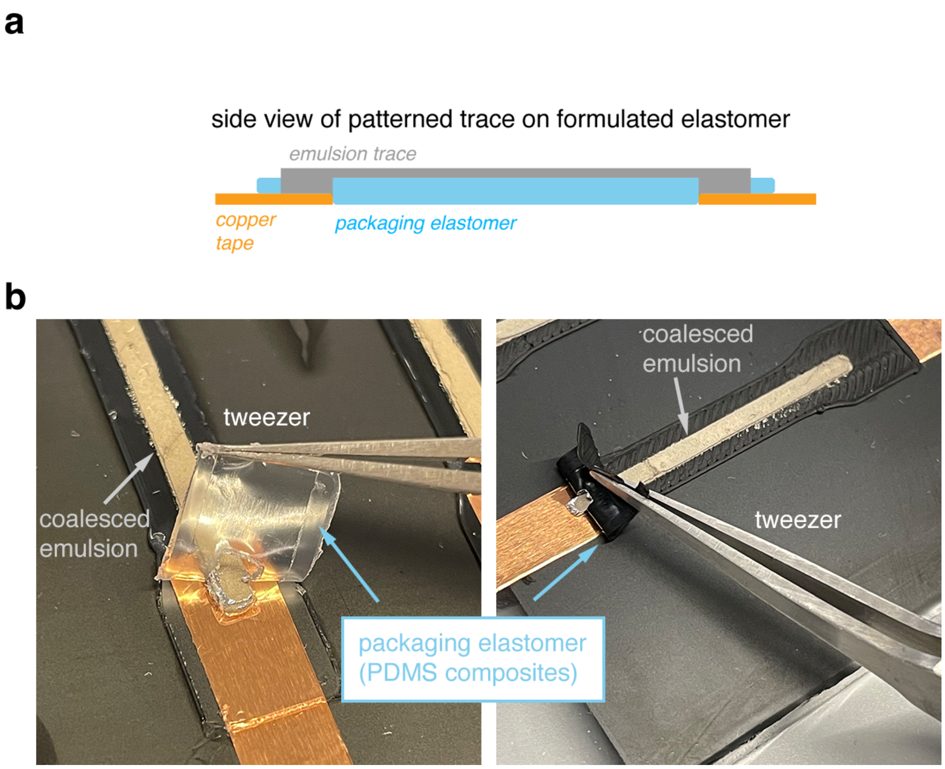

Figure S31: (a) Side schematic of stencil patterned emulsion trace on packaging elastomer based on PDMS. (b) Results of peeling PDMS elastomer composites after heat activating the emulsion traces. Left is PDMS/SiO_2 composite, right is PDMS/carbon black composite.

Supplementary Note 14: Calculation of LME conductivity after 10 months

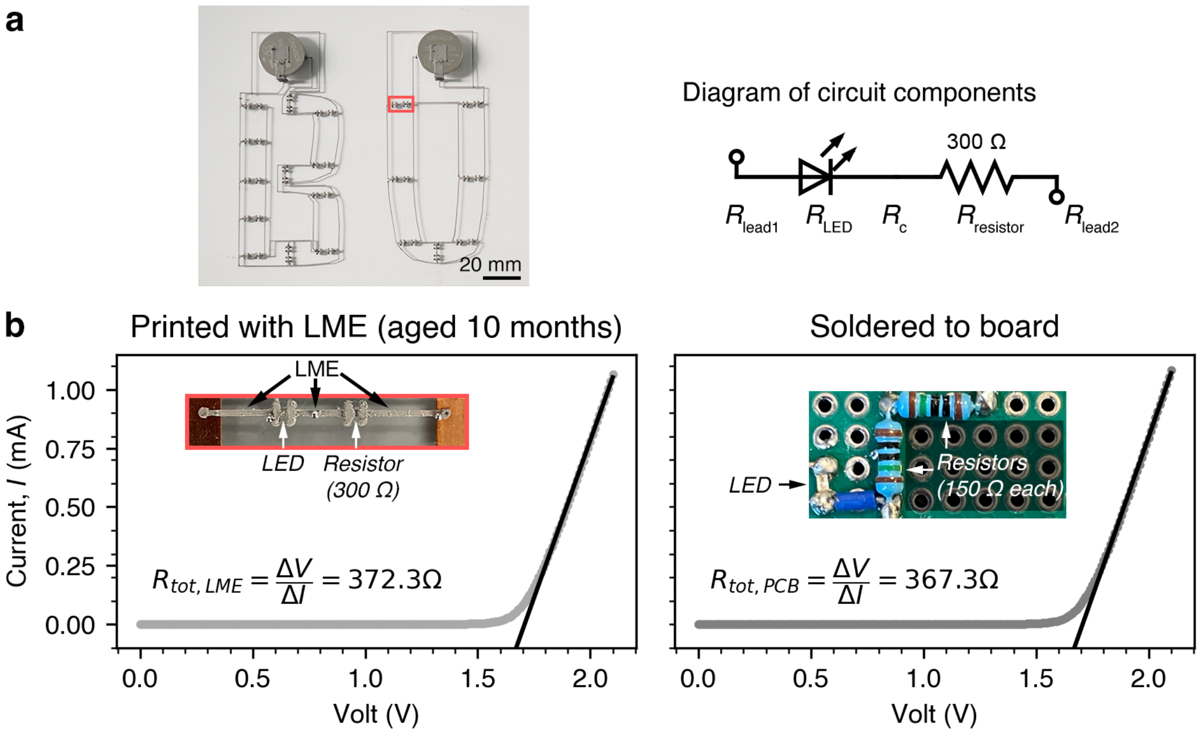

Figure S32: Comparison of 10-month aged LME to freshly soldered PCB using the same electrical components. (a) *Left.* BU LED array (adapted from manuscript Figure 4) with inset showing the circuit tested. The schematic of this circuit is shown on the right. (b) *I-V* data on 10 month old printed LME (left) compared to similar surface mount device components freshly soldered to a breadboard. The electrical contacts of the LED and resistors used in our BU LED array and in this figure are composed of a mix of copper/nickel/gold and nickel/tin, respectively.

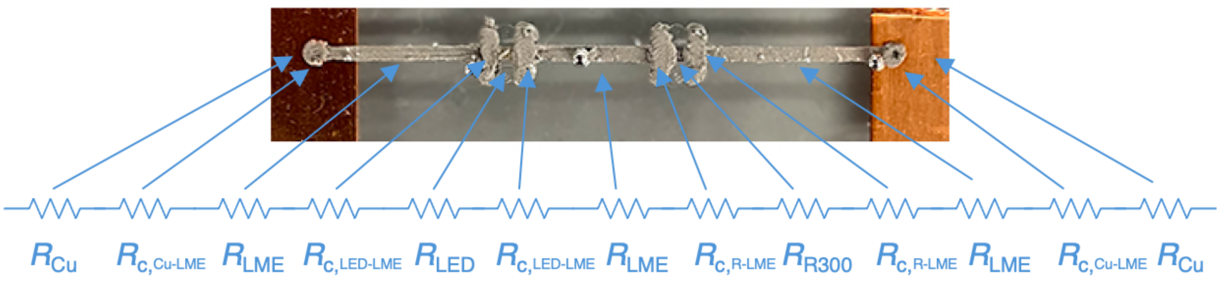

Figure S33: Circuit schematic used for calculating conductivity of 10-month aged LME

For the LME printed circuit shown in **Figure S33**, the total circuit can be represented by the equation for R_{tot} below:

$$\begin{aligned}
R_{\text{tot}} &= R_{\text{Cu}} + R_{\text{c,Cu-LME}} + R_{\text{LME}} + R_{\text{c,LED-LME}} + R_{\text{LED}} + R_{\text{c,LED-LME}} + R_{\text{LME}} + R_{\text{c,R-LME}} + R_{\text{R300}} \\
&\quad + R_{\text{c,R-LME}} + R_{\text{LME}} + R_{\text{c}} + R_{\text{Cu}} \\
R_{\text{tot}} &= 2R_{\text{Cu}} + 2R_{\text{c,Cu-LME}} + \cancel{2R_{\text{c,LED-LME}}} + \cancel{2R_{\text{c,R-LME}}} + 3R_{\text{LME}} + (R_{\text{LED}} + R_{\text{R300}}) \\
R_{\text{tot}} &= 2R_{\text{Cu}} + 2R_{\text{c,Cu-LME}} + 3R_{\text{LME}} + (R_{\text{LED}} + R_{\text{R300}})
\end{aligned}$$

where R_{Cu} is the copper (Cu) tape resistance, $R_{\text{c,Cu-LME}}$ is the contact resistance between the LME and Cu tape, R_{LME} is the LME resistance, R_{LED} is the LED 'resistance', $R_{\text{c,LED-LME}}$ is the contact resistance between the LME and LED, R_{R300} is the resistor resistance, and $R_{\text{c,R-LME}}$ is the contact resistance between the LME and the resistor. We assume $R_{\text{c,LED-LME}}$ and $R_{\text{c,R-LME}}$ are negligible compared to the resistances on the total circuit.

To estimate the conductivity of the 10-month aged LME ($\sigma_{\text{LME,aged}}$), we first rearrange for $R_{\text{LME,aged}}$ and use the calculated values from **Figure S32b** (i.e, $R_{\text{tot,aged}} = 372.3\Omega$ and $(R_{\text{LED}} + R_{\text{R300}}) = R_{\text{tot,PCB}} = 367.3\Omega$. We also use the measured value of $R_{\text{Cu}} = 0.004\Omega$ and assume $R_{\text{c,Cu-LME, aged}}$ is negligible:

$$\begin{aligned}
R_{\text{tot,aged}} &= 2R_{\text{Cu,aged}} + \cancel{2R_{\text{c,Cu-LME,aged}}} + 3R_{\text{LME,aged}} + (R_{\text{LED}} + R_{\text{R300}}) \\
R_{\text{LME,aged}} &= \frac{R_{\text{tot,aged}} - (2R_{\text{Cu,aged}} + (R_{\text{LED}} + R_{\text{R300}}))}{3} \\
&= \frac{(372.3 - (2 \times 0.004 + (367.3))}{3} \\
R_{\text{LME,aged}} &= 1.7\Omega
\end{aligned}$$

$\sigma_{\text{LME,aged}}$ can thus calculated by using definition for conductivity and printed LME interconnect geometry in the LME circuit. Below, l is the length of the interconnects printed between the copper tape to the LED, the LED to the resistor, and the resistor to the copper tape (**Figure S32b**); A is the printed interconnect cross section ($A = w_{\text{interconnect}} h_{\text{interconnect}}$):

$$\begin{aligned}
\sigma_{\text{LME,aged}} &= \frac{l}{AR_{\text{LME,aged}}} \\
&= \frac{0.6\text{cm}}{(0.0235\text{cm} \times 0.02\text{cm})(1.7\Omega)} \\
\sigma_{\text{LME,aged}} &= \mathbf{3.85 \times 10^{2}}\text{S cm}^{\mathbf{-1}}
\end{aligned}$$

The conductivity of a freshly activated LME is $\sigma_{\text{LME,fresh}} = 1.23 \times 10^{1}\text{S cm}^{-1}$ (**Figure S18**).

$$\begin{aligned}
\%\text{change} &= \frac{\sigma_{\text{LME,aged}} - \sigma_{\text{LME,fresh}}}{\sigma_{\text{LME,fresh}}} \times 100 \\
&= \frac{(3.85 \times 10^{2} - 1.23 \times 10^{1})}{1.23 \times 10^{1}} \times 100 \\
&= \mathbf{+30\%}
\end{aligned}$$

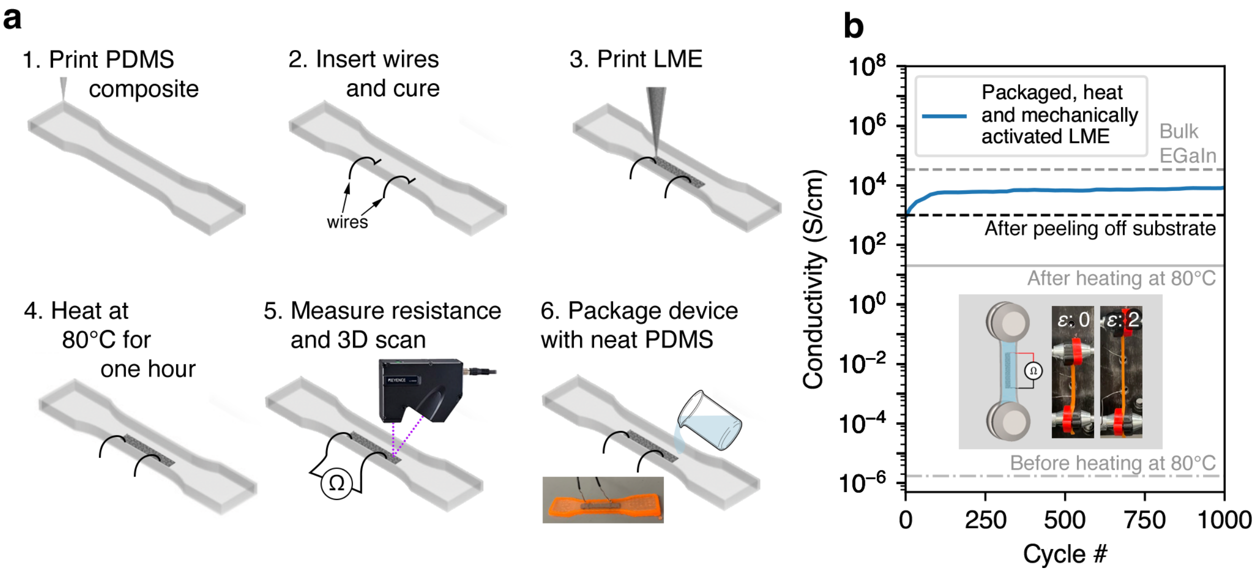

Figure S34: Printed LME packaged dogbone device and electromechanical testing. (a) Steps for fabricating packaged and activated liquid metal emulsion (optimum formulation). (b) Electromechanical test set-up and results

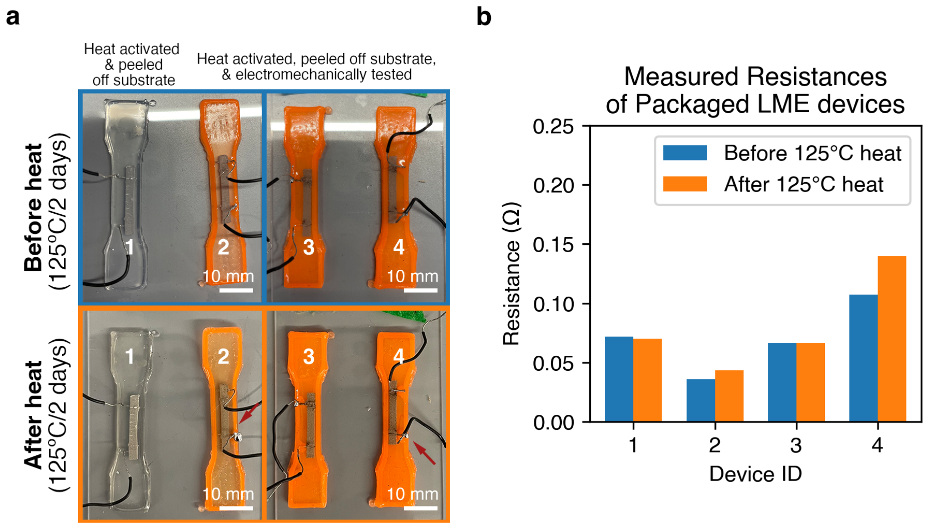

Figure S35: (a) Packaged dogbone devices before and after being heated at 125°C for 2 days. All devices but #1 were subjected to 200% stretching for 1000 cycles before being thermally tested. Droplets of eGaIn emerge in devices #2 and #4 after heat. (b) Measured resistances of devices shown in part (a)

Table S13: Alternate activators for chemically coalescing liquid metal emulsions and their associated carbon-halogen bond dissociation energy values (from Ref. [46], [47]). 2B2c is listed for reference.

**Halide Compound**	**Chemical Structure**	**Bond**	**Bond Dissociation Energy** (kcal/mol)
Chlorobenzene	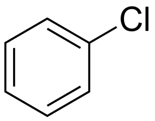	C-Cl	97.2
(2-Chloroethyl)benzene	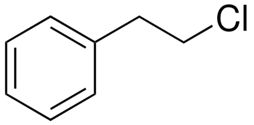	C-Cl	85.7
Benzyl chloride	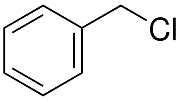	C-Cl	72.9
Cinnamyl chloride	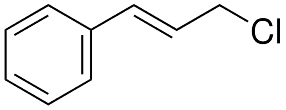	C-Cl	66.1
2-Bromoacetophenone	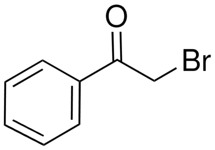	C-Br	64.2
2-Bromo-2'-chloroacetophenone (2B2c)	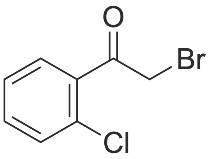	C-Br<br>C-Cl	63.1<br>91.5
2-Bromopropiophenone	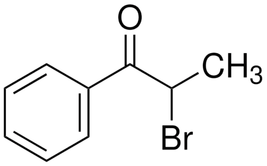	C-Br	60.4
Cinnamyl bromide	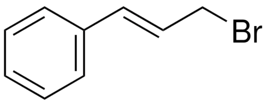	C-Br	53.5

Table S14: List of Supplementary Videos.

S1	Halide Salt-Based Liquid Metal Emulsions (LMEs) - Printing and Coalescing
S2	Print Velocity Sweep study of LME Extrusion
S3	Printing Spanning Filaments with LME
S4	LME-Based Device Activation at T $\sim$ 80°C
S5	Hybrid Assembly of BU LED Array with LME

References

[1] N.-C. Lee, *Reflow Soldering Processes and Troubleshooting*, chapter Solder Paste Technology, Newnes, **2002**.

[2] R. G. Pearson, *Journal of the American Chemical society* **1963**, *85*, 22 3533.

[3] Y. Ding, Z. Deng, C. Cai, Z. Yang, Y. Yang, J. Lu, Y. Gao, J. Liu, *International Journal of Thermophysics* **2017**, *38* 1.

[4] M. Zhang, L. Liu, C. Zhang, H. Chang, P. Zhang, W. Rao, *Adv. Mater. Interfaces* **2021**, *8*, 12 2100432.

[5] F. Krisnadi, S. Kim, S. Im, D. Chacko, M. H. Vong, K. Rykaczewski, S. Park, M. D. Dickey, *Adv. Mater.* **2024**, 2308862.

[6] T. Daeneke, K. Khoshmanesh, N. Mahmood, I. A. De Castro, D. Esrafilzadeh, S. J. Barrow, M. D. Dickey, K. Kalantar-Zadeh, *Chem. Soc. Rev.* **2018**, *47*, 11 4073.

[7] P. Van Gaans, H. Oonk, G. Somsen, *Journal of solution chemistry* **1990**, *19* 831.

[8] P. Smirnov, H. Wakita, M. Nomura, T. Yamaguchi, *Journal of solution chemistry* **2004**, *33* 903.

[9] C. Da Silva, O. Proux, J.-L. Hazemann, J. James-Smith, D. Testemale, T. Yamaguchi, *Journal of Molecular Liquids* **2009**, *147*, 1-2 83.

[10] L. Hepler, Z. Z. Hugus Jr, *Journal of the American Chemical Society* **1952**, *74*, 23 6115.

[11] T. J. Wadas, E. H. Wong, G. R. Weisman, C. J. Anderson, *Chemical reviews* **2010**, *110*, 5 2858.

[12] G. J. Gores, C. E. Koeppe, D. E. Bartak, *The Journal of Organic Chemistry* **1979**, *44*, 3 380.

[13] F. Alonso, I. P. Beletskaya, M. Yus, *Chemical Reviews* **2002**, *102*, 11 4009, pMID: 12428984.

[14] G. R. Fulmer, A. J. Miller, N. H. Sherden, H. E. Gottlieb, A. Nudelman, B. M. Stoltz, J. E. Bercaw, K. I. Goldberg, *Organometallics* **2010**, *29*, 9 2176.

[15] X. Li, M. Li, J. Xu, J. You, Z. Yang, C. Li, *Nat. Commun.* **2019**, *10*, 1 3514.

[16] W. M. Haynes, *CRC handbook of chemistry and physics*, CRC press, Boca Raton, FL, **2014**.

[17] G. S. Pokrovski, I. I. Diakonov, P. Bénézeth, J. Dandurand, J. Schott, V. Gurevich, K. Gavrichev, V. Gorbunov, I. L. Khodakovsky **1997**.

[18] Y. Lin, C. Cooper, M. Wang, J. J. Adams, J. Genzer, M. D. Dickey, *Small* **2015**, *11*, 48 6397.

[19] J. Park, S. Wang, M. Li, C. Ahn, J. K. Hyun, D. S. Kim, D. K. Kim, J. A. Rogers, Y. Huang, S. Jeon, *Nat. Commun.* **2012**, *3*, 1 916.

[20] S. Zhu, J.-H. So, R. Mays, S. Desai, W. R. Barnes, B. Pourdeyhimi, M. D. Dickey, *Advanced Functional Materials* **2013**, *23*, 18 2308.

[21] Y. Jo, J. H. Hwang, S. S. Lee, S. Y. Lee, Y. S. Kim, D.-G. Kim, Y. Choi, S. Jeong, *ACS Appl. Mater. Inter.* **2022**, *14*, 8 10747.

[22] J. Wang, G. Cai, S. Li, D. Gao, J. Xiong, P. S. Lee, *Adv. Mater.* **2018**, *30*, 16 1706157.

[23] Z. Ma, Q. Huang, Q. Xu, Q. Zhuang, X. Zhao, Y. Yang, H. Qiu, Z. Yang, C. Wang, Y. Chai, et al., *Nat. Mater.* **2021**, *20*, 6 859.

[24] U. Daalkhaijav, O. D. Yirmibesoglu, S. Walker, Y. Mengüç, *Adv. Mater. technologies* **2018**, *3*, 4 1700351.

[25] M. C. Yuen, M. A. Creighton, C. E. Tabor, In *2020 3rd IEEE International Conference on Soft Robotics (RoboSoft)*. IEEE, **2020** 676–681.

[26] S. Liu, M. C. Yuen, E. L. White, J. W. Boley, B. Deng, G. J. Cheng, R. Kramer-Bottiglio, *ACS Appl. Mater. Inter.* **2018**, *10*, 33 28232.

[27] Z. Lin, X. Qiu, Z. Cai, J. Li, Y. Zhao, X. Lin, J. Zhang, X. Hu, H. Bai, *Nat. Commun.* **2024**, *15*, 1 4806.

[28] S. Liu, D. S. Shah, R. Kramer-Bottiglio, *Nat. Mater.* **2021**, *20*, 6 851.

[29] W. Lee, H. Kim, I. Kang, H. Park, J. Jung, H. Lee, H. Park, J. S. Park, J. M. Yuk, S. Ryu, et al., *Science* **2022**, *378*, 6620 637.

[30] J. W. Boley, E. L. White, R. K. Kramer, *Adv. Mater.* **2015**, *27*, 14 2355.

[31] A. Fassler, C. Majidi, *Adv. Mater* **2015**, *27*, 11 1928.

[32] M. G. Mohammed, R. Kramer, *Adv. Mater.* **2017**, *29*, 19 1604965.

[33] C. J. Thrasher, Z. J. Farrell, N. J. Morris, C. L. Willey, C. E. Tabor, *Adv. Mater.* **2019**, *31*, 40 1903864.

[34] T. Neumann, E. Facchine, B. Leonardo, S. Khan, M. Dickey, *Soft Matter* **2020**, *16*, 28 6608–6618.

[35] R. Sánchez Cruz, S. F. Zopf, J. W. Boley, *J. Compos. Mater.* **2023**, *57*, 4 829–839.

[36] M. A. Fischler, R. C. Bolles, *Communications of the ACM* **1981**, *24*, 6 381.

[37] M. Ester, H.-P. Kriegel, J. Sander, X. Xu, et al., In *kdd*, volume 96. **1996** 226–231.

[38] H. Berger, *Solid-state electronics* **1972**, *15*, 2 145.

[39] G. Reeves, H. Harrison, *IEEE Electron device letters* **1982**, *3*, 5 111.

[40] T. Mason, J. Bibette, D. Weitz, *Phys. Rev. Lett.* **1995**, *75*, 10 2051.

[41] M. Baumgaertel, H. Winter, *Rheologica Acta* **1989**, *28* 511.

[42] T. Mason, J. Bibette, D. Weitz, *Journal of colloid and interface science* **1996**, *179*, 2 439.

[43] H. Yuk, X. Zhao, *Adv. Mater.* **2018**, *30*, 6.

[44] J. Teichman, L. Mahadevan, *J. Fluid Mech.* **2003**, *478* 71.

[45] A. Roy, L. Mahadevan, J.-L. Thiffeault, *J. Fluid Mech.* **2006**, *563* 283.

[46] P. C. St. John, Y. Guan, Y. Kim, S. Kim, R. S. Paton, *Nature communications* **2020**, *11*, 1 2328.

[47] P. C. St. John, Y. Guan, Y. Kim, B. D. Etz, S. Kim, R. S. Paton, *Sci. Data* **2020**, *7*, 1 244.
